# Shexiang Tongxin Dropping Pills Promote Macrophage Polarization-Induced Angiogenesis Against Coronary Microvascular Dysfunction *via* PI3K/Akt/mTORC1 Pathway

**DOI:** 10.3389/fphar.2022.840521

**Published:** 2022-03-23

**Authors:** Xiangyu Lu, Junkai Yao, Changxiang Li, Lingwen Cui, Yizhou Liu, Xiangning Liu, Gang Wang, Jianteng Dong, Qiong Deng, Yueyao Hu, Dongqing Guo, Wei Wang, Chun Li

**Affiliations:** ^1^ Modern Research Center for Traditional Chinese Medicine, School of Chinese Materia Medical, Beijing University of Chinese Medicine, Beijing, China; ^2^ Dongzhimen Hospital, Beijing University of Chinese Medicine, Beijing, China; ^3^ School of Traditional Chinese Medicine, Beijing University of Chinese Medicine, Beijing, China; ^4^ School of Life Sciences, Beijing University of Chinese Medicine, Beijing, China; ^5^ Key Laboratory of TCM Syndrome and Formula (Beijing University of Chinese Medicine), Ministry of Education, Beijing, China

**Keywords:** Shexiang Tongxin dropping pills, coronary microvascular dysfunction, M2 macrophages, angiogenesis, PI3K/AKT/mTORC1 pathway

## Abstract

**Background:** Accumulating evidence suggests that coronary microvascular dysfunction (CMD) is one of the important causes of coronary artery diseases. Angiogenesis can effectively improve CMD by increasing blood supply capacity, recovering cardiac function and poor hemodynamics. Clinical studies have approved Shexiang Tongxin dropping pill (STDP), which has exerted remarkable roles on ameliorating CMD, but the effects and mechanisms of STDPs on angiogenesis have not been clarified.

**Purpose:** The purpose of this study was to elucidate the effects and potential mechanisms of STDPs on macrophage polarization-induced angiogenesis against CMD.

**Methods:** Echocardiography, optical microangiography (OMAG), and histological examination were applied to evaluate cardioprotection and proangiogenic effects of STDPs on left anterior descending (LAD) ligation-induced CMD rats. *In vitro*, oxygen–glucose deprivation–reperfusion (OGD/R)-induced HUVEC model and LPS-stimulated bone marrow-derived macrophage (BMDM) model were established to observe the effects of STDPs on angiogenesis and M2 macrophage polarization.

**Results:** STDPs improved cardiac function, increased microvascular density, and the number of M2 macrophages in the heart of CMD rats. *In vitro*, STDPs accelerated the proliferation, migration, and tube formation in OGD/R-induced HUVECs similar to the effects of VEGF-A. Furthermore, in LPS-stimulated BMDMs model, STDPs modulated M2 macrophage polarization and increased VEGF-A release via the PI3K/AKT/mTORC1 pathway.

**Conclusion:** STDPs promoted macrophage polarization-induced angiogenesis against CMD *via* the PI3K/Akt/mTORC1 pathway. Our results demonstrated that the phenotype transformation of macrophages and stimulating the secretion of VEGF-A may be applied as novel cardioprotective targets for the treatment of CMD.

## Introduction

The coronary microcirculation system consists of arterioles, venules, and capillaries in the heart (Kellar et al., 2001). Coronary microvascular dysfunction (CMD) refers to the mismatch between myocardial blood supply and oxygen consumption, which is caused by abnormal coronary microvessels with diameters of less than 500 μm (Bender et al., 2016). Accumulating evidence suggests that CMD contributes to many coronary artery diseases (Chen et al., 2016). Coronary artery diseases were recognized as the main etiological factors in more than 50% of heart failure patients in North America and Europe (Pagliaro et al., 2020). Therefore, it is urgent to explore effective therapeutics to prevent CMD.

Diabetes, gender, aging, and inflammation are the main risk factors for CMD (Rensma et al., 2020). The potential mechanisms of CMD include: 1) microvascular remodeling, endothelial and smooth muscle dysfunction; 2) inflammatory response; 3) coronary microvascular spasm; and 4) abnormal hemorheological factors (Ca Mici and Filippo, 2014). Angiogenesis is one of the most important ways to improve CMD (Wu et al., 2017). The exact mechanisms involved in angiogenesis against CMD have not been fully elucidated. Angiogenesis is the process that sprouts the new blood vessels from existing vasculature and is essential to restore blood supply for the ischemic tissues (Pandya et al., 2006). The formation of collateral vessels is mainly from the marginal zone of the myocardial infarction into the infarcted heart tissue, and ultimately determines the functional and structural development of the heart (van der Laan et al., 2009). It is well known that angiogenesis is mainly regulated by proangiogenic cytokinases and growth factors (Shapouri-Moghaddam et al., 2018). For example, vascular endothelial growth factor-A (VEGF-A) is crucial to the angiogenesis process including degradation of basement membrane, induction of endothelial cells to migrate, proliferate, sprout, and tube formation (Liu et al., 2020). VEGF-A can be secreted by different cells in the heart, including macrophages, myocardial cells, endothelial cells, and so on (de la Torre et al., 2013; Icli et al., 2019). Macrophages are one of the most widely studied innate immune cells, which participate in myocardium angiogenesis (Mia et al., 2020). In response to environmental stimulation, macrophages exert phenotypic transformation and implement different functions (Liu et al., 2014). Macrophages can be divided into M1 macrophages and M2 macrophages according to their function and inflammatory factor secretion level (Chen et al., 2020). M1 macrophages secrete high-level TNF-α and low-level IL-10, which promote the occurrence and development of inflammation (Essandoh et al., 2016). However, M2 macrophages are characterized by secreting high levels of arginase-1 (Arg-1), IL-10, and VEGF-A, and play important roles in the process of angiogenesis and tissue repair (Peet et al., 2020). Thus, regulating M2 macrophage polarization may become a novel target to improve CMD and restore blood supply for the ischemic heart.

In the clinic, the treatment for CMD mainly focuses on drug treatment, including antiplatelet drugs, antiarrhythmic drugs, antiangina drugs, and calcium antagonists (Ong et al., 2016). However, increasing the risk of bleeding and other adverse reactions might exist (Luo et al., 2019). Shexiang Tongxin dropping pills (STDPs) were approved for marketing by the National Medical Products Administration in 2008, which consist of seven components: *Centaurea moschus* Hablitz, *Ginseng quinquefolium* (L.) Alph.Wood, *Bufonis Venenum*, *Salvia absconditiflora* Greuter and Burdet, *Bovis Calculus Artifactus*, *Fel Ursi*, and *Dryobalanops aromatica* C.F.Gaertn (Chen et al., 2015; Lin et al., 2017). STDPs are widely applied for the treatment of cardiovascular diseases in China (Lu et al., 2020), which have the effects of regulating qi and relieving pain, strengthening the heart, and activating collaterals (Pan et al., 2021). Surprisingly, STDPs have remarkable efficacy against CMD in the clinic (Tian et al., 2020). Clinical data have also reported that STDPs could inhibit inflammatory responses and, meanwhile, upregulate VEGF-A in the plasma of patients with coronary artery disease (Liu et al., 2021). Nevertheless, the mechanism of STDPs still remains unknown. The purpose of this study was to elucidate the effects and potential mechanisms of STDPs on macrophage polarization-induced angiogenesis against CMD.

## Materials and methods

### Drugs

STDPs were supplied by the Inner Mongolia Conba Pharmaceutical Co., Ltd. (Shanghai, China) and approved for marketing in 2008, with approval number: Z20080018. STDPs consist of seven active pharmaceutical ingredients, which was first dissolved in dimethyl sulfoxide (DMSO) with an initial concentration of 42 mg/ml (STDP:DMSO = 42 mg:1 ml). The resulting solution was autoclaved and stored at −20°C until further use. The fingerprint was established by HPLC to control the quality of STDPs **(** Supplementary Figure S1 **)**. LY294002 (154447-36-6, Selleck.cn, USA), the inhibitor of PI3K, was purchased from Selleck.co.uk. Telmisartan was indicated to be effective for patients with myocardial ischemia and used as a positive drug (J20090089, Boehringer Ingelheim, Germany). LPS (l2880, biodee, China) was purchased from the Beijing BioDee Biotechnology Co., Ltd.

### Animals and treatment

Experimental procedures were conducted in accordance with the China Physiological Society’s “Guiding Principles in the Care and Use of Animals,” and approved by the animal care committee of the Beijing University of Chinese Medicine (BUCM-4-2021113002-4048). Fifty Sprague–Dawley (SD) rats (220–240 g) were purchased from the Spelford (Beijing) Laboratory Animal Co., Ltd. All rats were on adaptive feeding for 3 days and fasted for 12 h before the surgery. The left anterior descending (LAD) artery of the rats were ligated to induce acute myocardial ischemia and CMD model as previously described (Wang et al., 2013). The rats were randomly divided into the sham group, model group, STDP group (21.6 mg/kg*day), and telmisartan group (8.23 mg/kg*day) for 7 days. An equal amount of saline was given by gavage in the other groups. Pentobarbital sodium was used as an anesthetic during the surgeries, and the greatest effort was made to minimize pain.

### Echocardiographic assessment

Rats were anesthetized and fixed on a wooden board. Cardiac function was assessed using an echocardiograph (Vevo TM 2100, Visual Sonics, Canada). The main parameters included the left ventricular ejection fraction (LVEF), short-axis shortening fraction (LVFS), left ventricular end-diastolic internal diameter (LVID; d), and left ventricular end-systolic internal diameter (LVID; s).

### Histological examination

Myocardial tissue from each group was fixed in 4% paraformaldehyde, and paraffin-embedded sections were prepared. Sections were stained with hematoxylin–eosin (HE) as previously described (Meng et al., 2021).

### Optical microangiography

In this study, OMAG imaging system (BaslerSPL 4096-140 km, Germany) based on optical coherent tomography (OCT) technology was used to evaluate the density of microvessels in the rat heart (Wan et al., 2016). Briefly, the spectral bandwidth was 100 nm, the central wavelength was 1,310 nm, and the axial resolution was 15 μm. The OCT system used an interframe ultra-high sensitivity OMAG algorithm to extract 3D vessels from images of cardiac tissue structures. The maximum intensity projection (MIP) of the volumetric vasculature allowed the morphological information of the microvascular network to be displayed from the top view (Redd et al., 2019). Each data volume took 1 min. The rat was fixed to a wooden board after anesthetization, and a small animal ventilator was attached to ensure that the rat could breathe normally. The heart was exposed by an opening between the third and fourth intercostal space. An appropriately sized cotton pad moistened with saline was placed under the heart to raise the heart slightly for the acquisition of microvascular imaging. The probe was placed in the same position in the heart, and the heart was held at pressures for image acquisition.

### Immunofluorescence analysis

Paraffin sections of heart sections were dewaxed and blocked with serum for 30 min. The sections were incubated overnight at 4°C with anti-CD206 antibody (ab201340, Abcam, USA) and anti-CD31 antibody (ab53004, Abcam, USA) in a wet box. Then the slides were washed with PBS three times. Secondary antibody (D001-34, Abcam, USA) was incubated for 50 min at room temperature in the dark. The nuclei were stained with DAPI (C0065, Solarbio, China).

Cells were seeded in confocal dishes, fixed with 4% paraformaldehyde for 15 min, and permeabilized with 0.5% TritonX-100 for 20 min. The cells were blocked with 1% BSA in a 37°C incubator for 1 h. Incubation with anti-CD206 antibody at 4°C overnight was followed by incubation with the anti-rabbit fluorescent secondary antibody IgG (AB0141, Abways, China) for 1 h at room temperature. Nuclei were stained with DAPI for 5 min.

### Western blot analysis

Rat heart tissues and cell lysates were prepared using RIPA lysis buffer containing protease inhibitor (c1053-100, Applygen, China), and phosphatase inhibitors (p1260-1, Applygen, China). An equal amount of protein was separated on 10%–15% SDS-polyacrylamide gels and transferred onto PVDF membranes. After blocking with 5% nonfat milk at room temperature for 2 h, the PVDF membranes were incubated with the appropriate primary antibody, followed by a secondary antibody. The PVDF membrane was exposed to ECL reagents (p1020-25, Applygen, China) to visualize the protein signals. The antibodies used for Western blot were as follows: anti-PI3K (1F6A7, Proteintech, USA), anti-Akt (2C5D1, Proteintech, USA), anti-Phospho–PI3K (CY6427, Abways, China), anti-Phospho–Akt (P31749, Abways, China), anti-VEGFA (ab 214424, Abcam, USA), anti-ARG-1 (P05089, Abways, China), anti-mTORC1 (Q6UUV9, Abways, China), anti-CD206 (JF0953, HuaAn, China), anti-rabbit IgG H and L (ab0101, Abways, China), and anti-mouse IgG H and L (ab0102, Abways, China).

### Troponin, IL-10, and vascular endothelial growth factor-A detection

The plasma levels of troponin (cTnI), IL-10, and VEGF-A were quantified using the ELISA kits (Wuhan Yunclone Technology Co., Ltd., Wuhan, China) according to the manufacturer’s instructions.

### Cell culture and group of HUVECs

HUVECs were obtained from the Beijing Anzhen Hospital. HUVECs were cultured with a 1640 medium containing 10% FBS and 1% penicillin/streptomycin (P/S) in a humidified incubator with 5% CO_2_ at 37°C. The groups were as follows: control group, model group, STDP group (100 ng/ml), and VEGF-A group (20 ng/ml). In the model group, oxygen–glucose deprivation–reperfusion (OGD/R) was constructed to induce cell injury. HUVECs were seeded at a density of 8 × 10^3^/well in 96-well plates. After 24 h, cells in the model group were incubated with Earle’s balanced salt solution in an anaerobic box for 6 h. Then the Earle’s balanced salt solution was removed, and 1640 medium was added to recover oxygen–glucose for 12 h at 37°C with 5% CO_2_. The cells in the STDP and VEGF-A groups underwent ODG/R simultaneously with STDPs or VEGF-A treatment.

### Cell viability assay

Cell viability was measured using CCK8 (Jiancheng, Nanjing, China) according to the manufacturer’s instructions. After different treatments, 10 µl of CCK8 was added to the well, and the plate was incubated in 37°C for 2 h. Then absorbance was measured using a microplate reader (PerkinElmer VICTOR1420, USA) at 450 nm.

### Wound healing assay

When HUVECs were cultured to 80%–90% confluence in a six-well plate, a line was gently drawn on the bottom of each well with a pipette and washed three times with PBS. The width of the scratch was measured after different treatments. The degree of the scratch healing was observed under an inverted microscope (BX50-FLA, Olympus, Tokyo, Japan).

### Matrigel tube formation assay

The 96-well plates were coated with 35 μl of growth factor-reduced Matrigel (354230, Biocoat, USA). After different treatments, HUVECs were seeded at 1 × 10^6^ cells/ml onto the Matrigel. After 5 h, the supernatant was discarded, and the cells were washed with PBS. Calcein-AM dye (5 μg/ml) was added and stained for 5 min. Photographs were taken using an inverted fluorescence microscope (SP8, Leica Microsystems CMS GmbH, Germany).

### Bone marrow-derived macrophage extraction

The 10-day-old SD rats were sterilized in beakers containing 75% ethanol for 3–5 min. The tibia and femur of the rats were stripped, and the bone marrow was squeezed out with forceps into the centrifuge tube with 1 ml of MEM (10% FBS, 1% P/S, M-CSF 10 μg/ml). Fivefold the amount of erythrocyte lysis solution was added, and the tube was placed at 4°C for 5 min, then centrifuged at 1,000 rpm/min for 3 min. The supernatant was discarded, the cells were resuspended by complete MEM medium and inoculated in 10-cm^2^ culture dishes. After 1 h, the supernatant was collected and inoculated in the corresponding culture plates, and half of the liquid volume was renewed after 2 days.

The groupings were as follows: control group, model group, STDP group, LY29 group, and LY29 + STDP group. BMDMs in the model group were treated with LPS (Beijing Biodee Biotechnology, 10 μg/ml) for 24 h. BMDMs in the inhibitor group were treated with LY294002 (154447-36-6, Selleck, USA) at a dose of 10 μM for 24 h. STDPs were administered at a dose of 400 µg/ml.

### Analysis of nitrogen monoxide production

BMDMs were cultured to 80%–90% confluence in a 96-well plate. After different treatments, the cell supernatant was extracted and assayed according to the NO assay kit method (bn27106-500, Biorigin, China). After adding the standards/samples, Griess reagent I and Griess reagent II, the plate was mixed well and incubated at room temperature for 5 min. NO concentration was measured at 540 nm using a microplate reader.

### Flow cytometry analysis

BMDMs were enzymatically digested for 40 s and prepared into a single-cell suspension. After centrifugation, the supernatant was discarded, and the cells were washed with PBS and centrifuged again. Anti-CD206 antibody (sc58986 PE, Santa Cruz, CA, USA) was added, and the BMDMs were incubated on ice for 40 min. After being washed twice, BMDMs were suspended in 300 μl of PBS and then detected by flow cytometry (Canton II BD, USA).

### Statistical analysis

All data were expressed as mean ± SD. Statistical analysis was performed using GraphPad Prism 6. Statistical analysis was performed using one-way analysis of variance (ANOVA). Dunnett’s test was used for multiple comparisons between groups. Values of *p* < 0.05 were considered statistically significant.

## Results

### Shexiang Tongxin dropping pills attenuated cardiac dysfunction in coronary microvascular dysfunction rats induced by left anterior descending ligation

**FIGURE 1:**
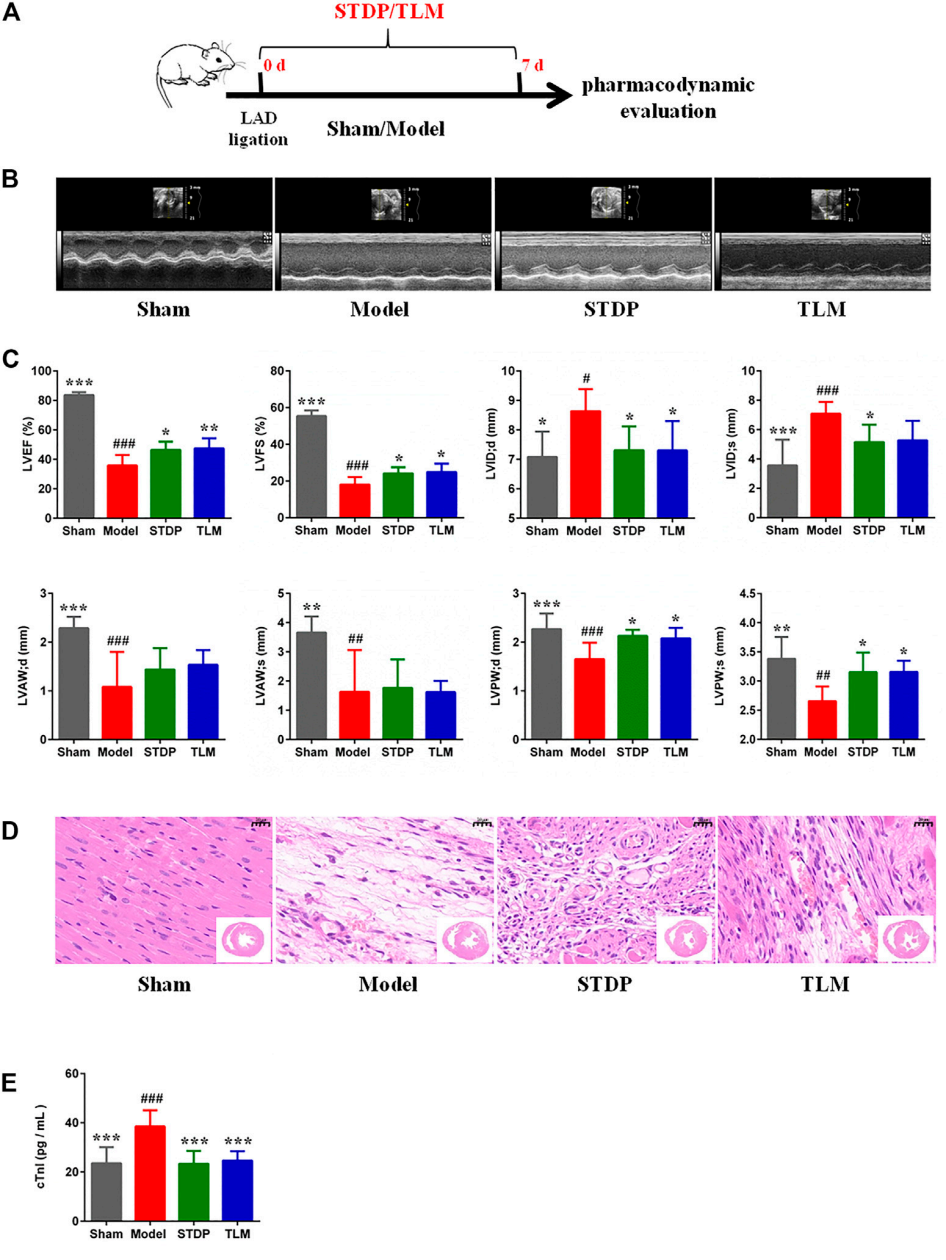
Effects of Shexiang Tongxin dropping pills (STDPs) on cardiac function. **(A)** The experimental protocol for STDP study in left anterior descending (LAD) ligation induced the coronary microvascular dysfunction (CMD) model rats. **(B)** Representative images of echocardiography in the sham, model, STDP, and TLM groups. **(C)** Echocardiographic parameter analysis in different groups. *N* = 6 per group. **(D)** Representative H and E staining of paraffin sections. Scale bar = 20 μm. **(E)** The serum troponin (cTnI) in different groups. *N* = 6 per group. *p < 0.05, **p < 0.01, ***p < 0.001 *vs*. the model group; ^#^ *p* < 0.05, ^# #^ *p* < 0.01, ^# # #^ *p* < 0.001 *vs*. the sham group.

To determine whether STDPs could attenuate cardiac dysfunction in CMD rats induced by LAD ligation, echocardiographic assessment, H and E staining, and ELISA assay were performed. The experimental protocol is presented in Figure 1A. Rats in the model group showed a significant reduction in cardiac function (Figures 1B,C). As shown by the echocardiography results, EF was decreased by 57.05%, and FS was decreased by 67.26% in the model group compared with the sham group, which were remarkably reversed by STDP administration. H&E staining of heart sections showed an obvious increase in vascular tube-like formation in the STDP group (Figure 1D). In addition, serum cTnI, an indicator of myocardial injury, was detected, and STDPs could diminish the release of cTnI into the serum compared with the model group (Figure 1E). Telmisartan was used as a positive drug. These results suggested that STDPs improved cardiac function in LAD ligation-induced CMD rats.

### Shexiang Tongxin dropping pills promoted angiogenesis by inducing the polarization of M2 macrophages in ischemic myocardial tissue

**FIGURE 2:**
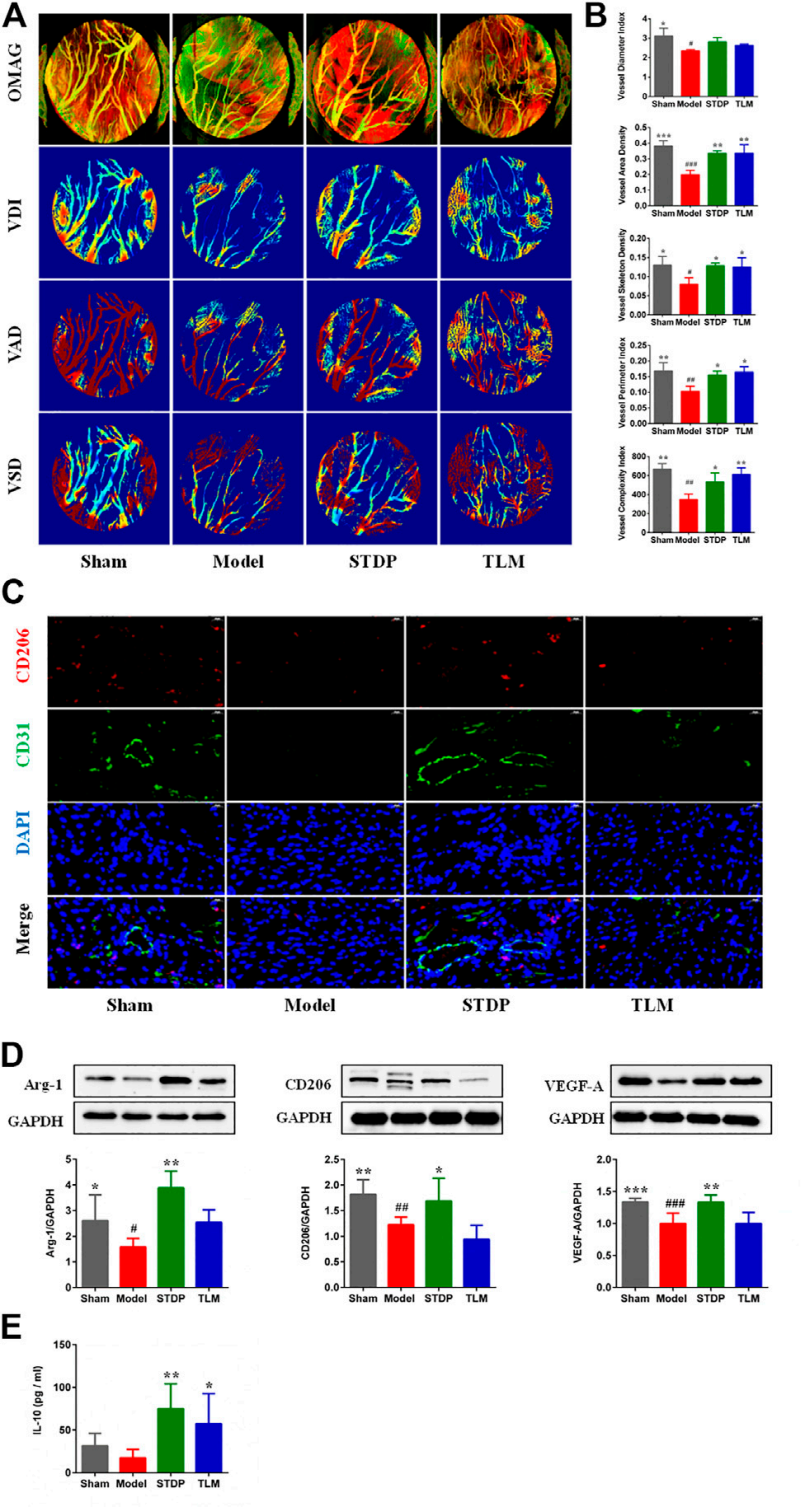
STDPs promoted angiogenesis by inducing polarization of M2 macrophages in ischemic myocardial tissues. **(A)** Representative real-time perfusion images of cardiac vessels by optical microangiography (OMAG). **(B)** Vessel diameter index (VDI), vessel area density (VAD), vessel skeleton density (VSD), vessel perimeter index, and vessel complexity index analysis in different groups. *N* = 3 per group. **(C)** IF staining of CD206 and CD31 in different groups. *N* = 3 per group. **(D)** Immunoblots and the quantification of Arg-1, CD206, and VEGF-A, *N* = 6 per group. **(E)** The level of IL-10 in serum. *N* = 6 per group. *p < 0.05, **p < 0.01 *vs*. the model group; ^#^ *p* < 0.05, ^# #^ *p* < 0.01 *vs.* the sham group.

The real-time perfusion of cardiac vessels in the different groups was assessed by OMAG, and the representative images are presented in Figure 2A. After STDP treatment, microvessel density increased significantly compared with the model group (Figure 2B). It is well known that M2 macrophages participate in the process of heart repair and angiogenesis (Hong and Tian, 2020), which are marked by CD206 and characterized by releasing high levels of Arg-1 and IL-10 (Wu et al., 2019). Therefore, the effects of STDPs on the polarization of M2 macrophage were explored. IF staining showed that compared with the model group, the number of M2 macrophages in the STDP group was upregulated **(** Supplementary Figure S2 **)**. Meanwhile, STDPs increased the expression of Arg-1 and CD206 in ischemic myocardial tissue compared with the model group (Figure 2D). STDP also could stimulate the release of IL-10 into the serum (Figure 2E). Interestingly, we found that increased M2 macrophages were around endothelial cells (Figure 2C), accompanied with an increased VEGF-A expression in the STDP group compared with the model group (Figure 2D). These results indicated that STDPs could promote angiogenesis by inducing M2 macrophage polarization in ischemic myocardial tissues.

### Shexiang Tongxin dropping pills upregulated the PI3K/Akt/mTORC1 pathway in the ischemic hearts of rats

**FIGURE 3:**
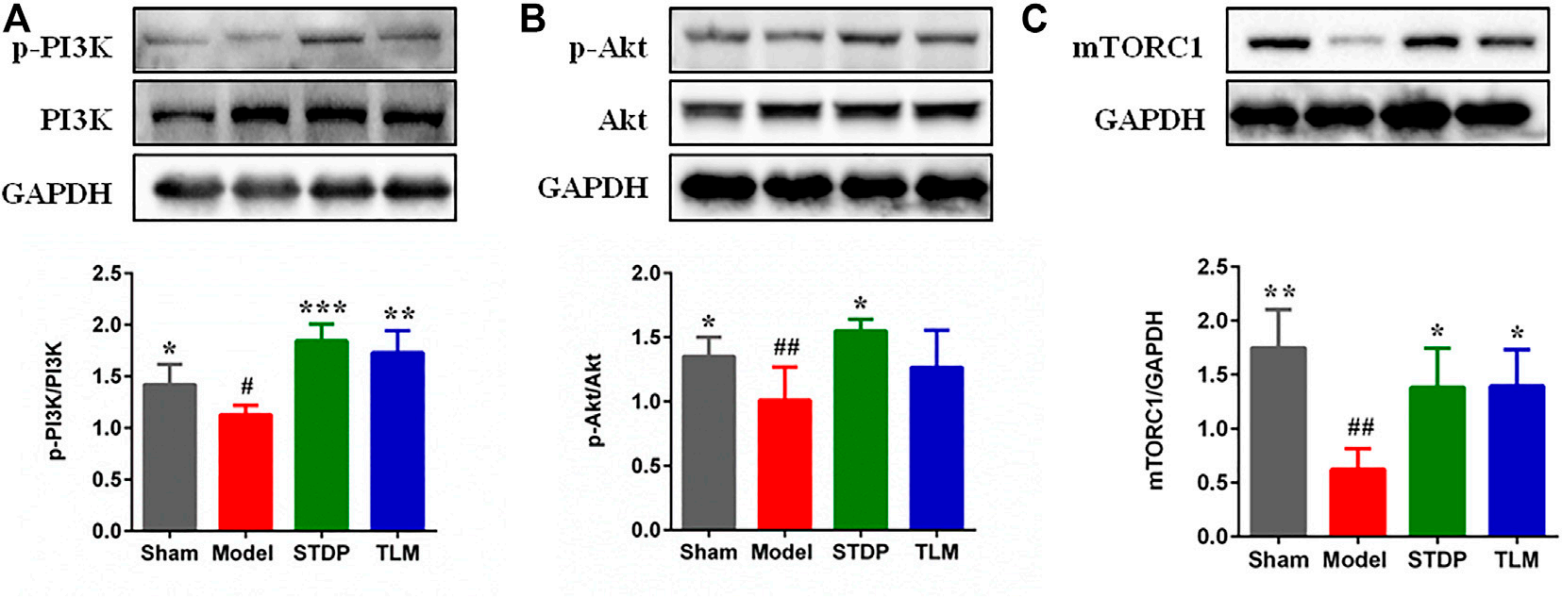
STDPs upregulated the PI3K/Akt/mTORC1 pathway in ischemic heart of rats. **(A–C)** Representative immunoblots and analysis for p-PI3K, *p*-Akt, and mTOCR1 in the sham, model, STDP, and TLM groups. *N* = 3 per group. *p < 0.05, **p < 0.01, ***p < 0.001 *vs*. the model group; ^#^ *p* < 0.05, ^# #^ *p* < 0.01 *vs*. the sham group.

Phosphorylation-activated PI3K/Akt was critically involved in macrophage polarization in heart tissues (Vergadi et al., 2017); thus, the effects of STDP on the expressions of p-PI3K and *p*-Akt were examined. Results showed that the expressions of p-PI3K and *p*-Akt were significantly decreased in the model group compared with the sham group. After treatment with STDPs, levels of p-PI3K and *p*-Akt were upregulated **(** Figures 3A,B **)**. Meanwhile, treatment of STDP could reverse the low expression of mTORC1 in the model group (Figure 3C). These data indicated that STDP could induce M2 macrophage polarization *via* regulating the PI3K/Akt/mTORC1 pathway.

### Shexiang Tongxin dropping pills enhanced the proliferation, migration, and tube formation of HUVECs

**FIGURE 4:**
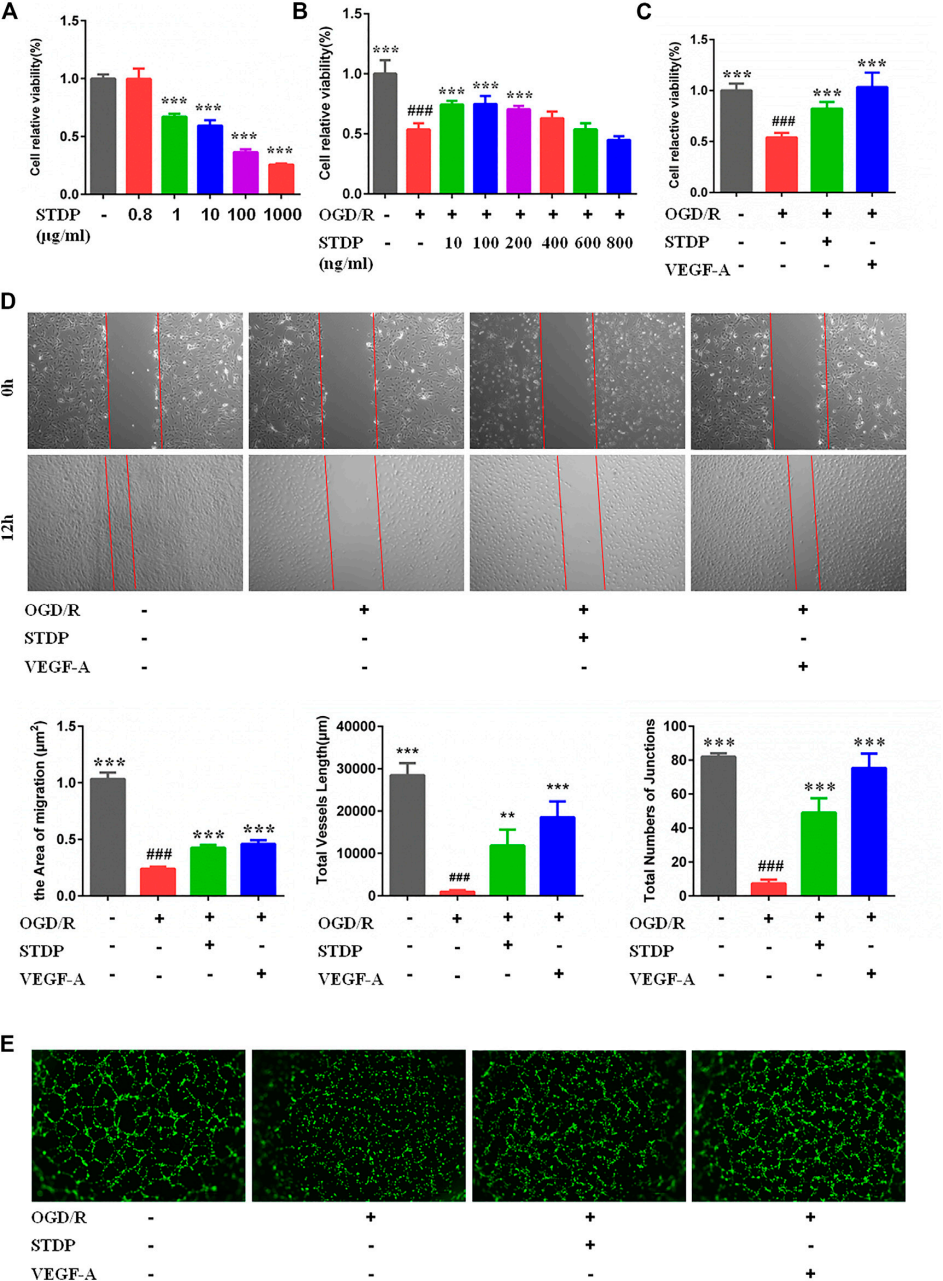
STDPs enhanced the proliferation, migration, and tube-forming capacity of HUVECs. **(A)** STDPs (800 ng/ml) have no cytotoxicity on HUVECs. **(B)** Bone marrow-derived macrophages (BMDMs) were treated with various concentrations of STDPs for 24 h **(C)** STDPs (100 ng/ml) promoted the cell viability in OGD/R-induced HUVECs. VEGF-A (20 ng/ml) promoted the proliferation capacity of HUVECs. *N* = 6 per group. **(D)** STDPs promoted cell migration in OGD/R-induced HUVECs. *N *= 3 per group. **(E)** STDPs promoted tube formation in OGD/R-induced HUVECs. *N* = 3 per group. VEGF-A was used as the positive control. **p < 0.01, ***p < 0.001 *vs*. the model group; ^# # #^ *p* < 0.001 *vs*. the sham group.

In order to explore the effect of STDPs on angiogenic properties of endothelial cells *in vitro*, we established the ODG/R-induced HUVECs model. The treatment of HUVECs with STDPs (10–200 ng/ml) for 24 h significantly enhanced cell viability compared with the model group **(** Figure 4A **)**. Among them, 100 μg/ml of STDPs was the best concentration for subsequent experiments to evaluate the effects and mechanism (Figure 4B **)**. The proliferation and migration of HUVECs in the model group were reduced, which were upregulated after STDP treatment (Figures 4C,D). The effect of STDPs on the angiogenetic properties of endothelial cells was further examined using the tube formation assay. The tube length and numbers of junctions treated with STDPs were significantly increased compared with the model group, and VEGF-A was used the positive control **(** Figure 4E **)**. These data suggested that STDPs could promote the angiogenesis of HUVECs directly.

### Shexiang Tongxin dropping pills modulated M2 macrophage polarization and increased VEGF-A release *via* the PI3K/AKT/mTORC1 pathway in LPS-induced bone marrow-derived macrophages

**FIGURE 5:**
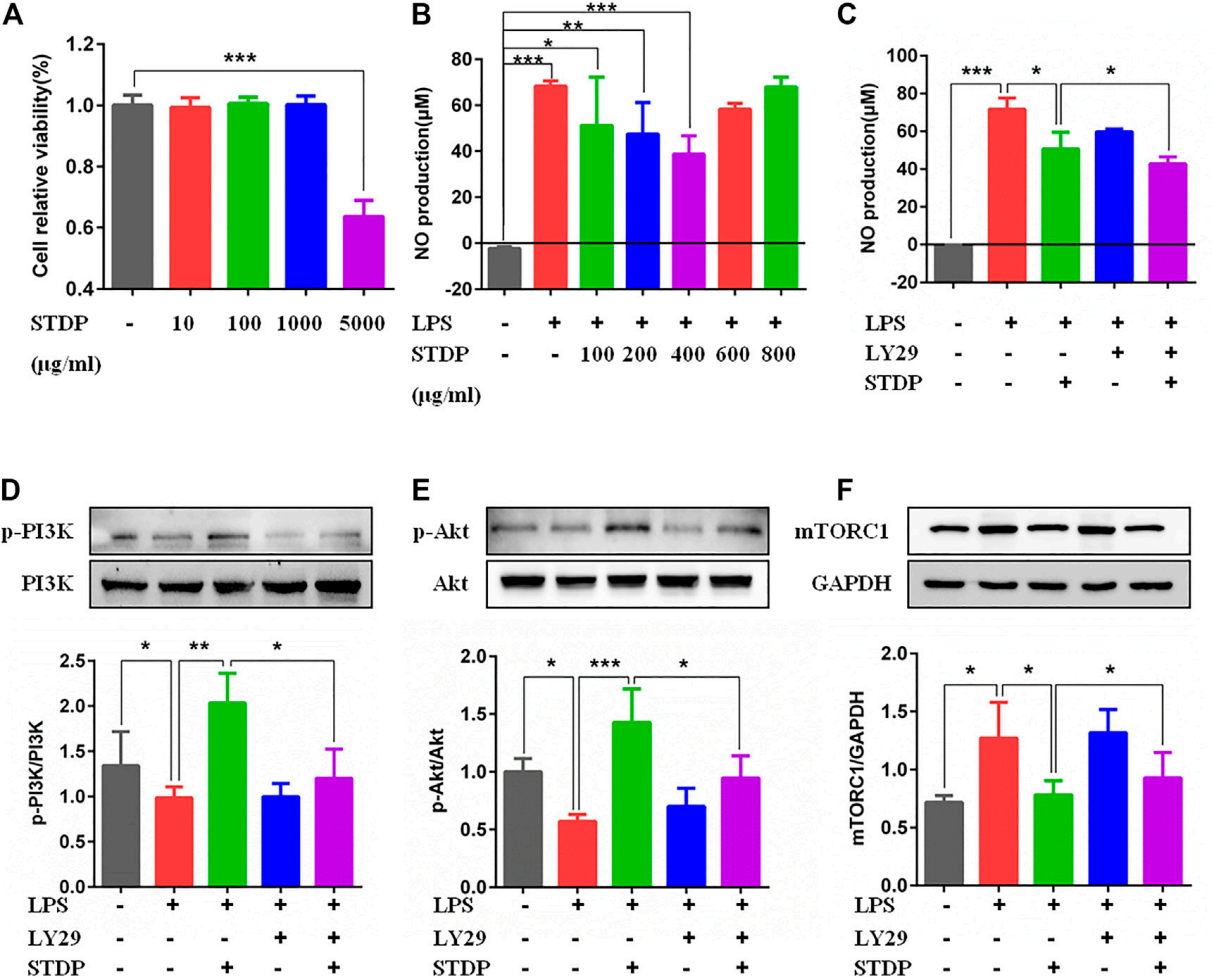
STDPs regulated the PI3K/AKT/mTORC1 pathway in BMDMs. **(A)** STDPs (10, 100, and 1,000 μg/ml) had no cytotoxicity on BMDMs. *N* = 6 per group. **(B, C)** NO level in the cell supernatant in the different groups. *N *= 6 per group. **(D–F)** Representative immunoblots and analysis for p-PI3K, *p*-Akt, and mTOCR1 in the control, model, STDP, LY29, and LY29+STDP groups. *N* = 3 per group. *p < 0.05, **p < 0.01, ***p < 0.001.

**FIGURE 6:**
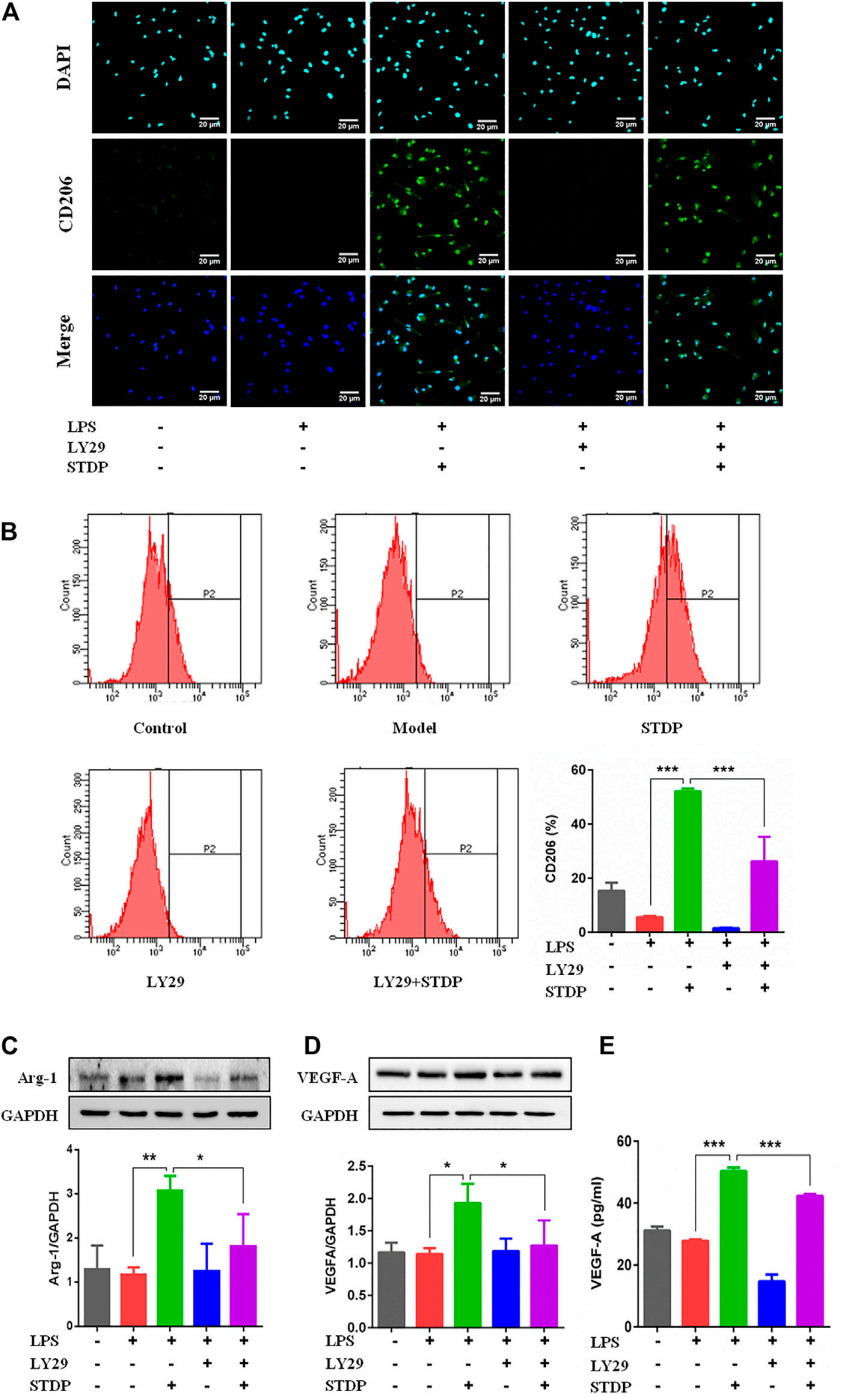
STDPs modulated M2 macrophage polarization and increased VEGF-A release. **(A)** IF staining of CD206 in the different groups. Scale bar = 20 μm. **(B)** Flow cytometry results showed that CD206-positive cells were upregulated after STDP treatment. *N* = 3 per group. **(C, D)** Representative immunoblots and analysis for Arg-1 and VEGF-A in different groups. *N* = 3 per group. **(E)** The release of VEGF-A in BMDM supernatant detected by ELISA. *N* = 3 per group. *p < 0.05, **p < 0.01, ***p < 0.001.

To further clarify the effects of STDPs on macrophage polarization-induced angiogenesis, LPS-induced BMDM inflammation model was used. CCK8 result showed that STDPs (10, 100, and 1,000 μg/ml) had no cytotoxicity on BMDMs **(** Figure 5A **)**. The effective concentrations of STDPs were assessed by the NO level in the cell supernatant. STDPs (400 μg/ml) had the best inhibitory effect on the release of NO after LPS stimulation (Figure 5B) and were used in the subsequent experiments. IF staining showed that STDPs obviously increased CD206 expression in LPS-induced BMDMs (Figure 6A). Similarly, CD206-postive BMDMs detected by flow cytometry were upregulated after STDP treatment, which were identical to the IF results (Figure 6B). The expression of Arg-1 in the STDP group was increased compared with the model group (Figure 6C). In addition, STDPs enhanced the expression of VEGF-A (Figure 6D) and the release of VEGF-A in the supernatant (Figure 6E). These results revealed that STDPs modulated M2 macrophage polarization and increased VEGF-A release, which played important roles in angiogenesis.

**FIGURE 7:**
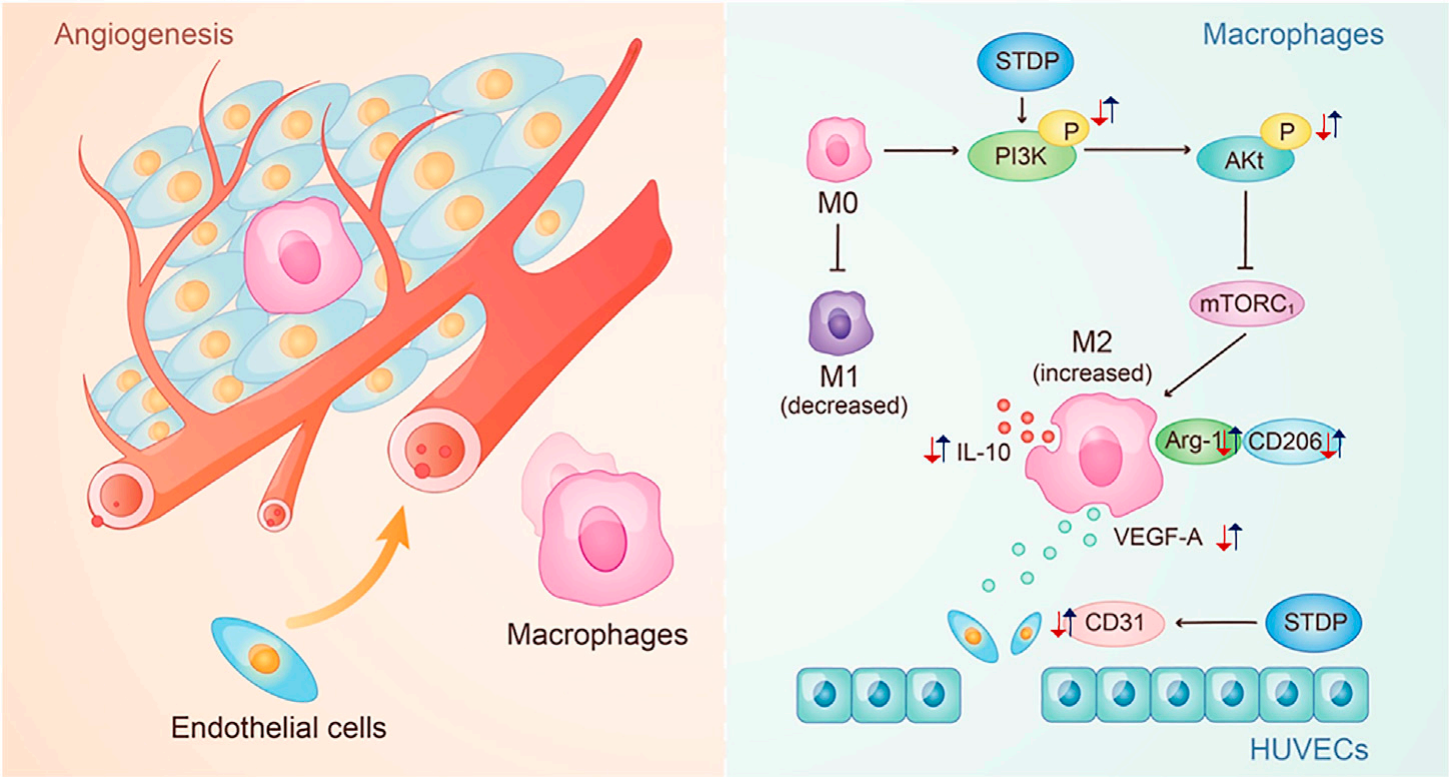
Potential mechanism of STDPs on CMD. The red arrows represent the change trend of key factors in the pathological environment, and the blue arrows represent the change trend of key factors after the STDPs administration.

Furthermore, the effective mechanism of STDPs on macrophage polarization-induced angiogenesis was assessed. Immunoblot results showed that STDPs could promote the expressions of p-PI3K and *p*-Akt in LPS-induced BMDMs compared with the model group (Figures 5D, E). In contrast, the expression of mTORC1 was increased in the model group, and STDP treatment inhibited mTORC1 expression (Figure 5F). Then LY294002, the inhibitor of PI3K, was applied. LY294002 reversed the effects of STDPs on NO release, and the expressions of p-PI3K, *p*-Akt, and mTORC1 (Figures 5C–F). At the same time, the enhancement of STDPs on M2 macrophage polarization and VEGF-A release could both be reversed by LY294002 (Figure 6E). Therefore, STDPs modulated M2 macrophage polarization and increased VEGF-A release *via* the PI3K/AKT/mTORC1 pathway in LPS-induced BMDMs **(** Figure 7 **)**.

## Discussion

In this study, we constructed the LAD ligation-induced CMD rat model, LPS-stimulated BMDM model, and OGD/R-induced HUVEC model to explore the effects and mechanism of STDPs on angiogenesis against CMD. We found that 1) STDPs ameliorated cardiac function in LAD ligation-induced CMD rats by promoting microvessel density, accompanied with increased infiltration of anti-inflammatory M2 macrophages in the ischemic heart. 2) STDPs modulated M2 macrophage polarization and increased VEGF-A release in LPS-stimulated BMDMs. 3) STDPs could regulate the proliferation, migration, and angiogenesis of HUVECs directly. 4) STDPs promoted macrophage polarization-induced angiogenesis *via* the PI3K/Akt/mTORC1 pathway both *in vivo* and *in vitro*.

CMD leads to a variety of cardiovascular diseases, and the main pathological mechanism of CMD is the imbalance of the vascular system due to insufficient collateral angiogenesis (Olivotto et al., 2011; Taqueti and Carli, 2018). Therapeutic angiogenesis is one of the effective treatments to restore microcirculation (Ahn et al., 2008). Previous studies showed that LAD ligation of rats could induce coronary microvascular dysfunction with a low microvascular density and impaired endothelial function (Zhang et al., 2014; Zhai et al., 2020); thus, the LAD ligation rat model was constructed to explore the effects of STDPs on improving CMD in our study. The results demonstrated that cardiac function was protected, and microvascular density was increased in CMD rats after STDP treatment. OMAG is a novel technology to exhibit the real-time perfusion of cardiac vessels and microvascular density (Qin et al., 2016). The visual results made the proangiogenic effect of STDPs more definite. Intriguingly, the number of M2 macrophages in myocardial tissue was enhanced at the same time, with simultaneous increase in VEGF-A and IL-10 levels. The more direct evidence was that the increased expression of CD31 was accompanied by an increase in CD206 in the periphery, suggesting that STDPs promoted M2 macrophage-dependent angiogenesis. We further investigated the potential mechanism of STDP-promoted angiogenesis *in vitro* experiments. In LPS-stimulated BMDMs, STDPs could increase the number of M2 macrophages, and promote Arg-1 expression and VEGF-A secretion. Consistent with a previous study, increased VEGF-A promoted angiogenesis in OGD/R-induced HUVECs. In addition to affecting macrophage-induced angiogenesis, we found that STDPs also could directly regulate the proliferation, migration, and tube formation of HUVECs. It may be related to the multicomponent and multitarget characteristics of traditional Chinese medicine.

It was reported that Ginsenoside Rg3 attenuated LPS-induced acute lung injury by inhibiting PI3K/Akt activation and macrophage polarization (Yang et al., 2018). Phosphorylated PI3K activated Akt activity, and then activated or inhibited the activity of downstream proteins through phosphorylation, which affected the levels of M2 macrophage markers (Lu et al., 2017). Vergadi et al. found that multiple factors, such as IL-10 and TGF-β, induced M2 macrophage polarization, which were counteracted by inhibition of Akt activation (Vergadi et al., 2017). mTORC1 is a downstream protein of the PI3K/Akt pathway. Akt promoted mTORC1 activation, which mediated feedback inhibition to suppress Akt activity (Covarrubias et al., 2015). Our results identified that STDPs promoted macrophage polarization-induced angiogenesis via the PI3K/Akt/mTORC1 pathway, which was reversed by the PI3K inhibitor LY294002.

However, there are still some defects in this study. For example, in this study, STDPs consisted of seven herbal APIs, not a monomeric mixture, so the dose selection and determination did not exactly refer to the dosing criteria for consensus document and may have some limitations. The active monomer components of STDPs in improving CMD are still unknown, and they need to be explored further. In addition, more studies are needed to elucidate the potential mechanisms by which PI3K/AKT/mTORC1 and its downstream effectors regulate M1/M2 macrophage polarization following CMD.

## Conclusion

In summary, this study provided conclusive evidence that STDPs promoted macrophage polarization-induced angiogenesis against CMD *via* the PI3K/Akt/mTORC1 pathway. Our results demonstrated that the phenotype transformation of macrophages and stimulation of the secretion of VEGF-A may be applied as novel cardioprotective targets for the treatment of CMD.

## Data Availability

The original contributions presented in the study are included in the article/Supplementary Material. Further inquiries can be directed to the corresponding authors.
