# Supplementary figures and images for: Shexiang Tongxin Dropping Pills Promote Macrophage Polarization-Induced Angiogenesis Against Coronary Microvascular Dysfunction via PI3K/Akt/mTORC1 Pathway (part 1 of 2)
Source: Front Pharmacol. 2022 Mar 23;13:840521. doi: 10.3389/fphar.2022.840521 (PMC8984141; doi:10.3389/fphar.2022.840521)

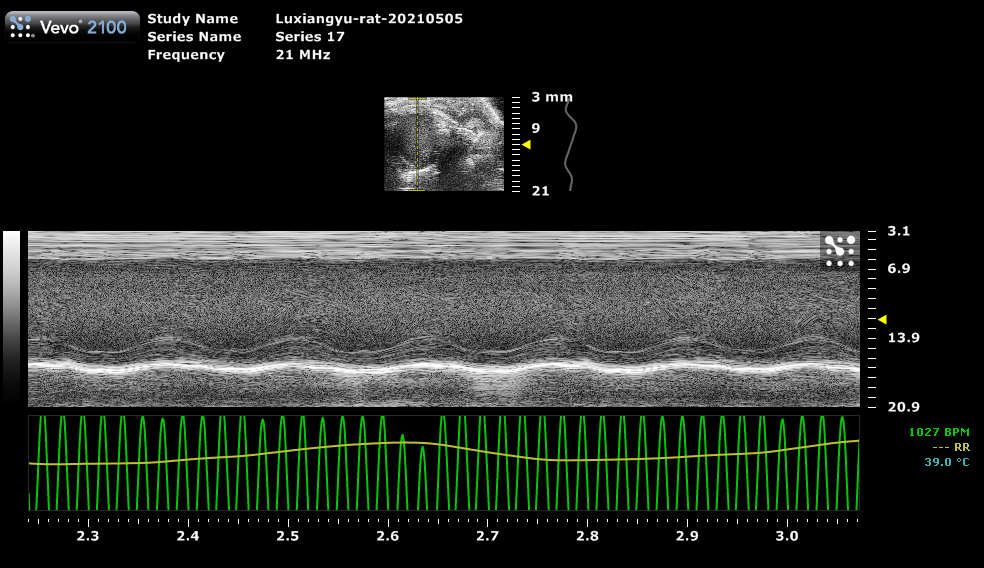

Supplement: Supplementary file 1 [file DataSheet3.ZIP › Raw data-2/Echocardiographic/Model.tif]

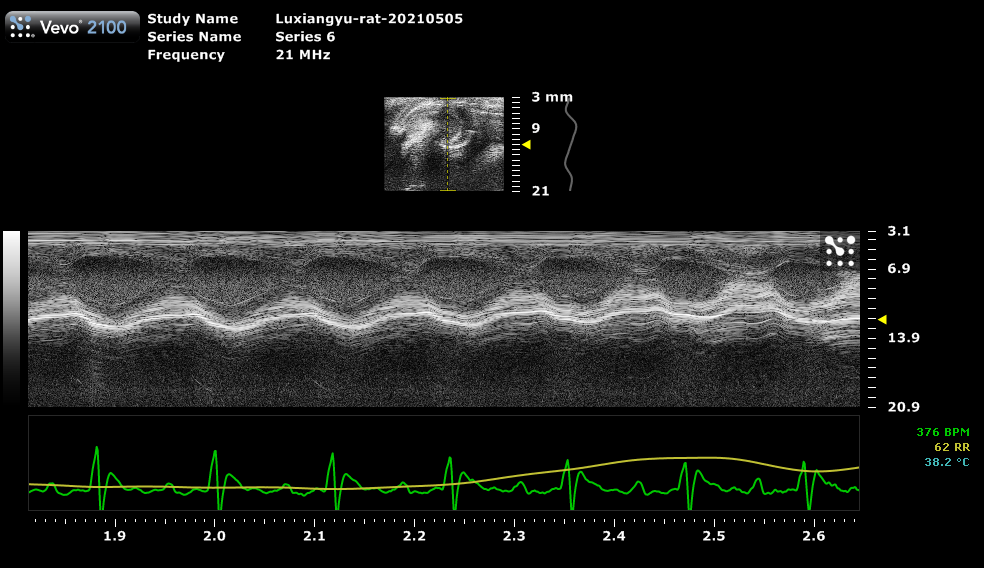

Supplement: Supplementary file 1 [file DataSheet3.ZIP › Raw data-2/Echocardiographic/Sham.tif]

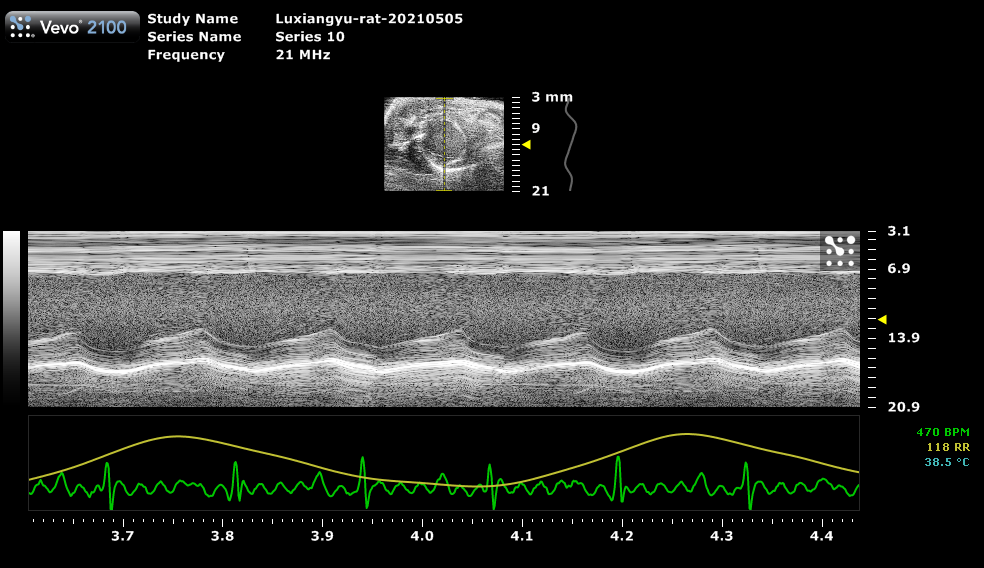

Supplement: Supplementary file 1 [file DataSheet3.ZIP › Raw data-2/Echocardiographic/STDP.tif]

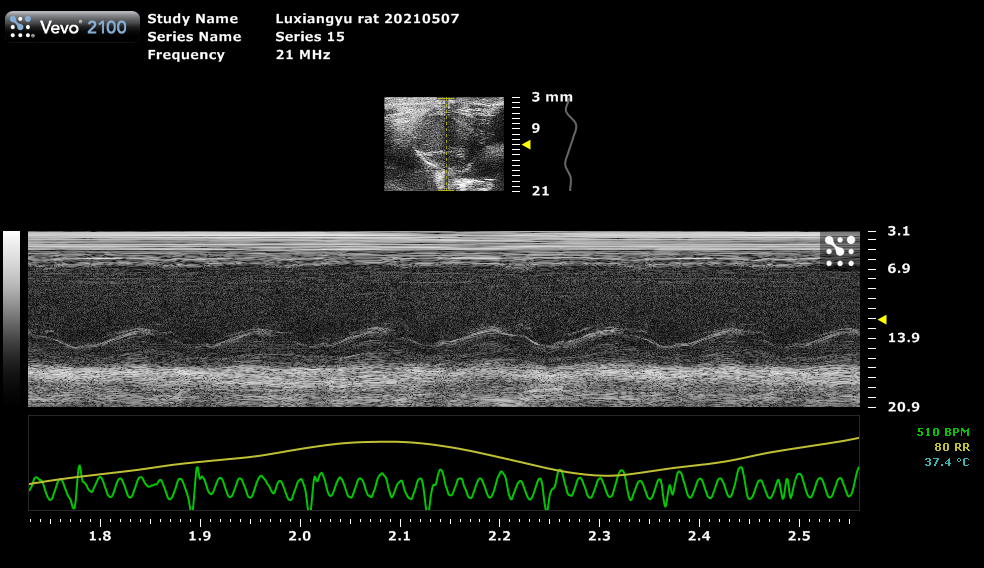

Supplement: Supplementary file 1 [file DataSheet3.ZIP › Raw data-2/Echocardiographic/TLM.tif]

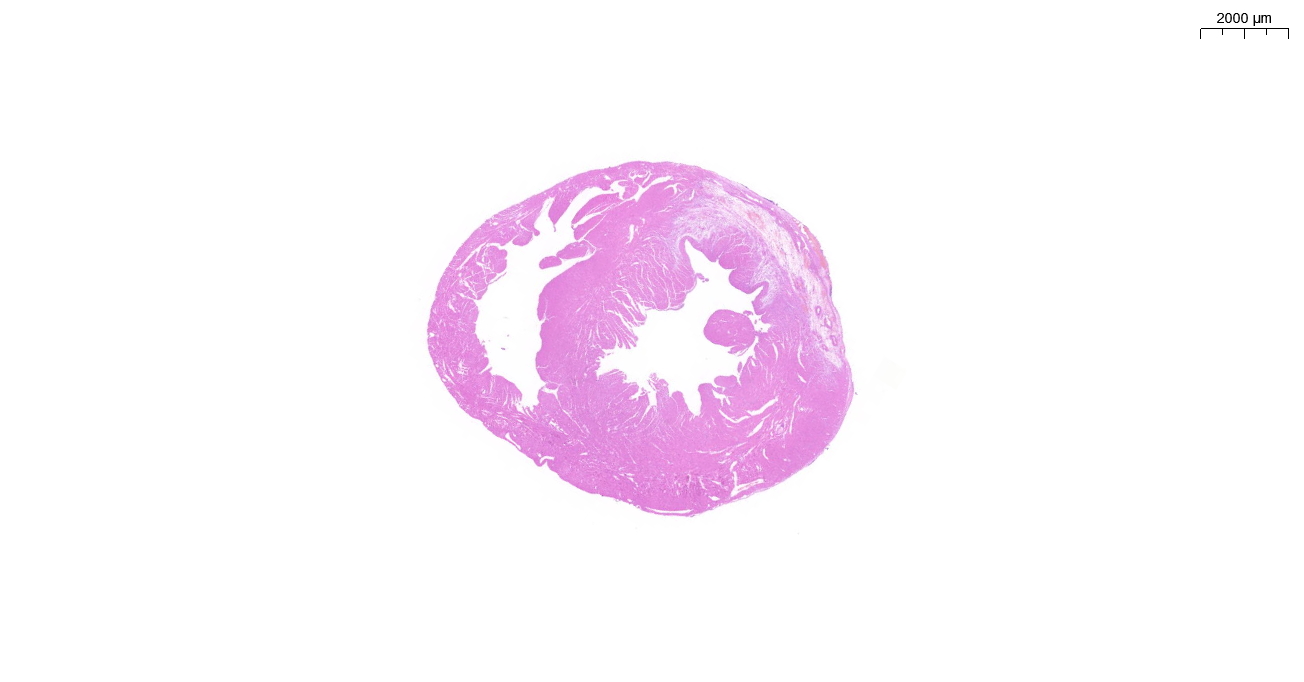

Supplement: Supplementary file 1 [file DataSheet3.ZIP › Raw data-2/Histological examination/Model-0.4x.jpg]

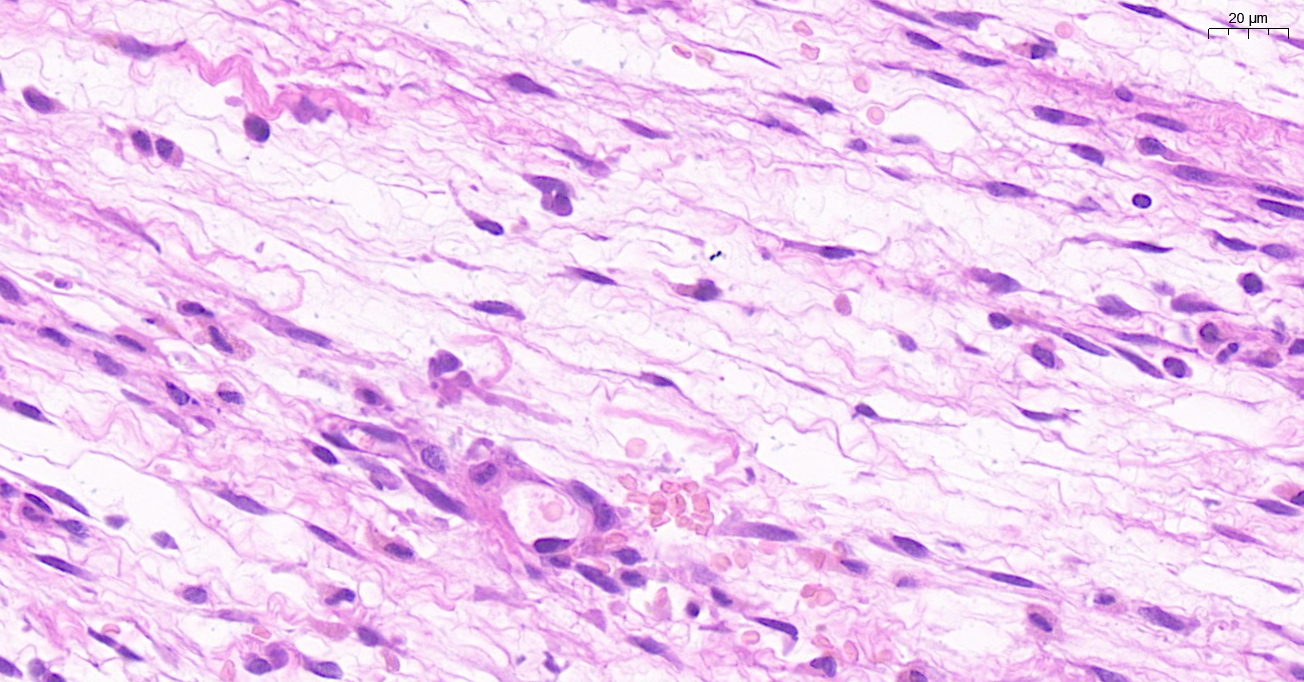

Supplement: Supplementary file 1 [file DataSheet3.ZIP › Raw data-2/Histological examination/Model-40.0x.jpg]

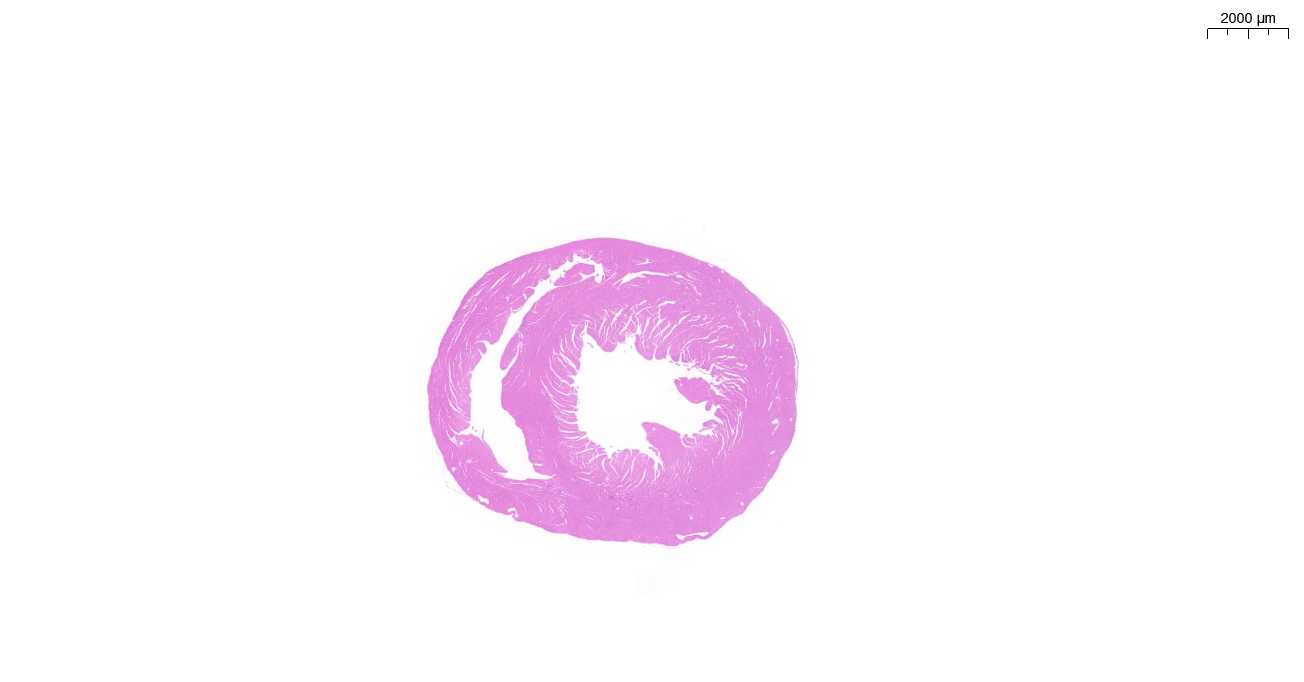

Supplement: Supplementary file 1 [file DataSheet3.ZIP › Raw data-2/Histological examination/Sham-0.4x.jpg]

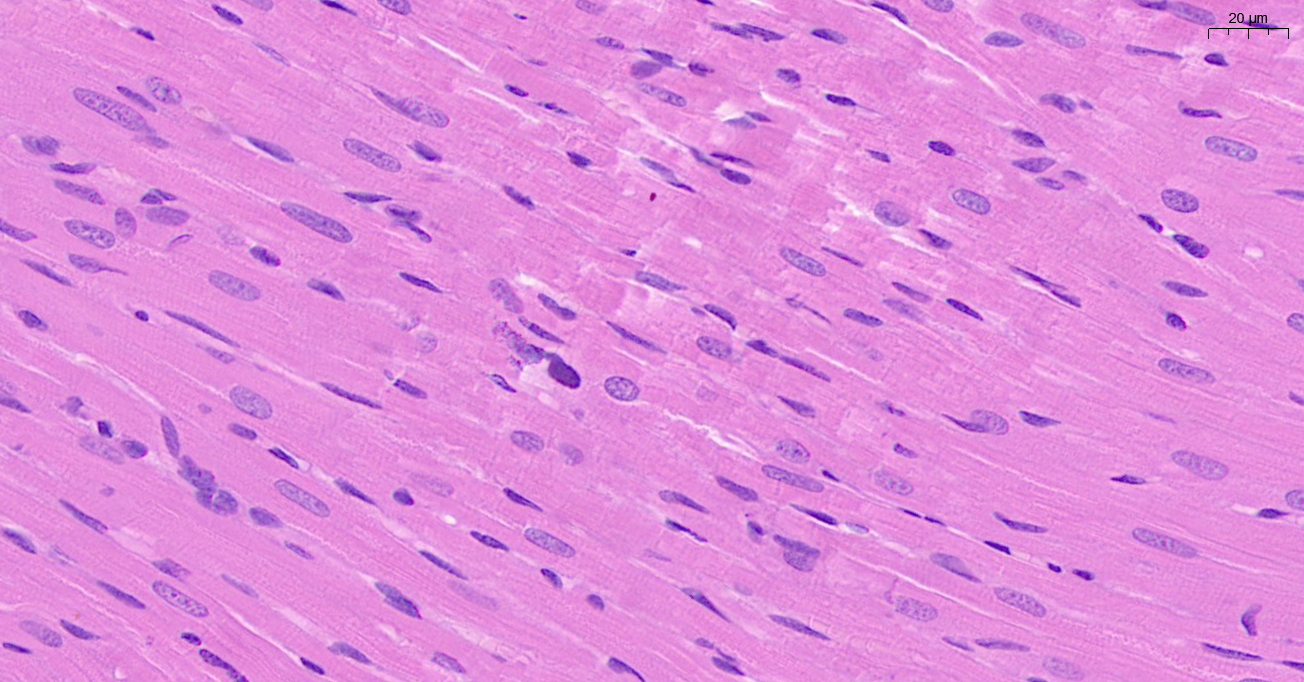

Supplement: Supplementary file 1 [file DataSheet3.ZIP › Raw data-2/Histological examination/Sham-40.0x.jpg]

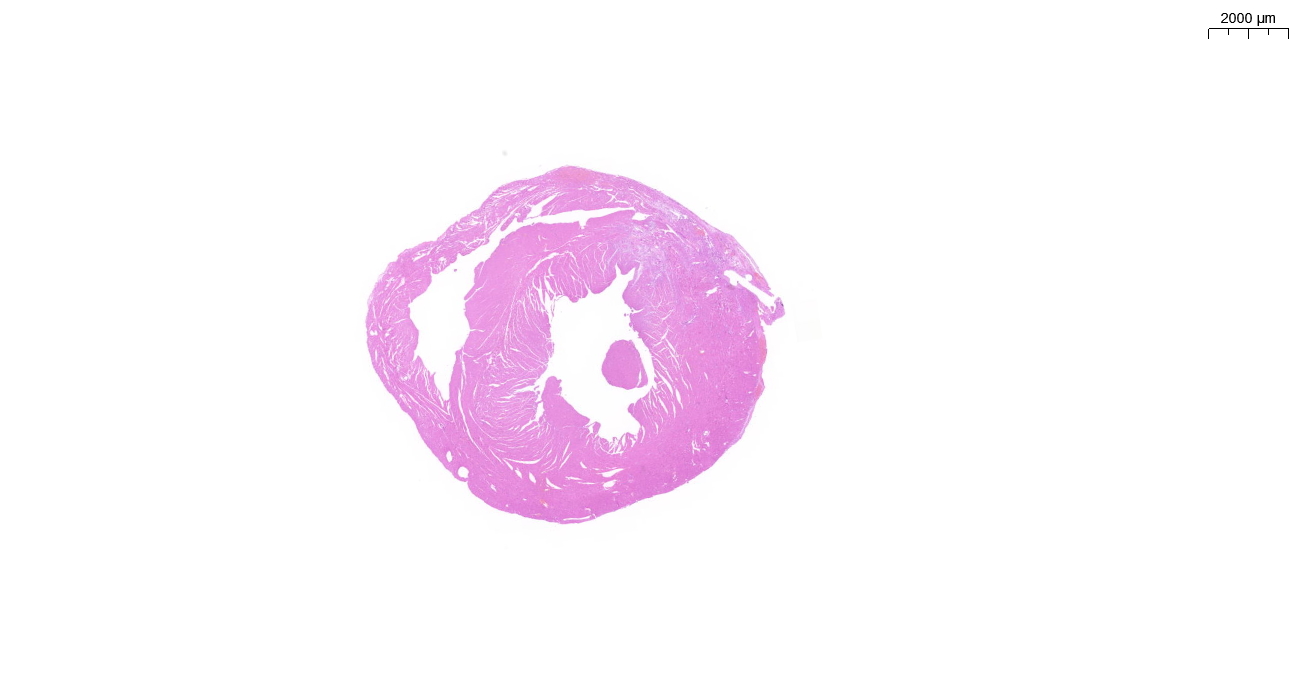

Supplement: Supplementary file 1 [file DataSheet3.ZIP › Raw data-2/Histological examination/STDP-0.4x.jpg]

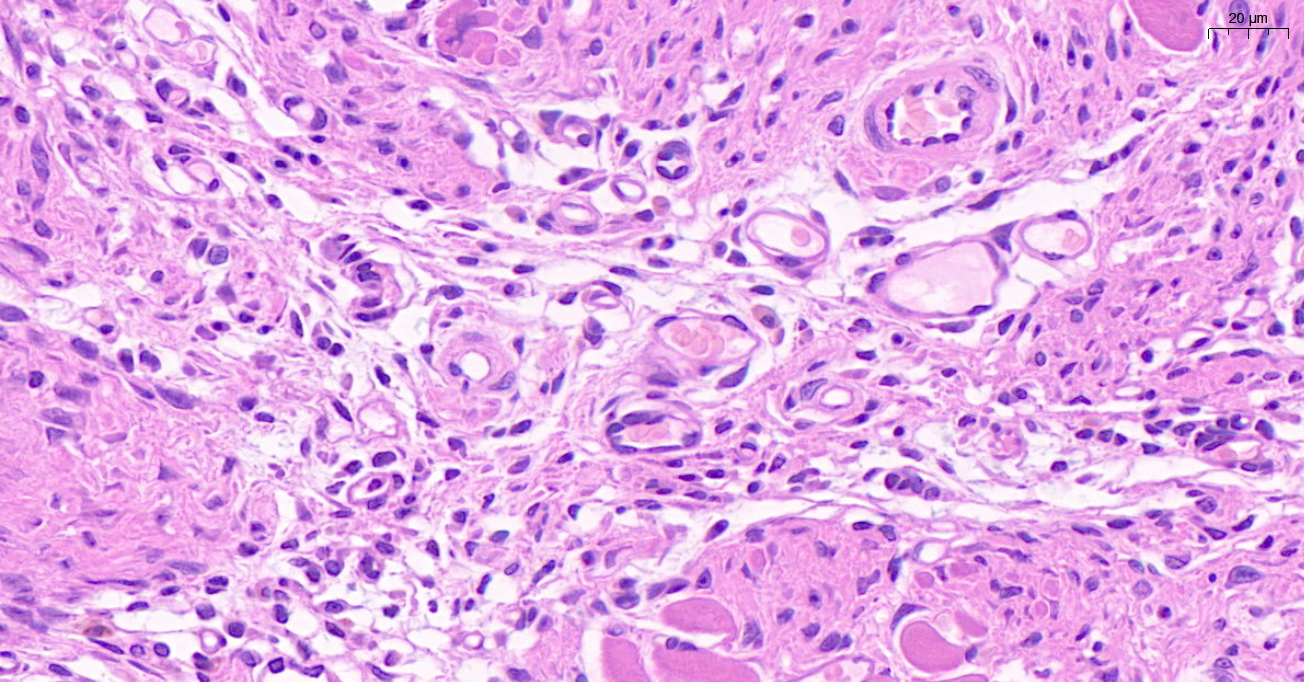

Supplement: Supplementary file 1 [file DataSheet3.ZIP › Raw data-2/Histological examination/STDP-40.0x.jpg]

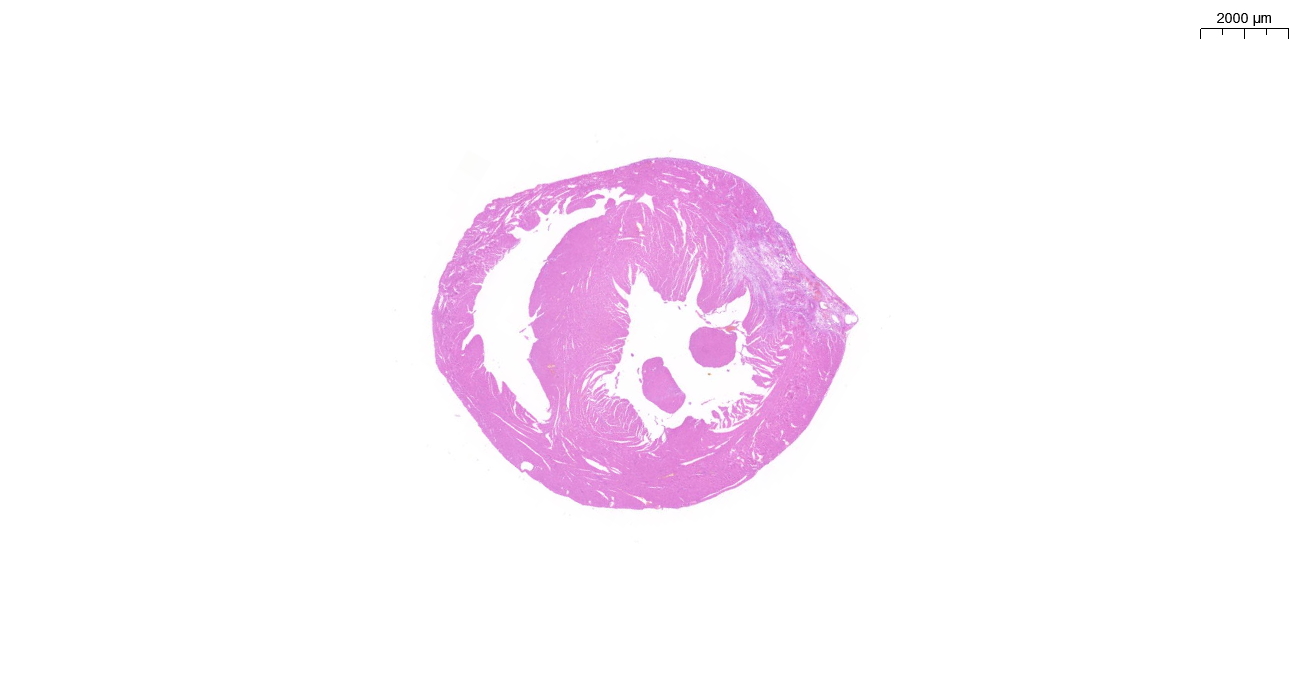

Supplement: Supplementary file 1 [file DataSheet3.ZIP › Raw data-2/Histological examination/TLM-0.4x.jpg]

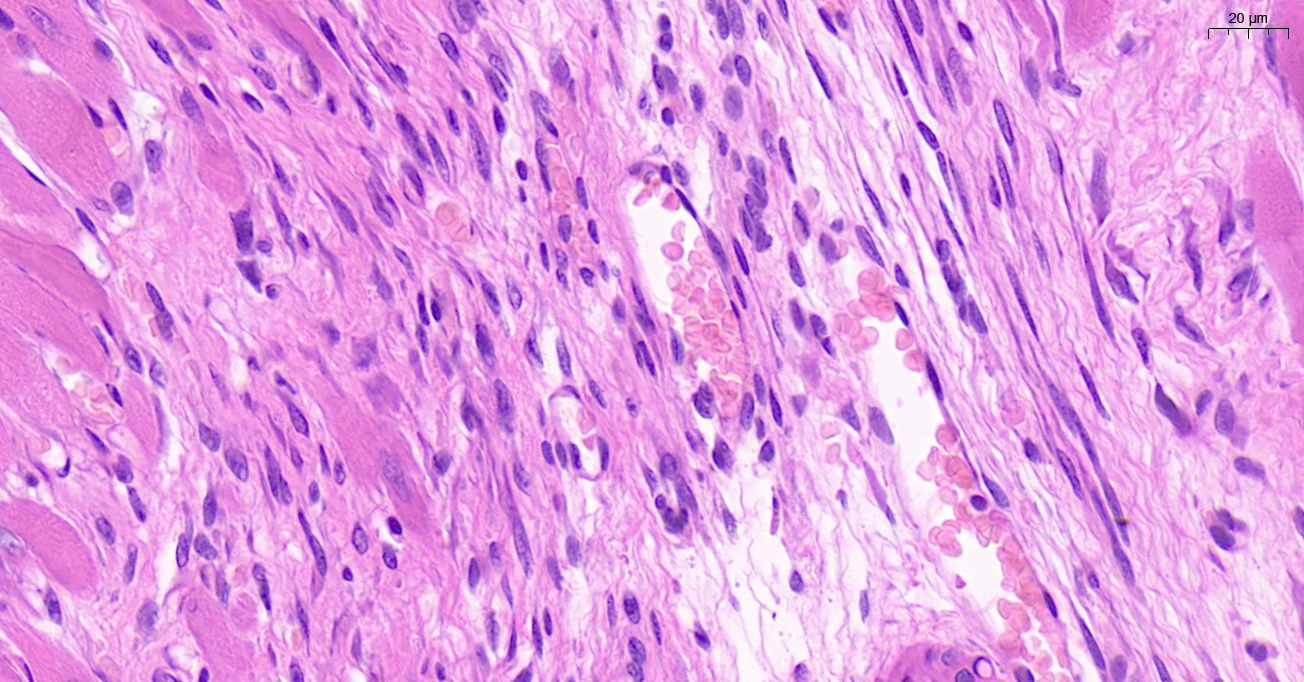

Supplement: Supplementary file 1 [file DataSheet3.ZIP › Raw data-2/Histological examination/TLM-40.0x.jpg]

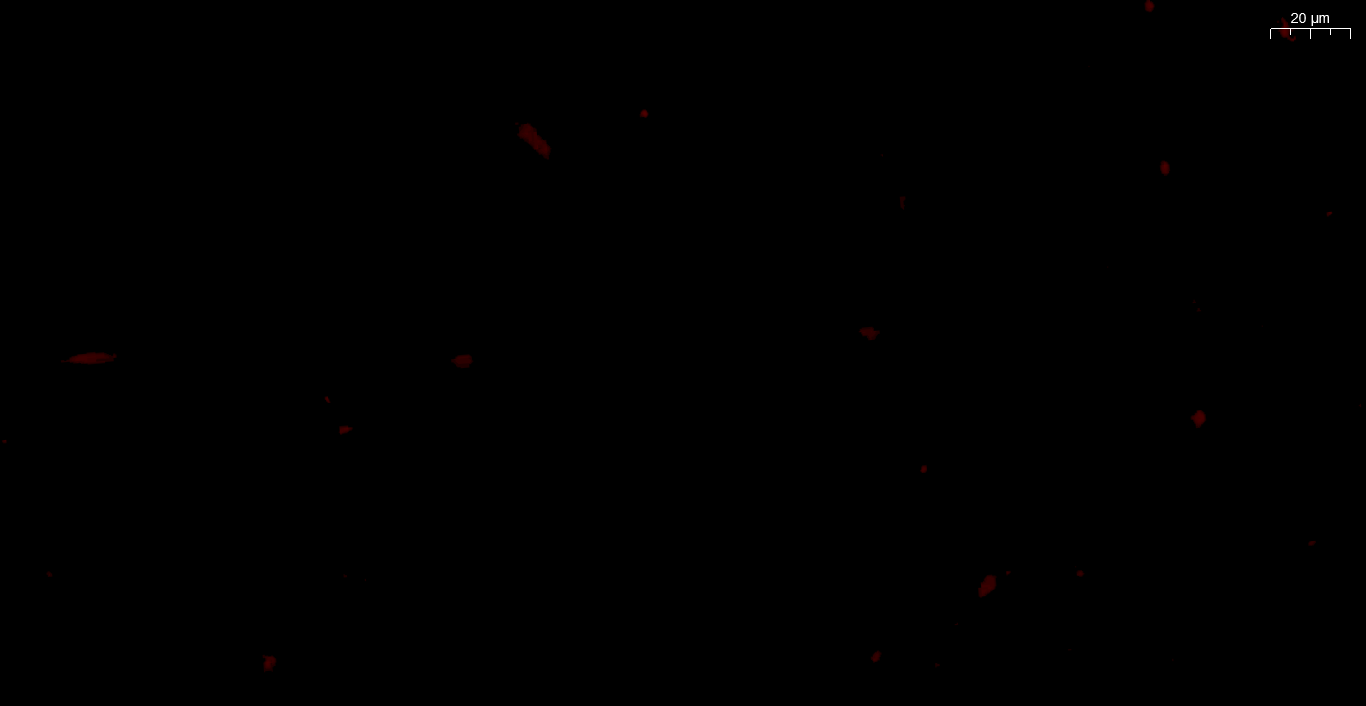

Supplement: Supplementary file 1 [file DataSheet3.ZIP › Raw data-2/Immunofluorescence (IF) Analysis/in vivo/Model/Model-CD206.tif]

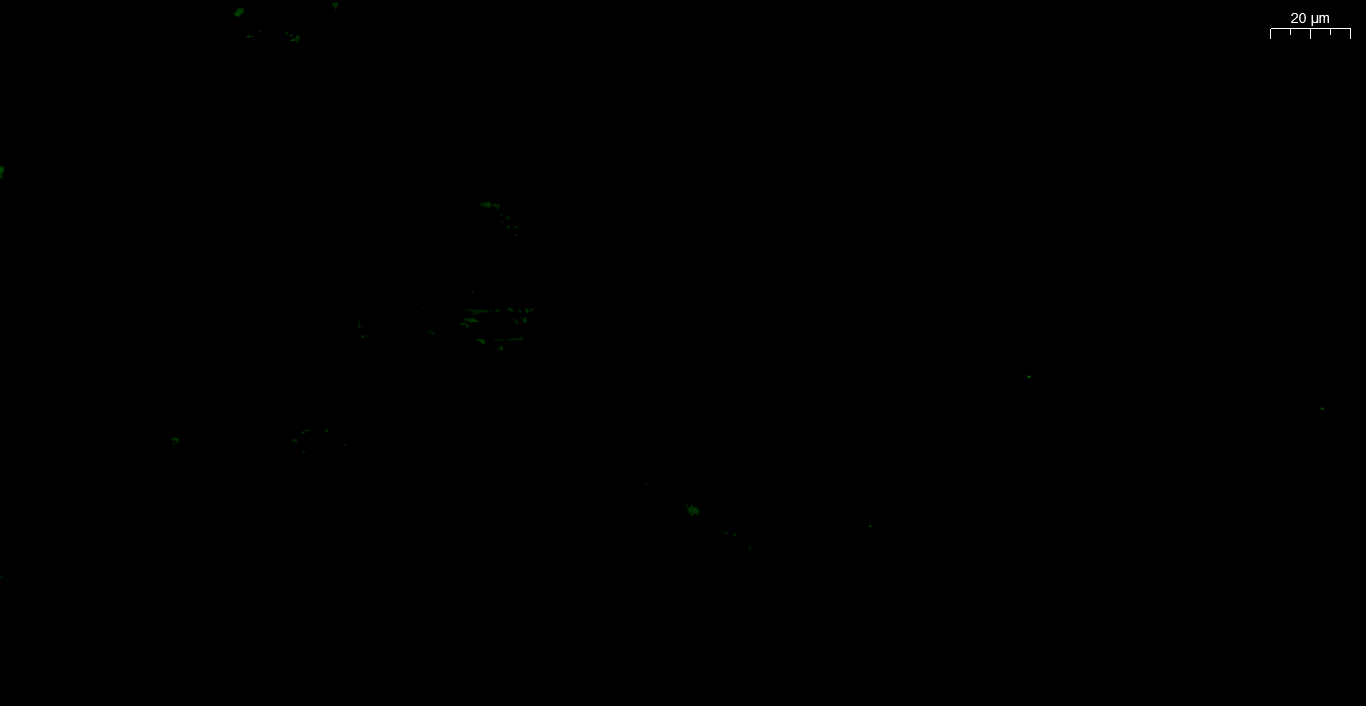

Supplement: Supplementary file 1 [file DataSheet3.ZIP › Raw data-2/Immunofluorescence (IF) Analysis/in vivo/Model/Model-CD31.tif]

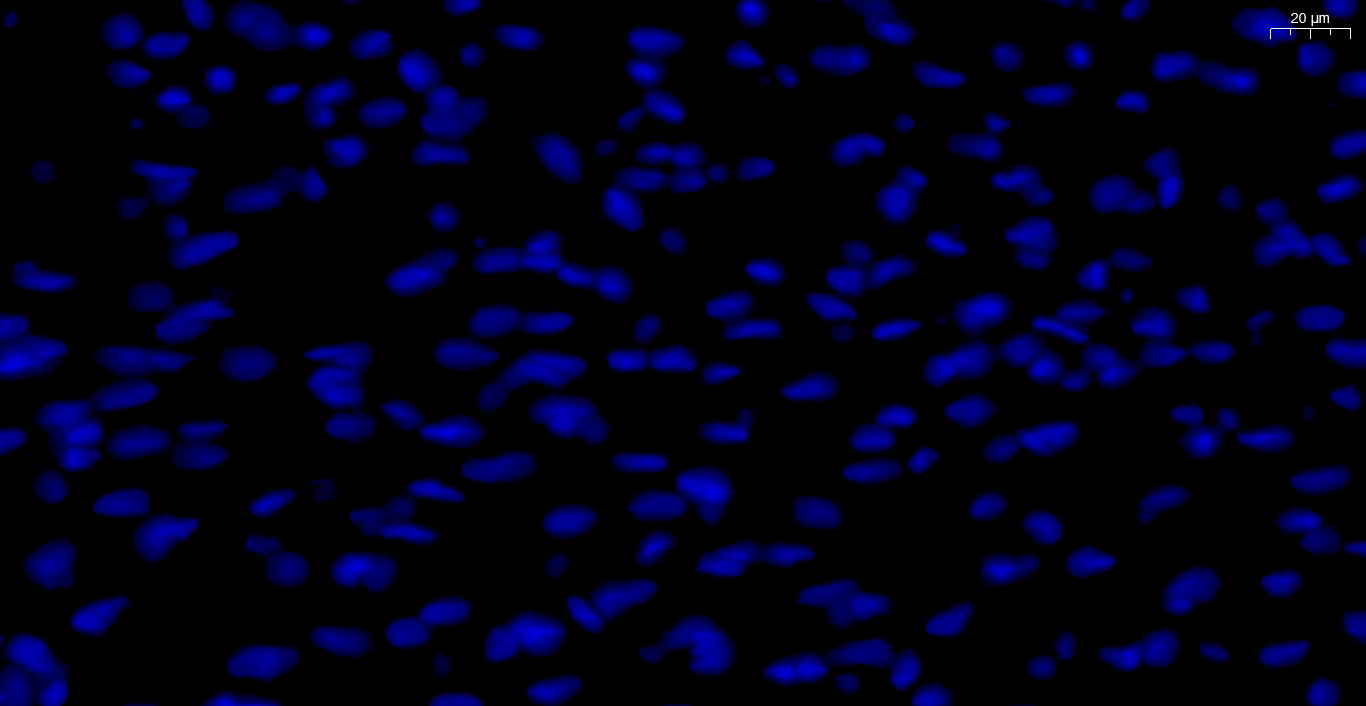

Supplement: Supplementary file 1 [file DataSheet3.ZIP › Raw data-2/Immunofluorescence (IF) Analysis/in vivo/Model/Model-DAPI.tif]

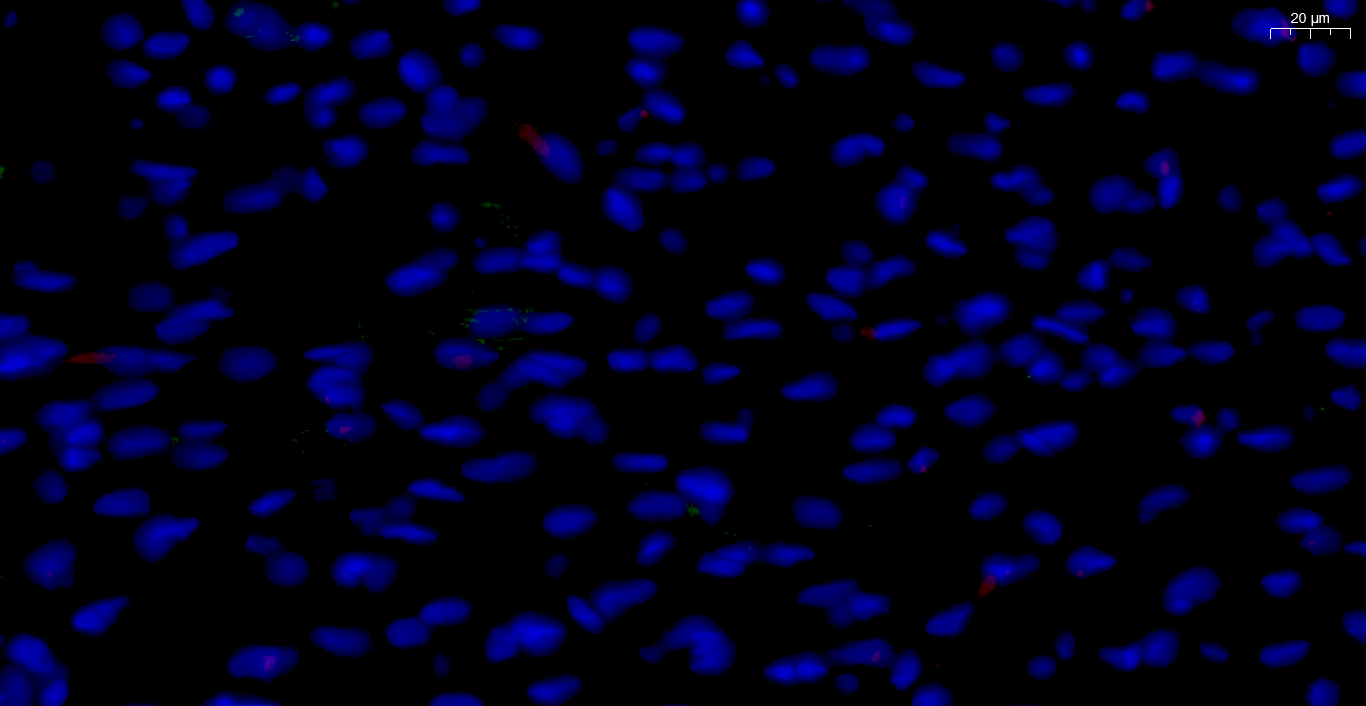

Supplement: Supplementary file 1 [file DataSheet3.ZIP › Raw data-2/Immunofluorescence (IF) Analysis/in vivo/Model/Model-MERGE.tif]

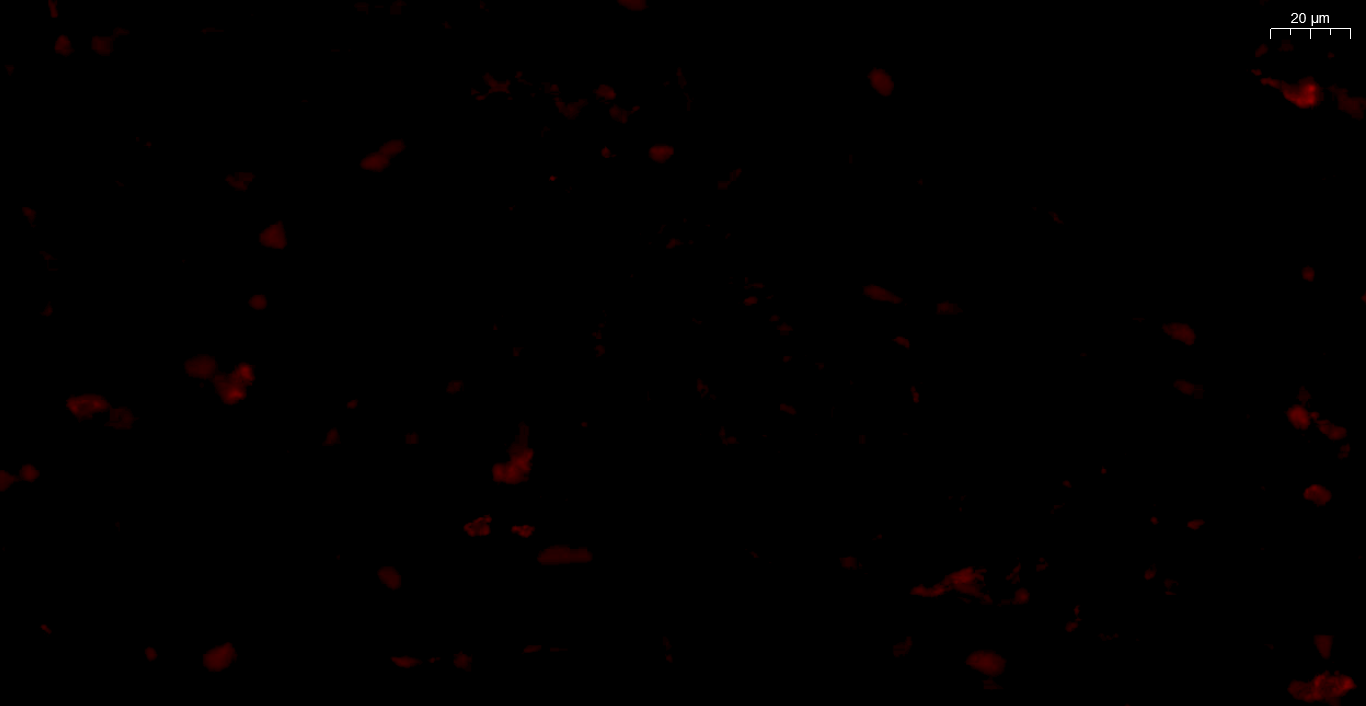

Supplement: Supplementary file 1 [file DataSheet3.ZIP › Raw data-2/Immunofluorescence (IF) Analysis/in vivo/Sham/Sham-CD206.tif]

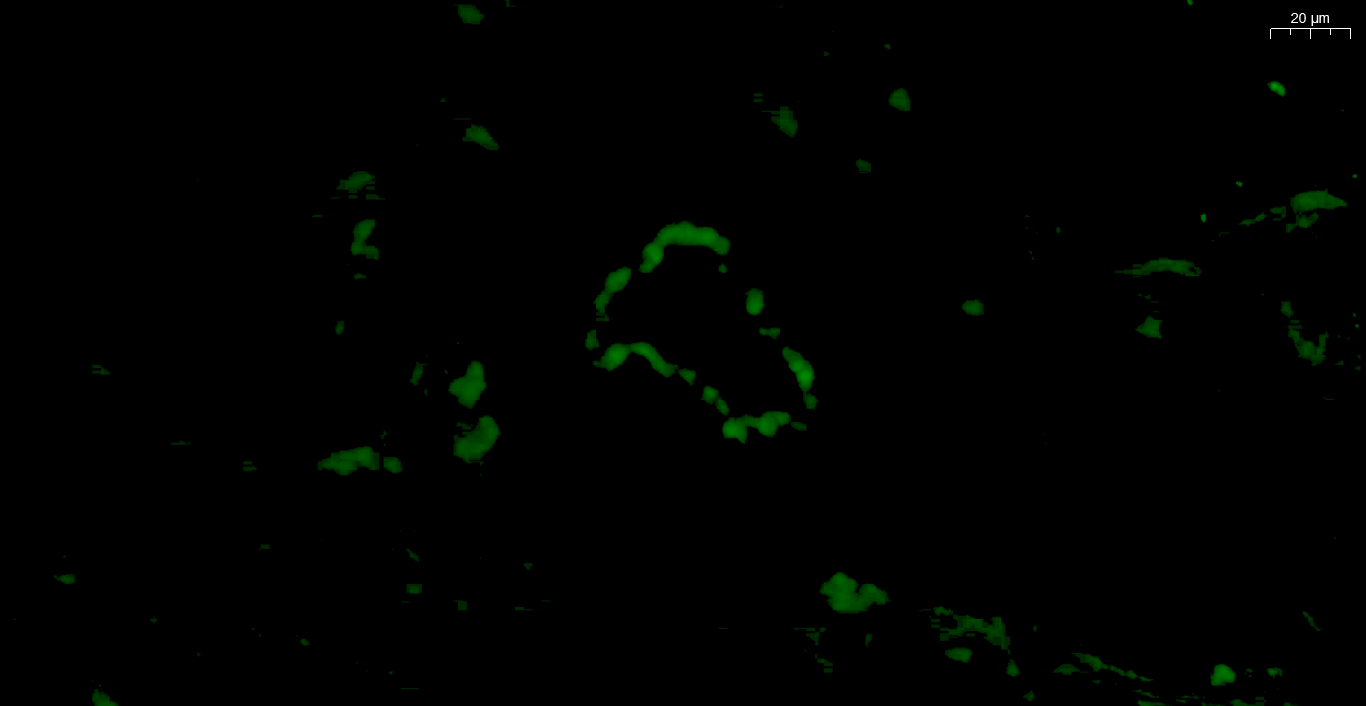

Supplement: Supplementary file 1 [file DataSheet3.ZIP › Raw data-2/Immunofluorescence (IF) Analysis/in vivo/Sham/Sham-CD31.tif]

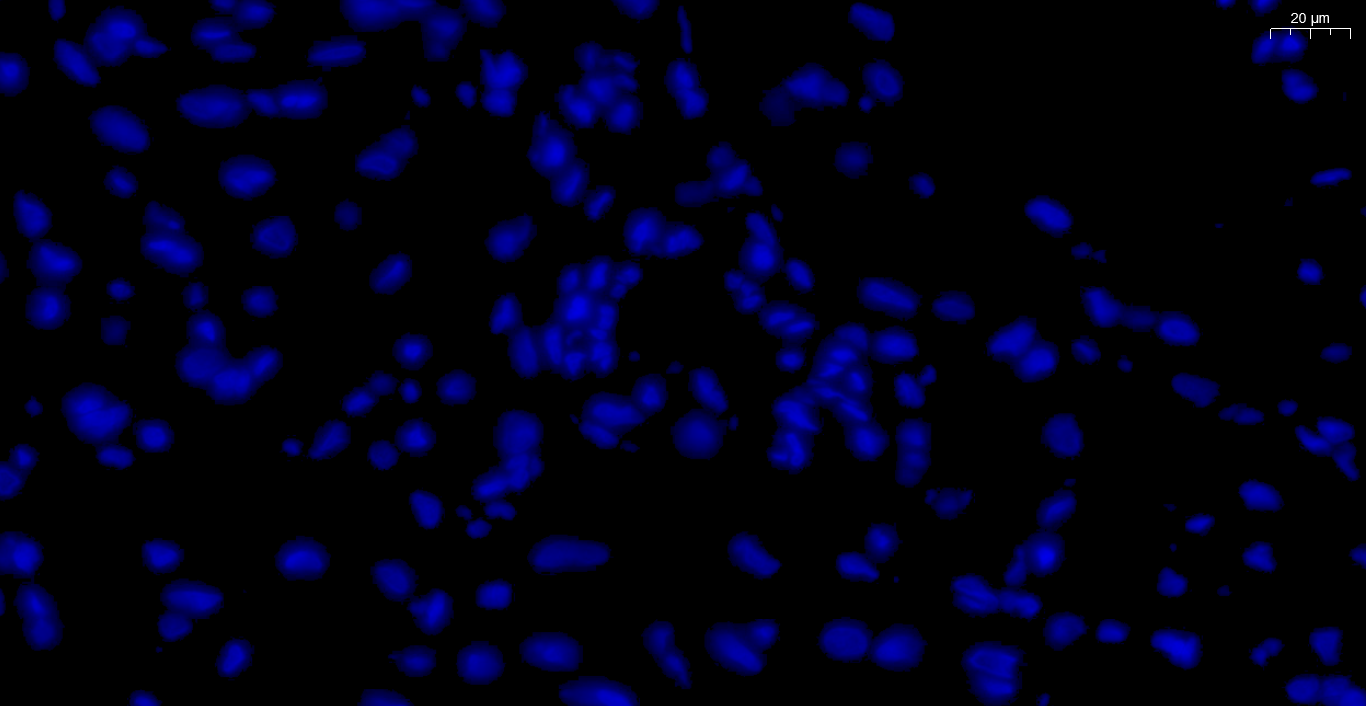

Supplement: Supplementary file 1 [file DataSheet3.ZIP › Raw data-2/Immunofluorescence (IF) Analysis/in vivo/Sham/Sham-DAPI.tif]

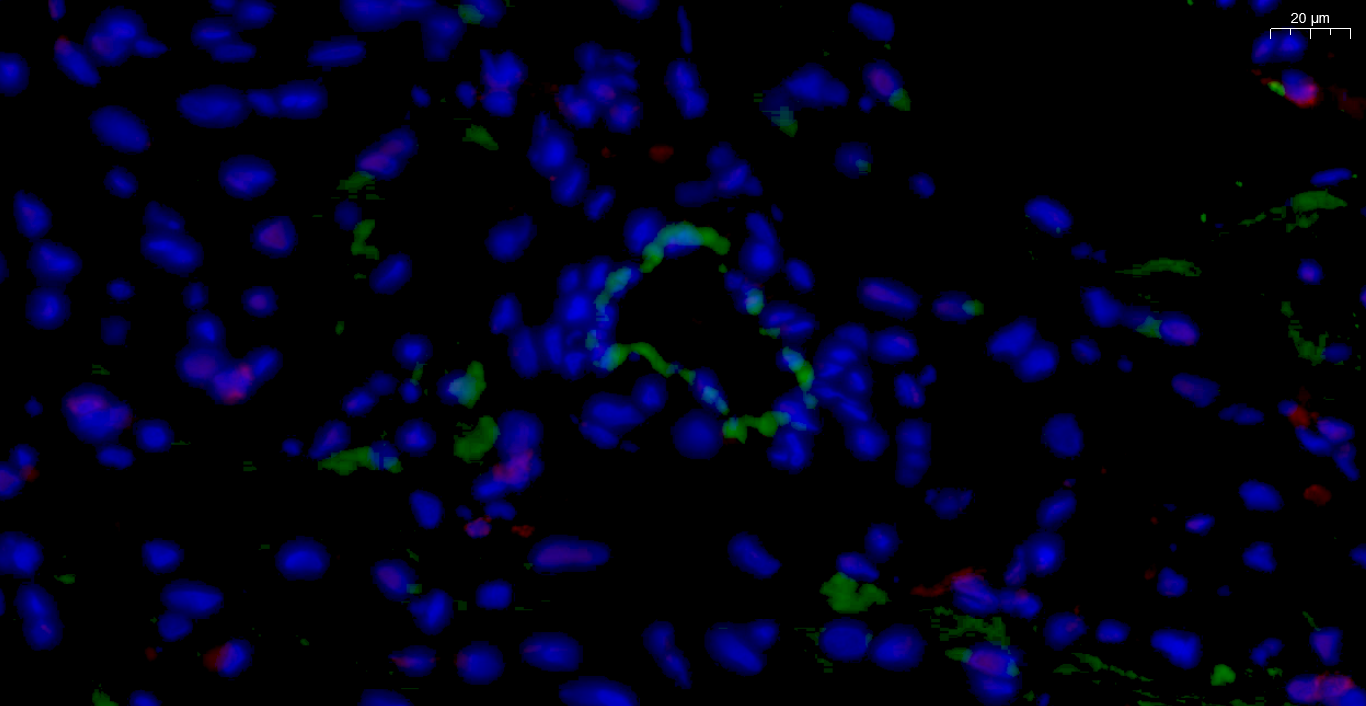

Supplement: Supplementary file 1 [file DataSheet3.ZIP › Raw data-2/Immunofluorescence (IF) Analysis/in vivo/Sham/Sham-MERGE.tif]

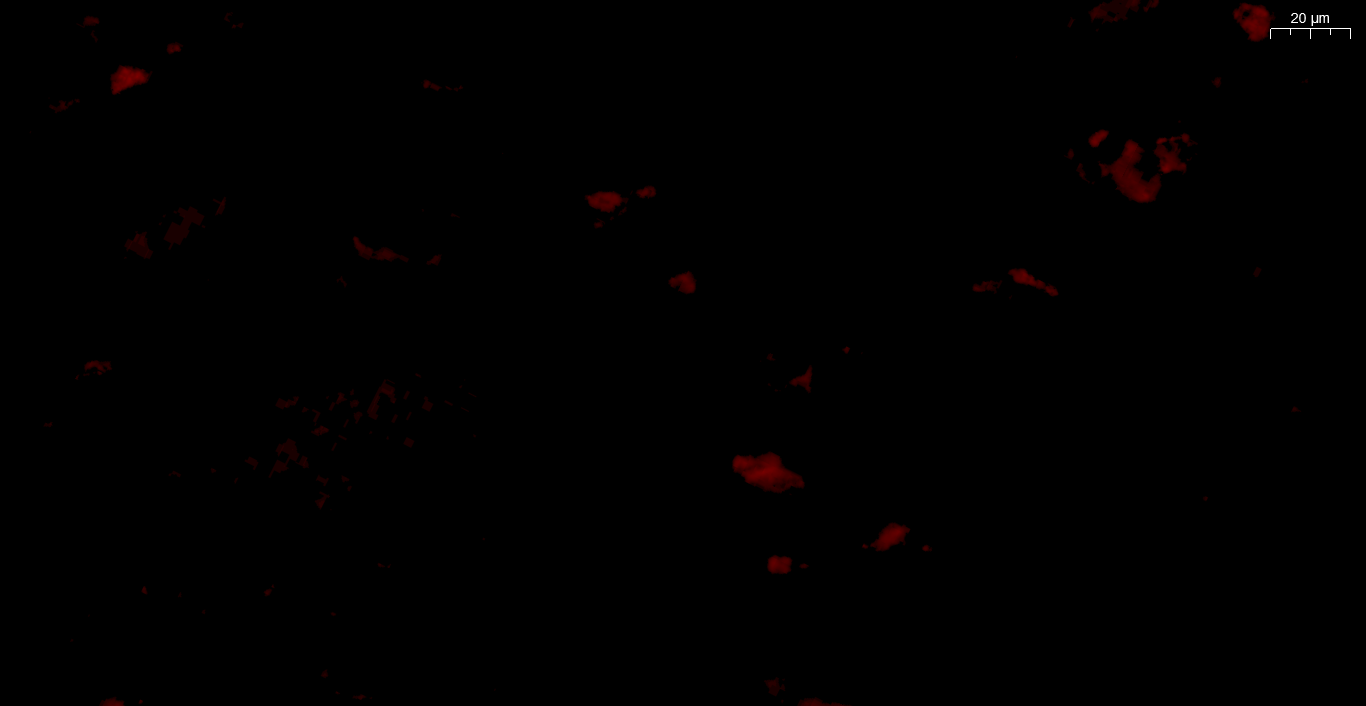

Supplement: Supplementary file 1 [file DataSheet3.ZIP › Raw data-2/Immunofluorescence (IF) Analysis/in vivo/STDP/STDP-CD206.tif]

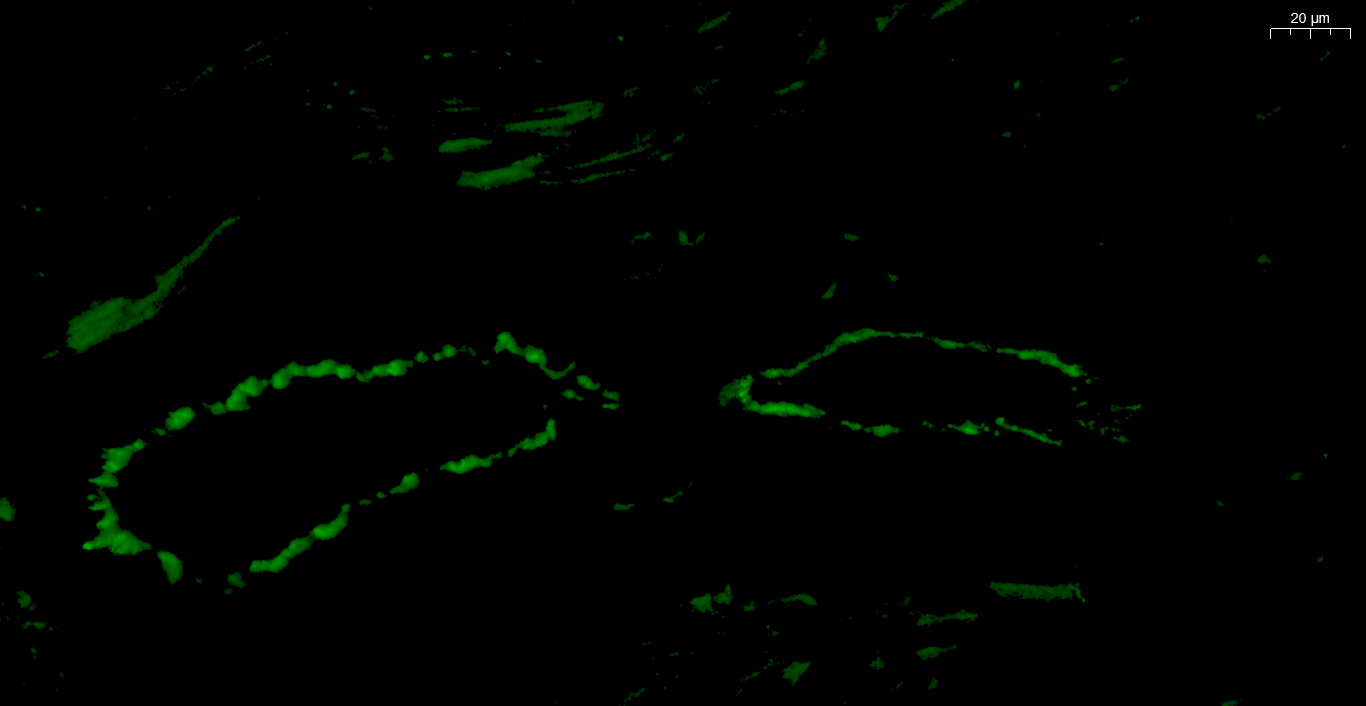

Supplement: Supplementary file 1 [file DataSheet3.ZIP › Raw data-2/Immunofluorescence (IF) Analysis/in vivo/STDP/STDP-CD31.tif]

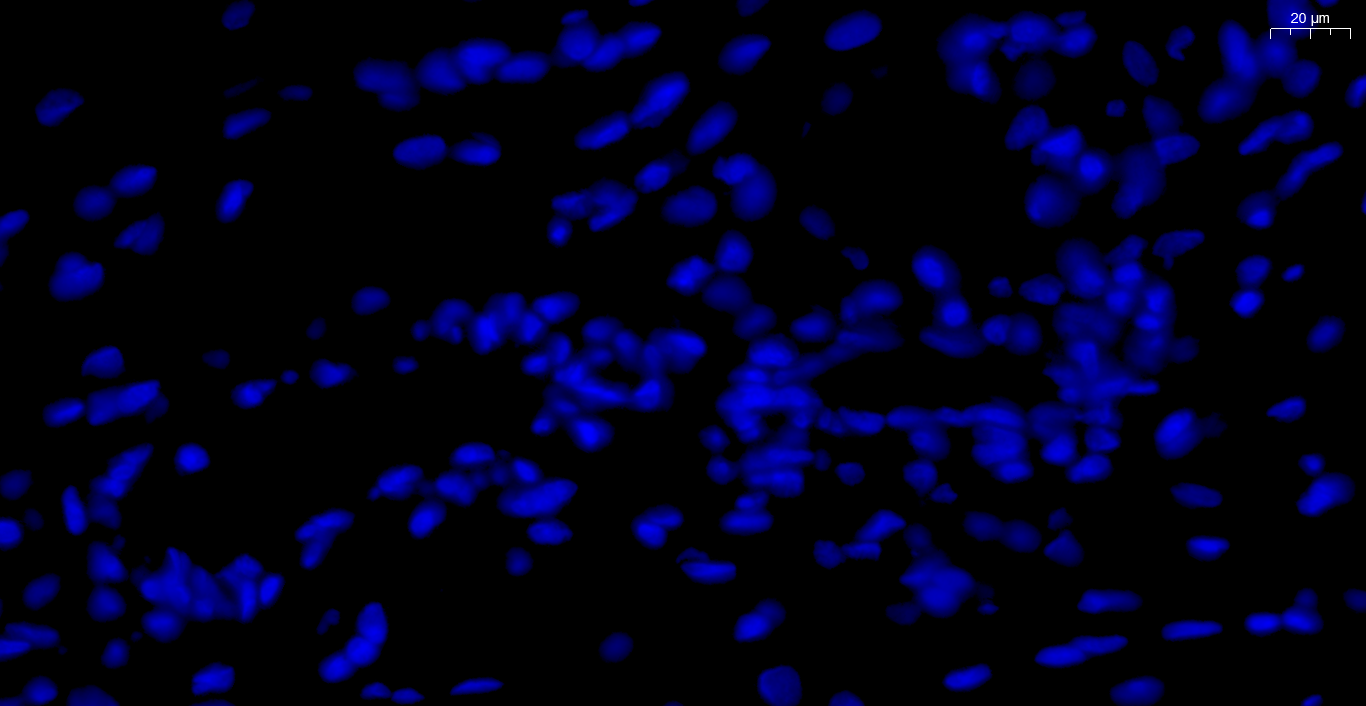

Supplement: Supplementary file 1 [file DataSheet3.ZIP › Raw data-2/Immunofluorescence (IF) Analysis/in vivo/STDP/STDP-DAPI.tif]

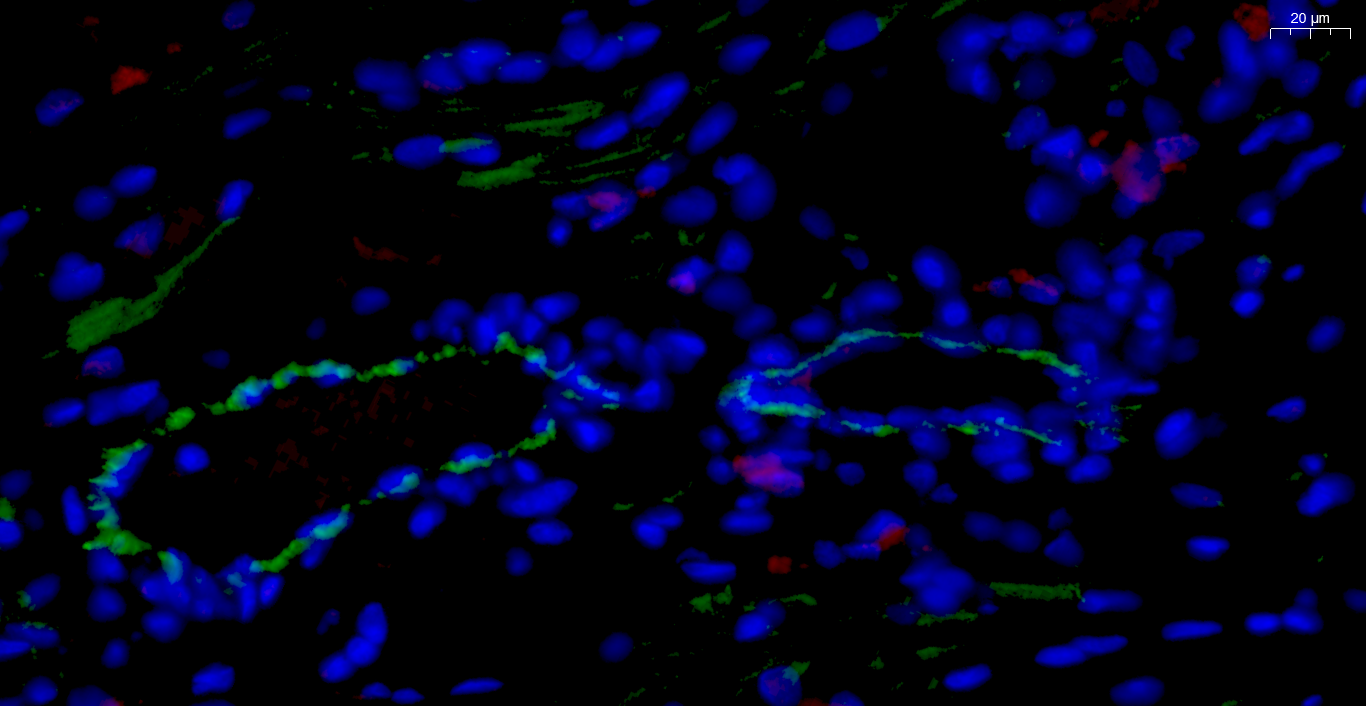

Supplement: Supplementary file 1 [file DataSheet3.ZIP › Raw data-2/Immunofluorescence (IF) Analysis/in vivo/STDP/STDP-MERGE.tif]

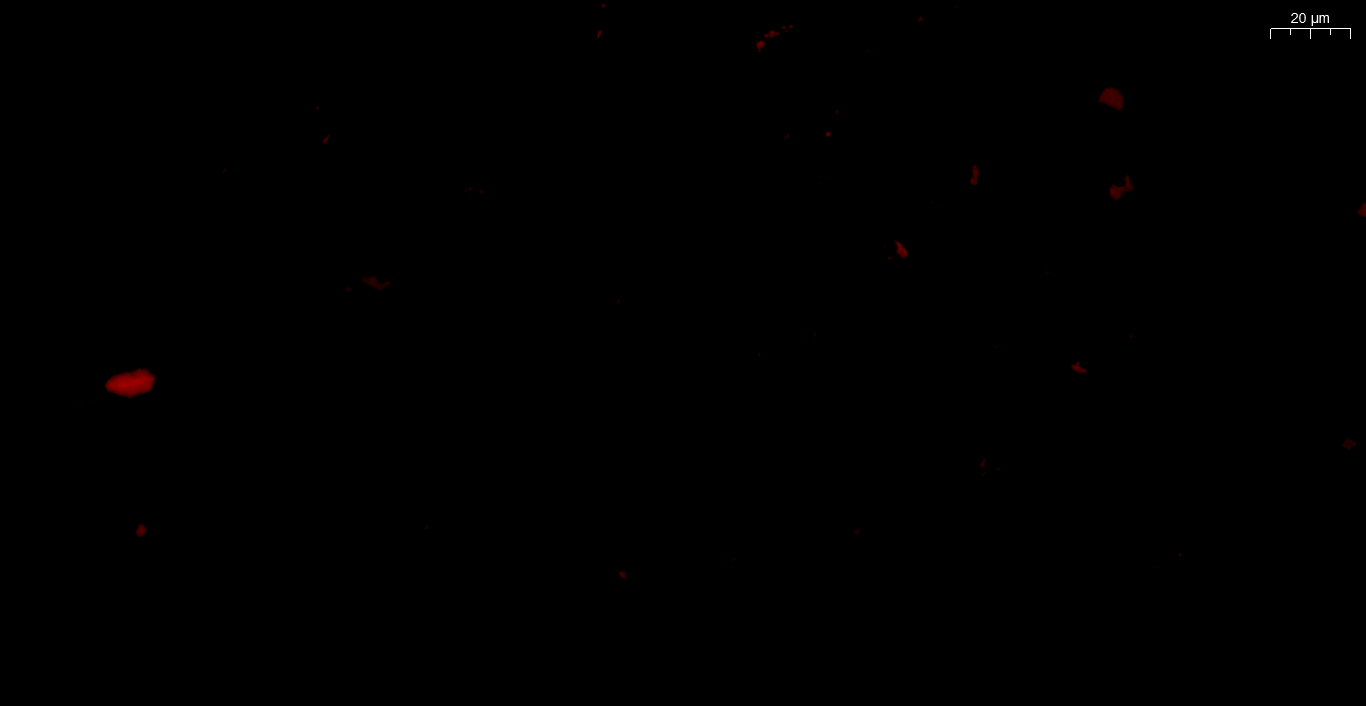

Supplement: Supplementary file 1 [file DataSheet3.ZIP › Raw data-2/Immunofluorescence (IF) Analysis/in vivo/TLM/TLM-CD206.tif]

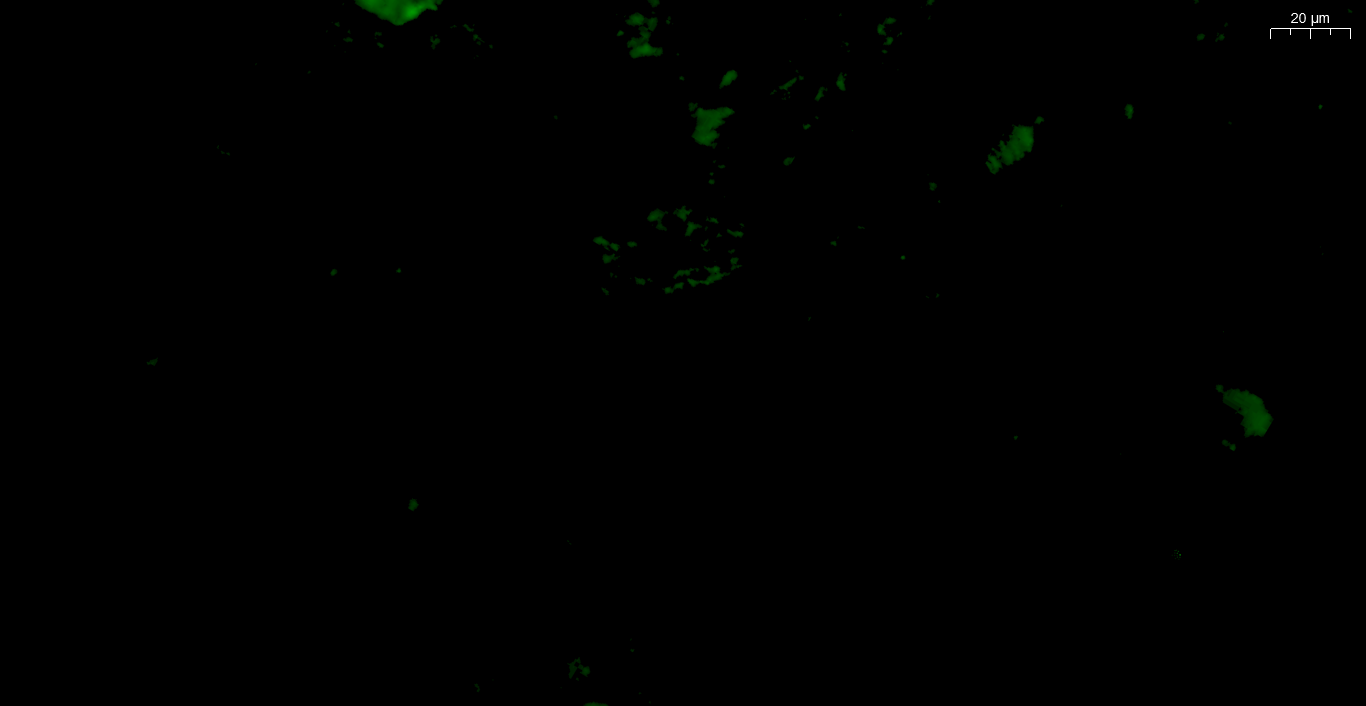

Supplement: Supplementary file 1 [file DataSheet3.ZIP › Raw data-2/Immunofluorescence (IF) Analysis/in vivo/TLM/TLM-CD31.tif]

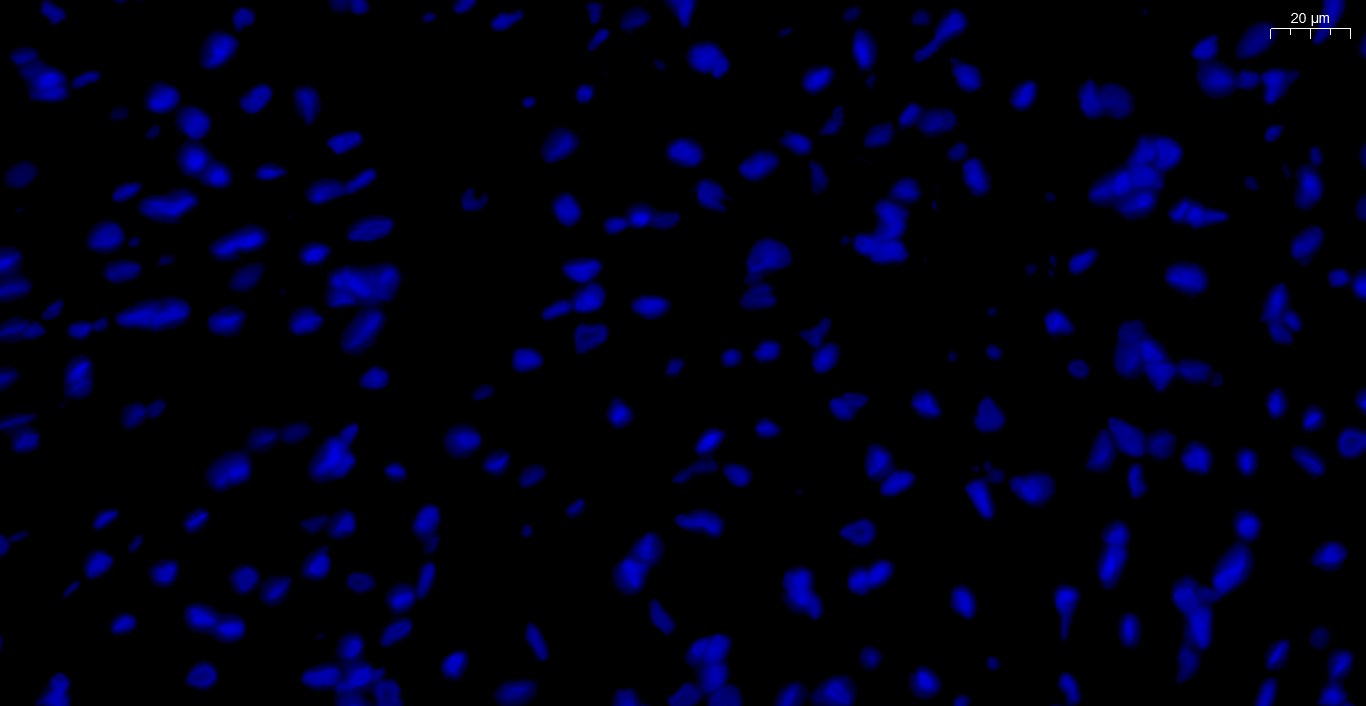

Supplement: Supplementary file 1 [file DataSheet3.ZIP › Raw data-2/Immunofluorescence (IF) Analysis/in vivo/TLM/TLM-DAPI.tif]

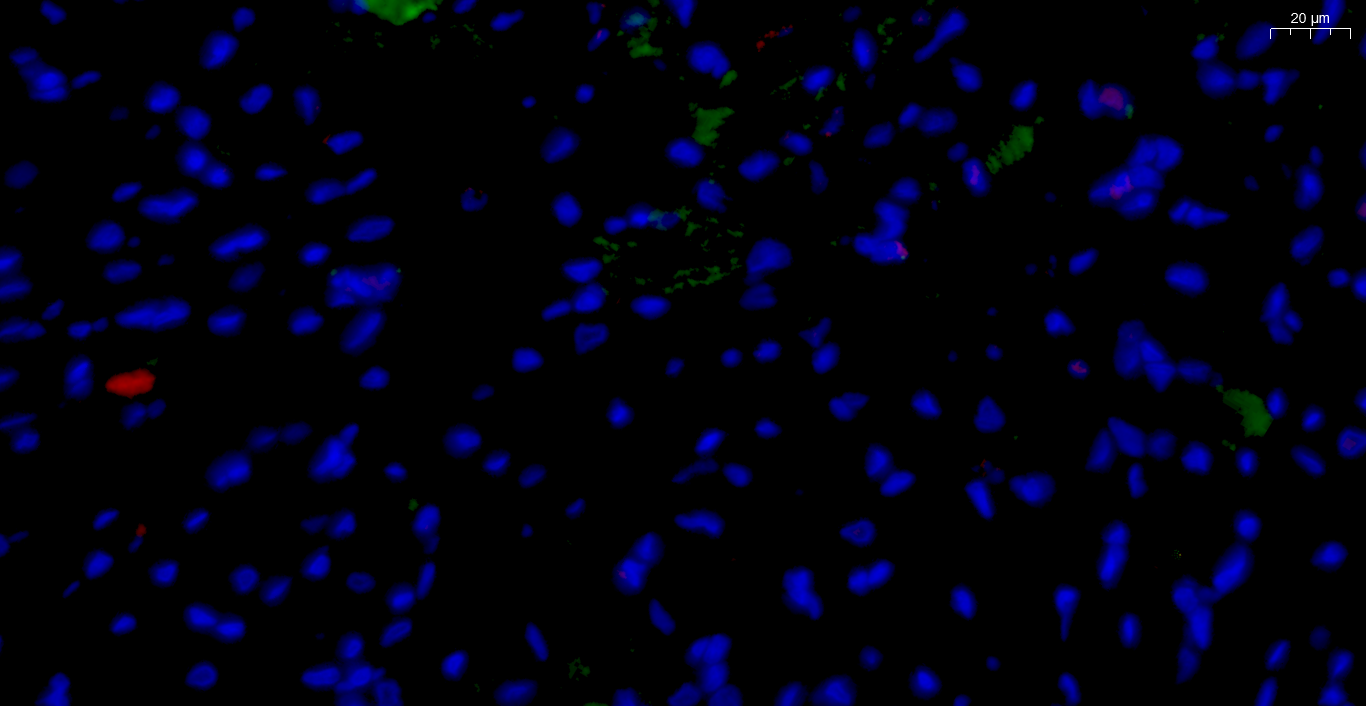

Supplement: Supplementary file 1 [file DataSheet3.ZIP › Raw data-2/Immunofluorescence (IF) Analysis/in vivo/TLM/TLM-MERGE.tif]

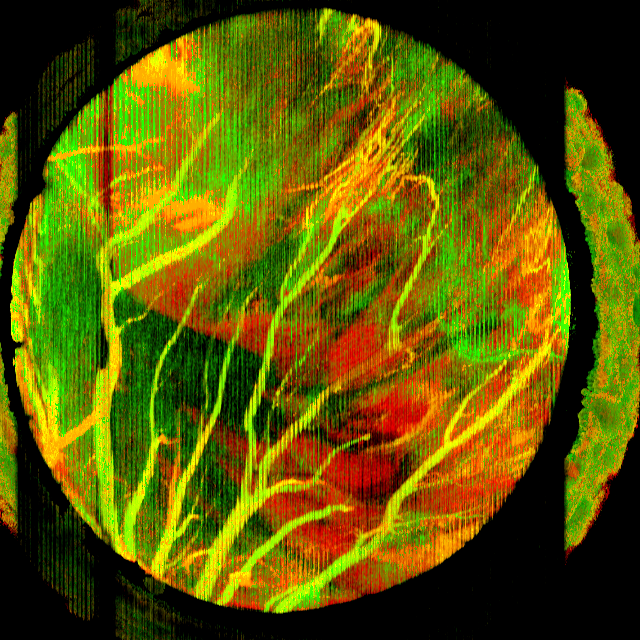

Supplement: Supplementary file 1 [file DataSheet3.ZIP › Raw data-2/OMAG/OMAG/Model.png]

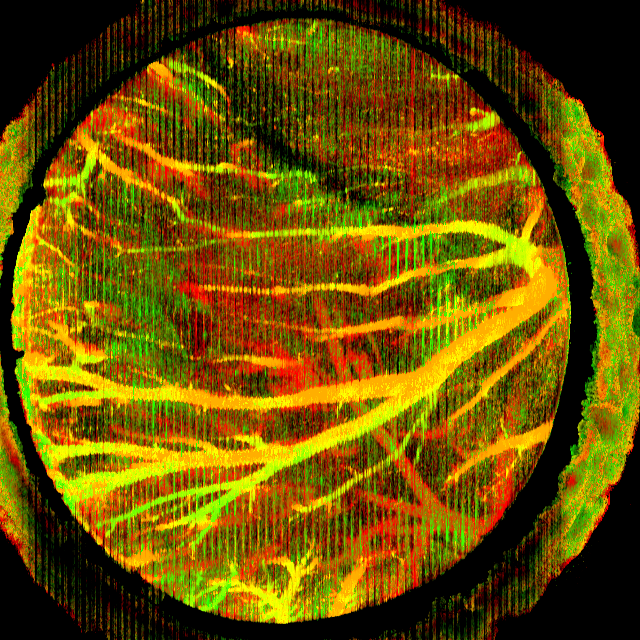

Supplement: Supplementary file 1 [file DataSheet3.ZIP › Raw data-2/OMAG/OMAG/Sham.png]

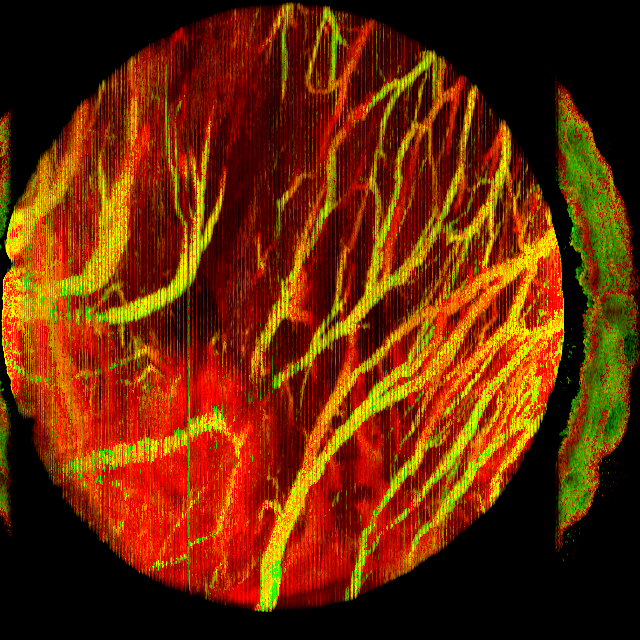

Supplement: Supplementary file 1 [file DataSheet3.ZIP › Raw data-2/OMAG/OMAG/STDP.png]

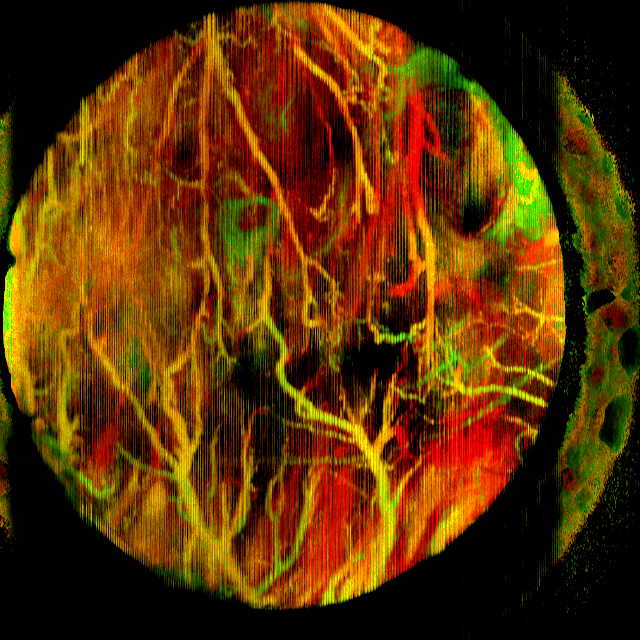

Supplement: Supplementary file 1 [file DataSheet3.ZIP › Raw data-2/OMAG/OMAG/TLM.png]

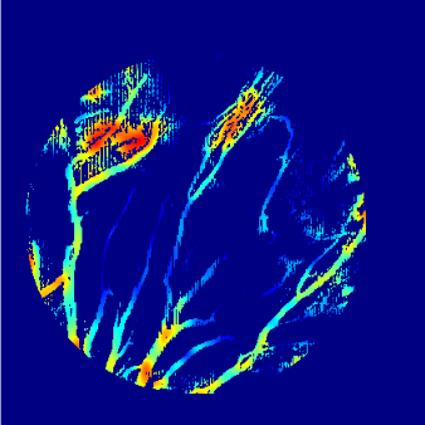

Supplement: Supplementary file 1 [file DataSheet3.ZIP › Raw data-2/OMAG/Vessel Area Density/Model.png]

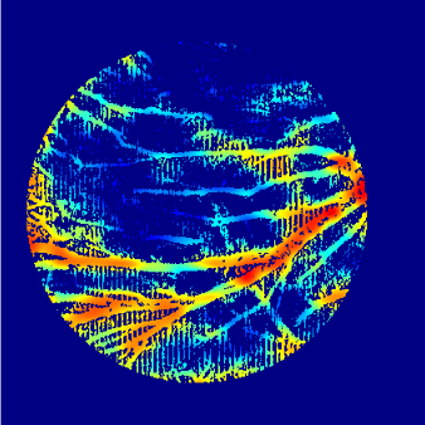

Supplement: Supplementary file 1 [file DataSheet3.ZIP › Raw data-2/OMAG/Vessel Area Density/Sham.png]

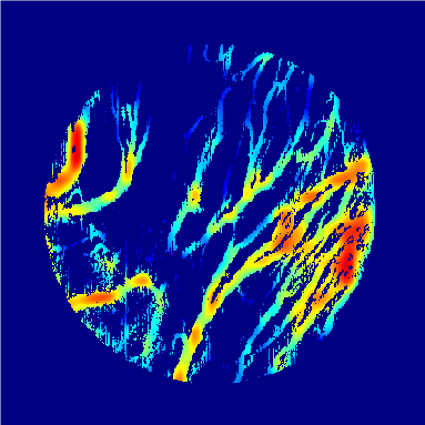

Supplement: Supplementary file 1 [file DataSheet3.ZIP › Raw data-2/OMAG/Vessel Area Density/STDP.png]

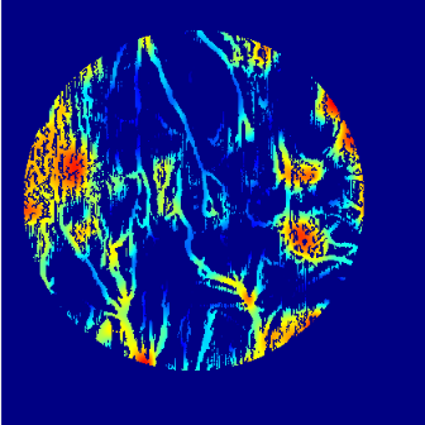

Supplement: Supplementary file 1 [file DataSheet3.ZIP › Raw data-2/OMAG/Vessel Area Density/TLM.png]

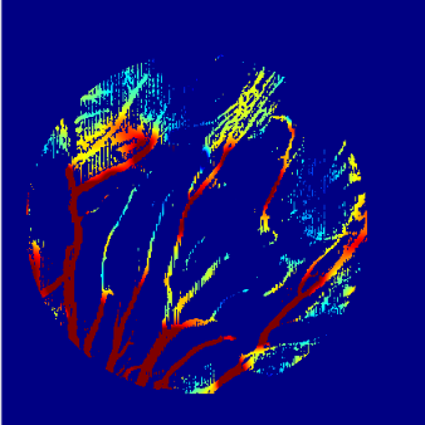

Supplement: Supplementary file 1 [file DataSheet3.ZIP › Raw data-2/OMAG/Vessel Diameter Index/Model.png]

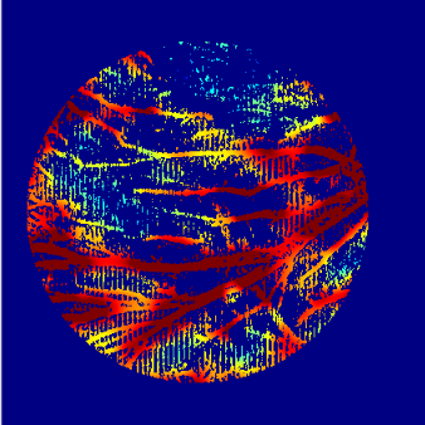

Supplement: Supplementary file 1 [file DataSheet3.ZIP › Raw data-2/OMAG/Vessel Diameter Index/Sham.png]

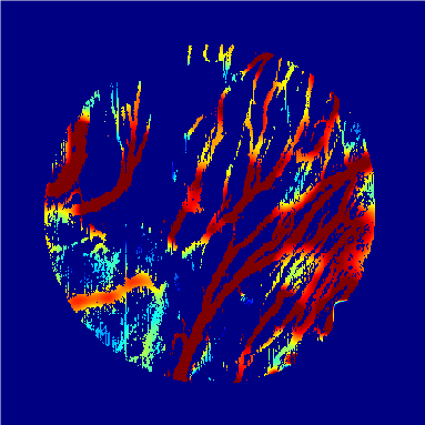

Supplement: Supplementary file 1 [file DataSheet3.ZIP › Raw data-2/OMAG/Vessel Diameter Index/STDP.png]

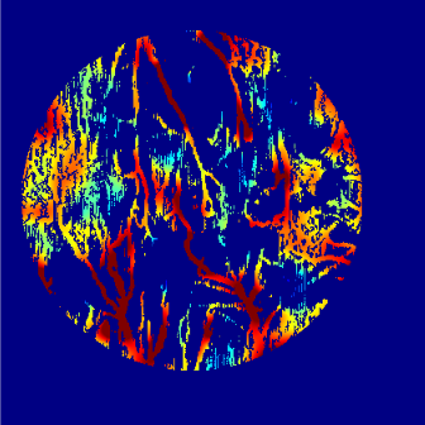

Supplement: Supplementary file 1 [file DataSheet3.ZIP › Raw data-2/OMAG/Vessel Diameter Index/TLM.png]

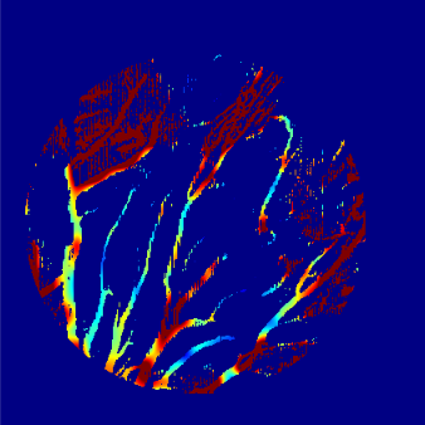

Supplement: Supplementary file 1 [file DataSheet3.ZIP › Raw data-2/OMAG/Vessel Skeleton Density/Model.png]

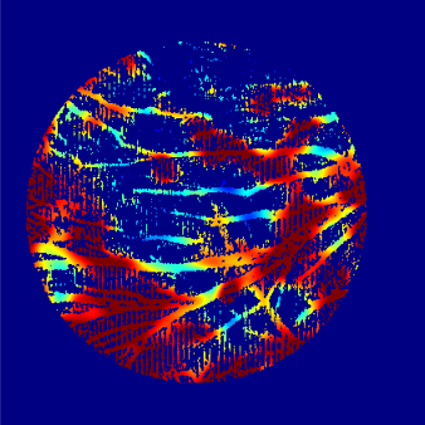

Supplement: Supplementary file 1 [file DataSheet3.ZIP › Raw data-2/OMAG/Vessel Skeleton Density/Sham.png]

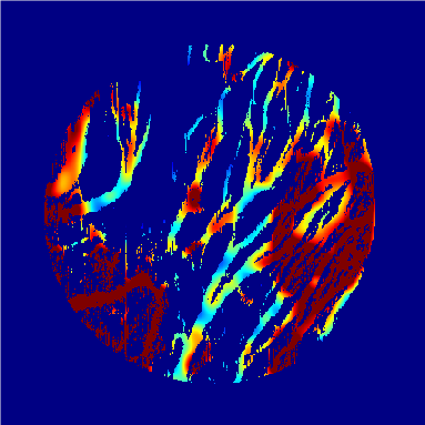

Supplement: Supplementary file 1 [file DataSheet3.ZIP › Raw data-2/OMAG/Vessel Skeleton Density/STDP.png]

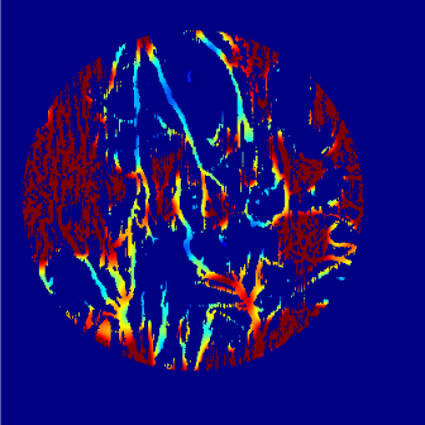

Supplement: Supplementary file 1 [file DataSheet3.ZIP › Raw data-2/OMAG/Vessel Skeleton Density/TLM.png]

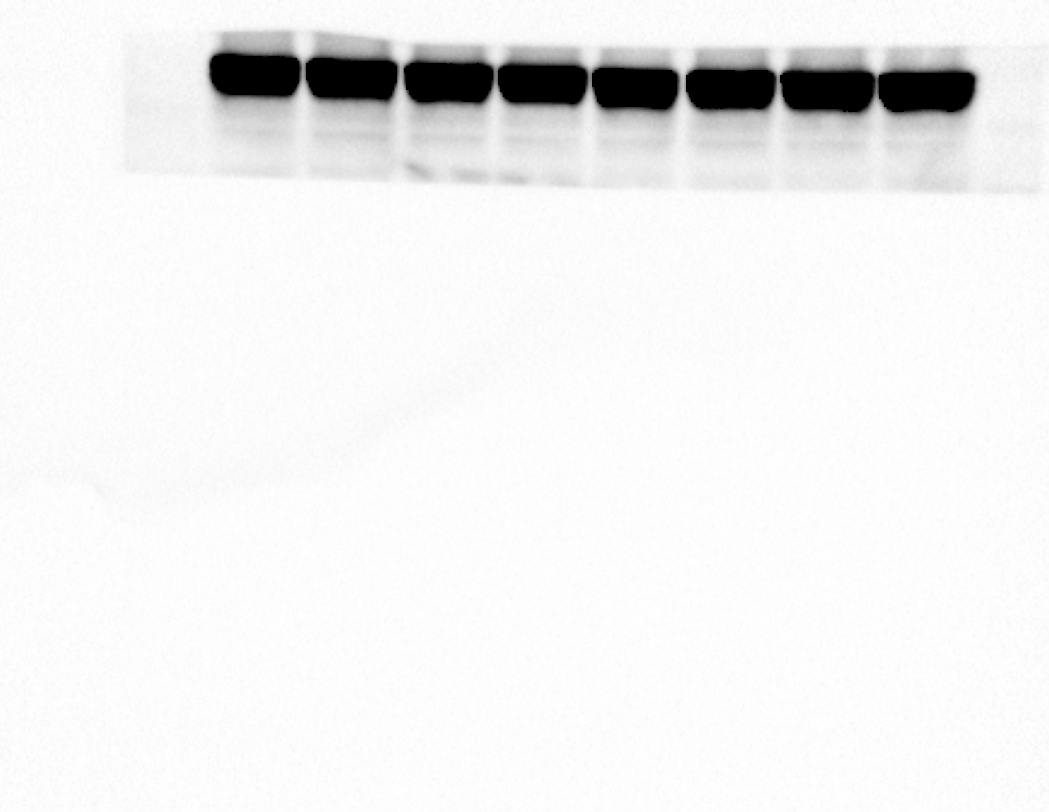

Supplement: Supplementary file 1 [file DataSheet3.ZIP › Raw data-2/Western Blot/in vivo/Akt/Akt(1,2).tif]

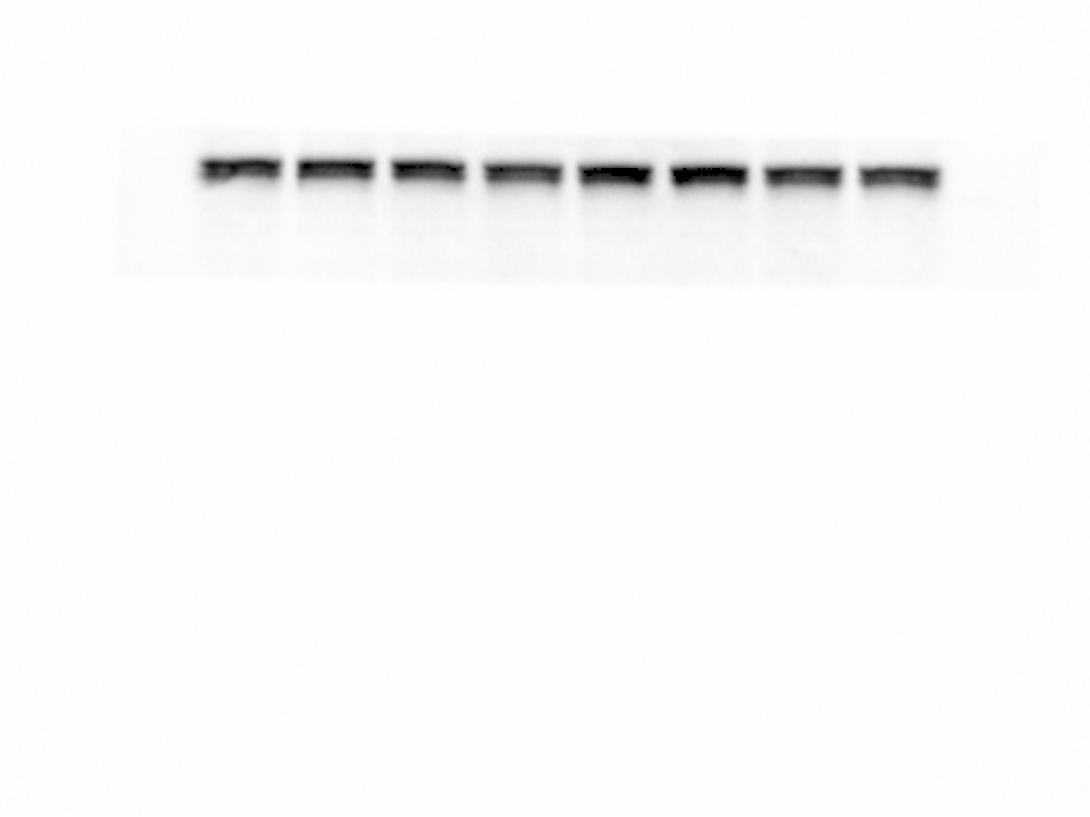

Supplement: Supplementary file 1 [file DataSheet3.ZIP › Raw data-2/Western Blot/in vivo/Akt/Akt(3.4).tif]

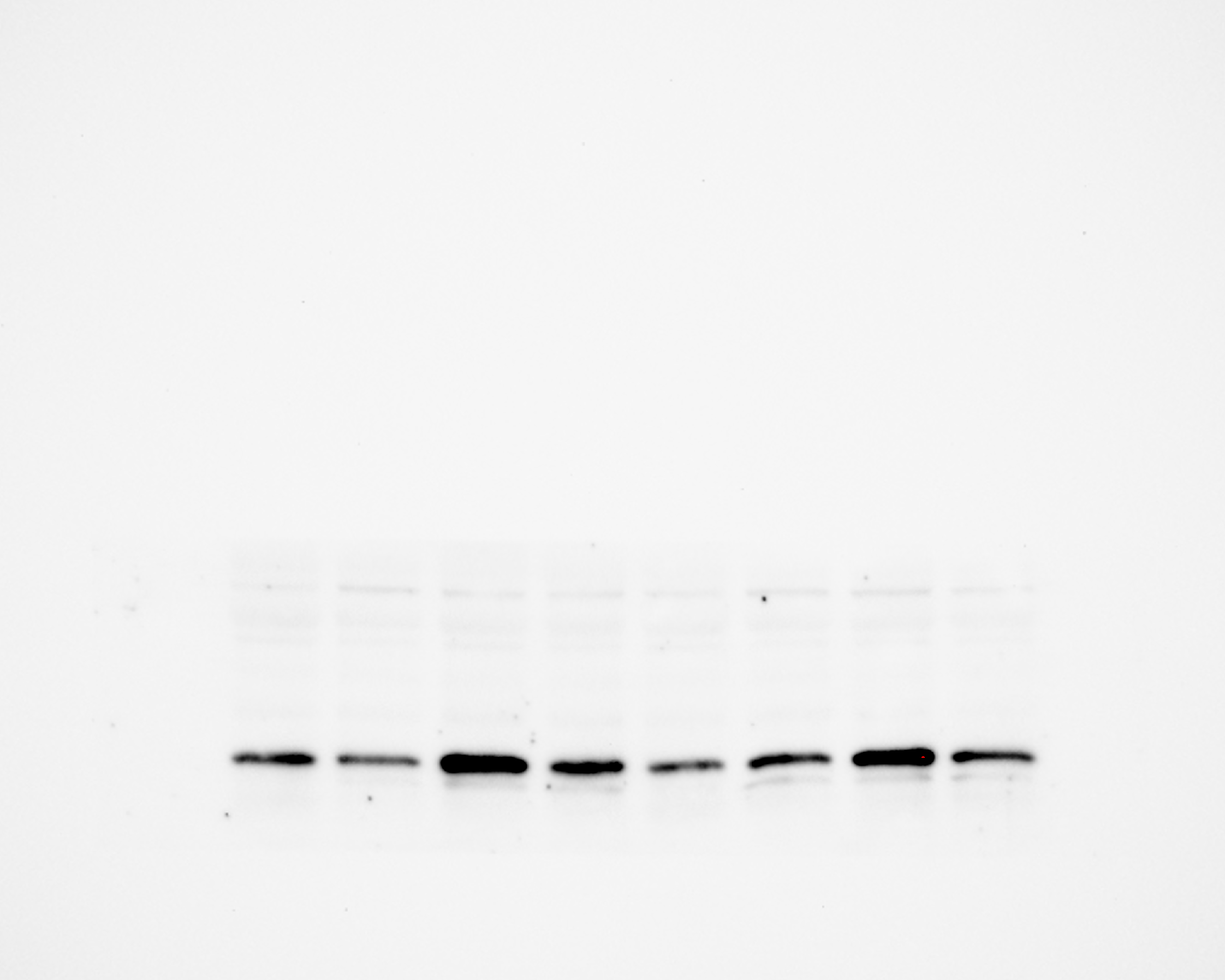

Supplement: Supplementary file 1 [file DataSheet3.ZIP › Raw data-2/Western Blot/in vivo/Arg-1/Arg-1(1,2).tif]

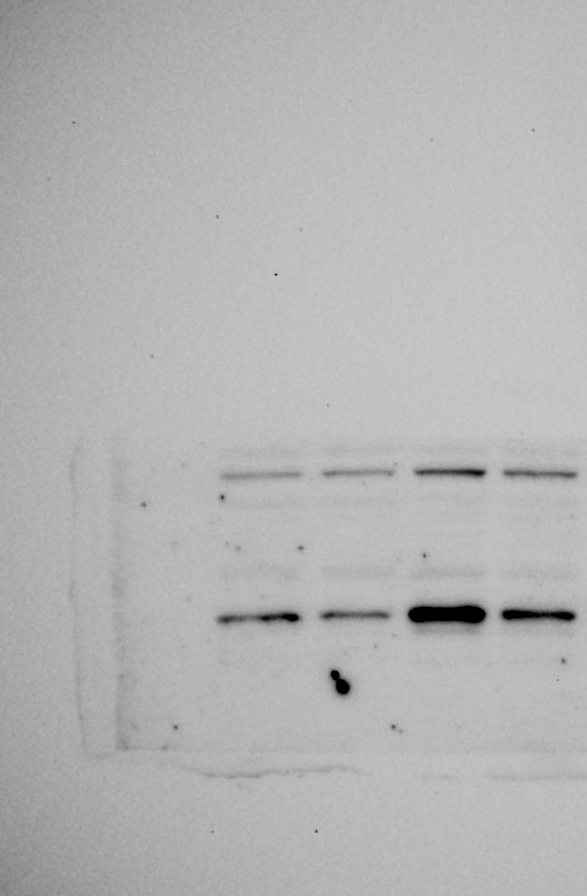

Supplement: Supplementary file 1 [file DataSheet3.ZIP › Raw data-2/Western Blot/in vivo/Arg-1/Arg-1(3).tif]

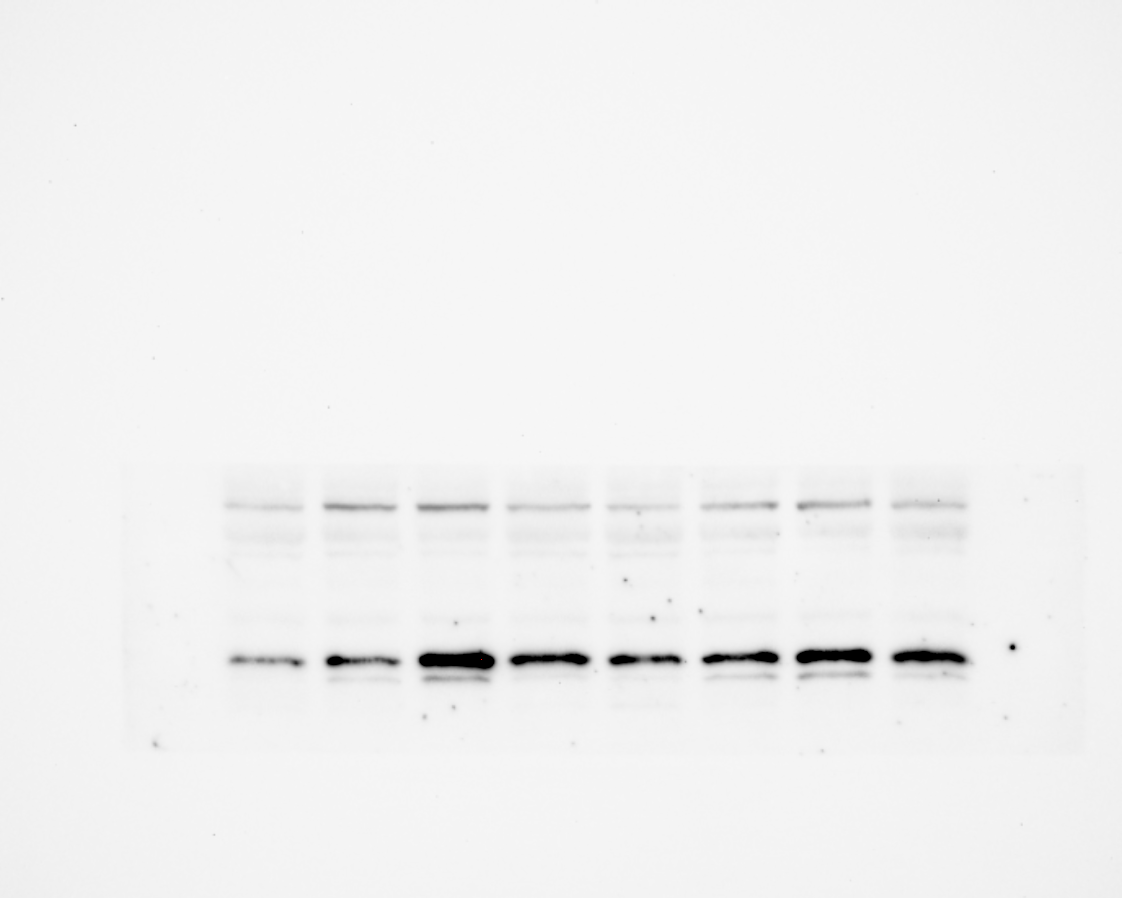

Supplement: Supplementary file 1 [file DataSheet3.ZIP › Raw data-2/Western Blot/in vivo/Arg-1/Arg-1(4,5).tif]

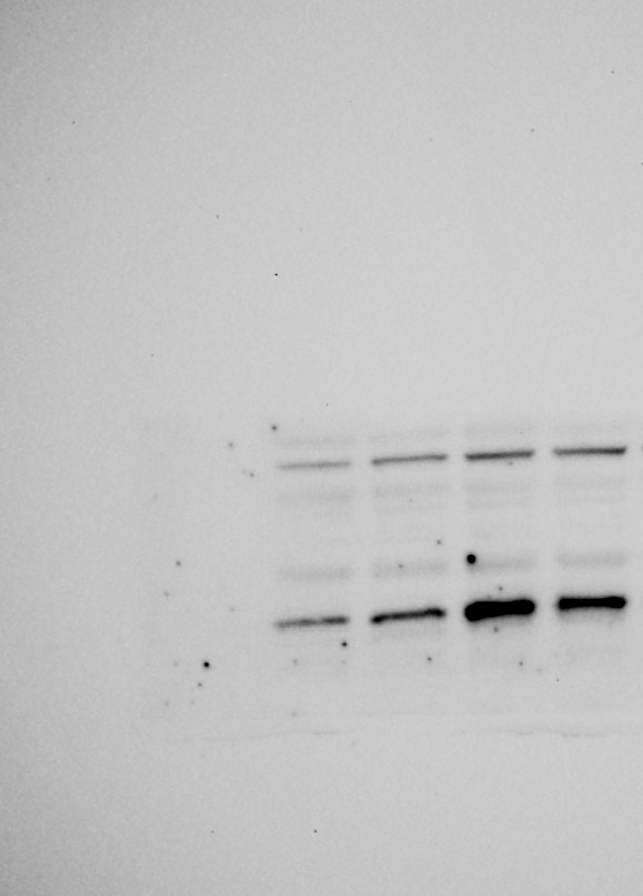

Supplement: Supplementary file 1 [file DataSheet3.ZIP › Raw data-2/Western Blot/in vivo/Arg-1/Arg-1(6).tif]

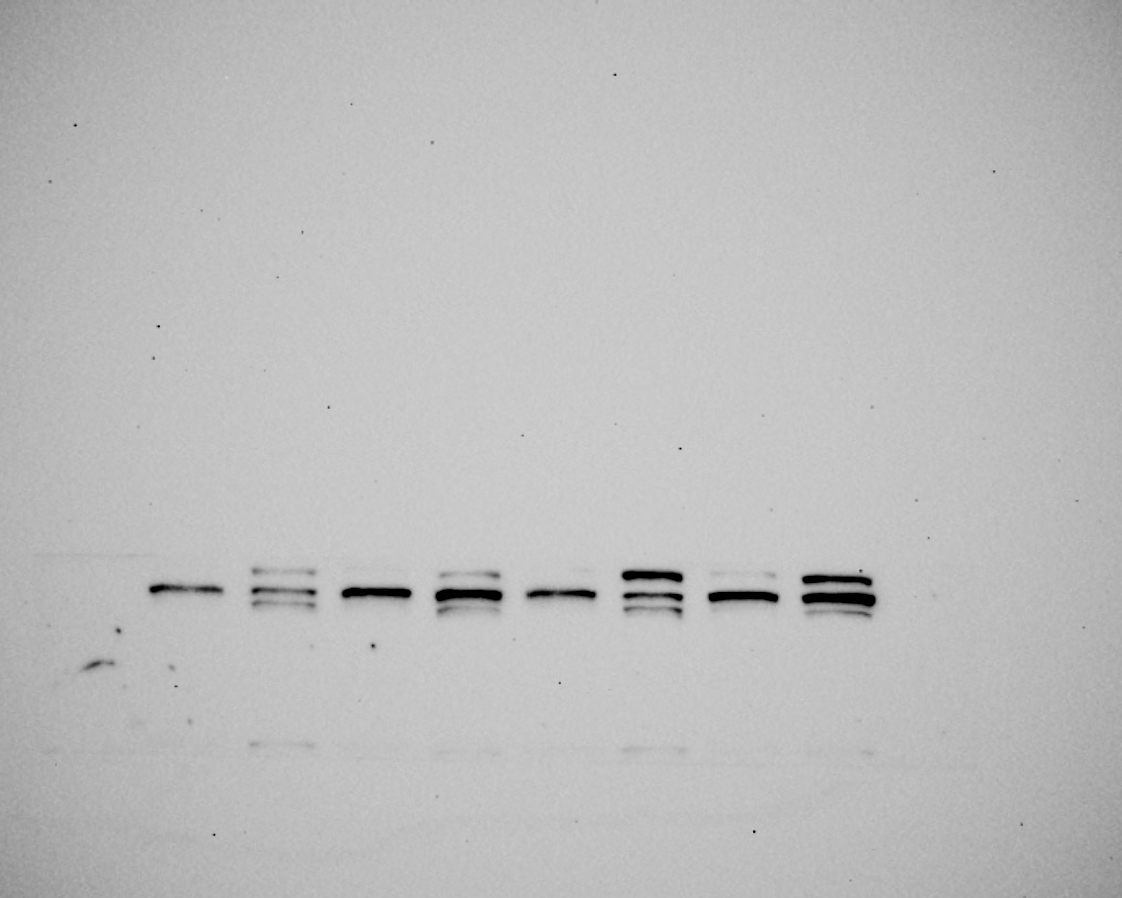

Supplement: Supplementary file 1 [file DataSheet3.ZIP › Raw data-2/Western Blot/in vivo/CD206/CD206(1,2).tif]

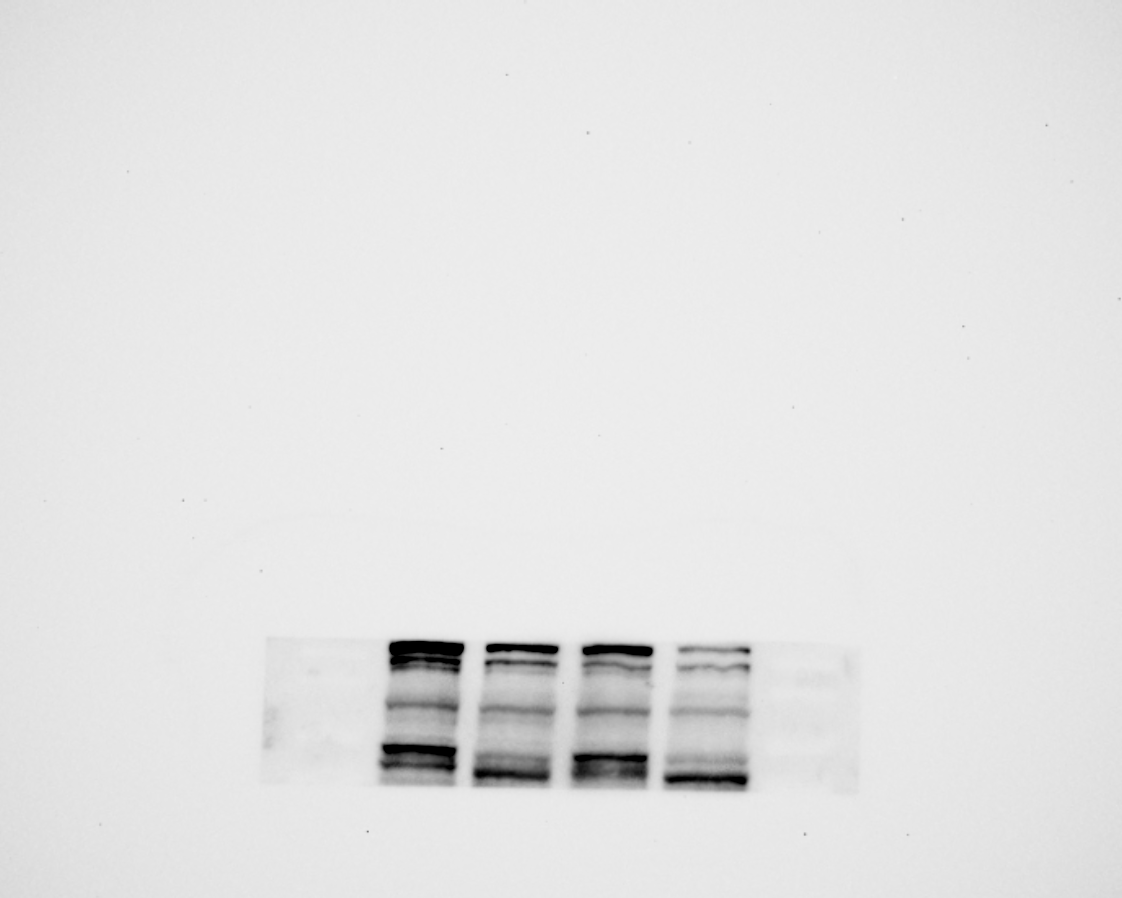

Supplement: Supplementary file 1 [file DataSheet3.ZIP › Raw data-2/Western Blot/in vivo/CD206/CD206(3).tif]

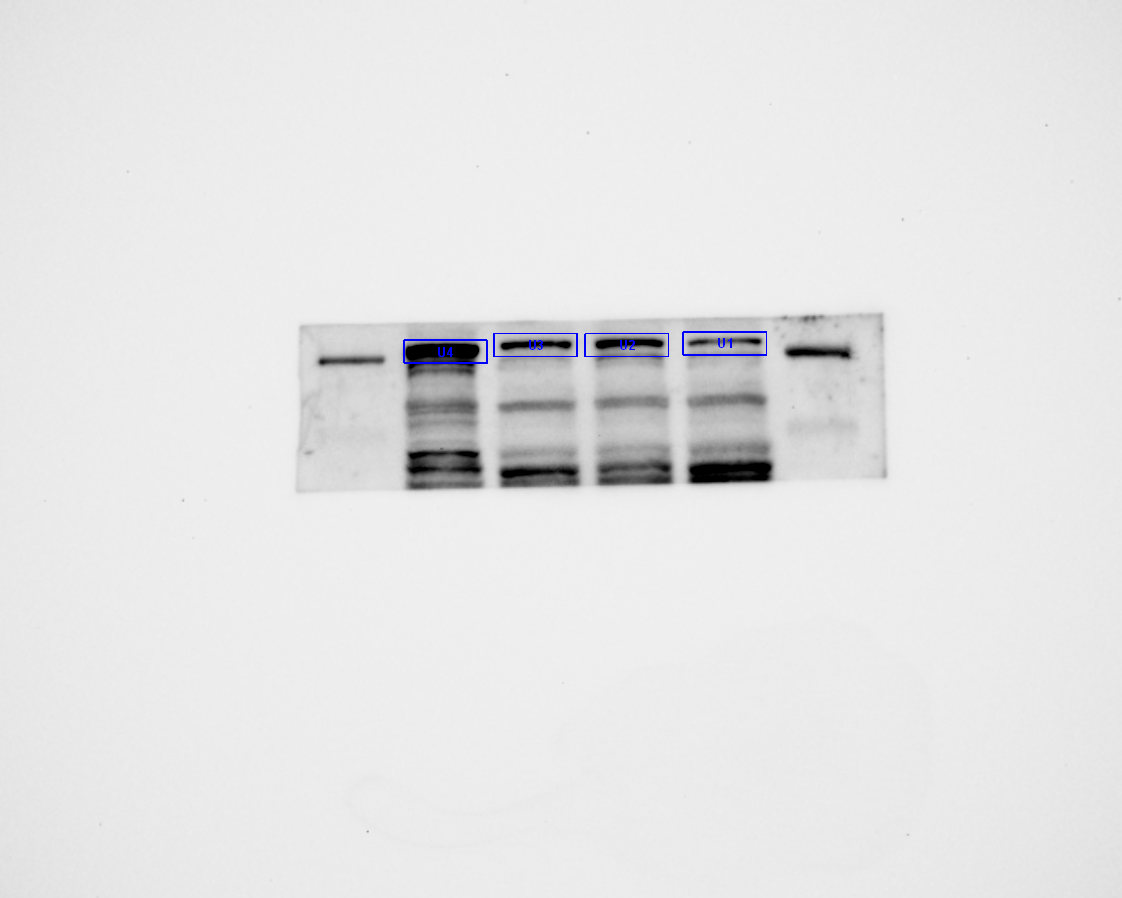

Supplement: Supplementary file 1 [file DataSheet3.ZIP › Raw data-2/Western Blot/in vivo/CD206/CD206(4).tif]

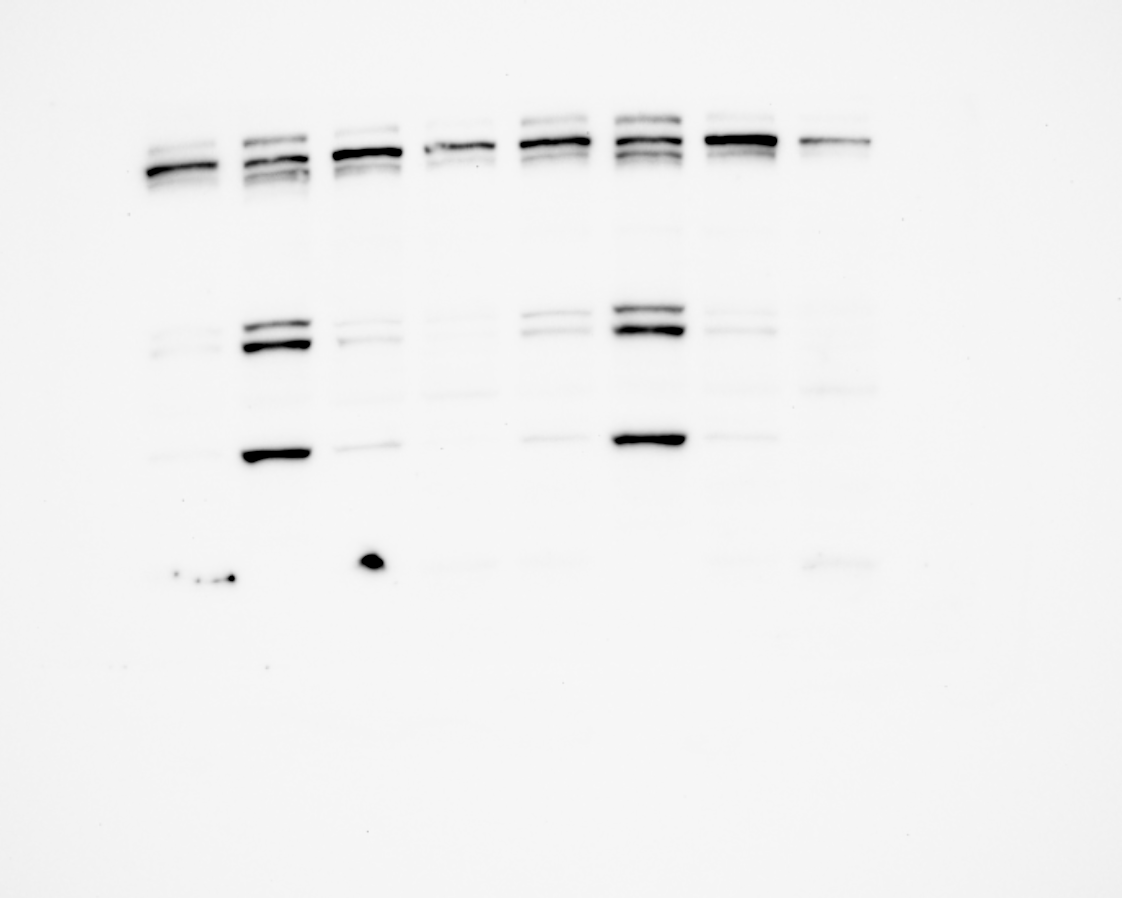

Supplement: Supplementary file 1 [file DataSheet3.ZIP › Raw data-2/Western Blot/in vivo/CD206/CD206(5,6).tif]

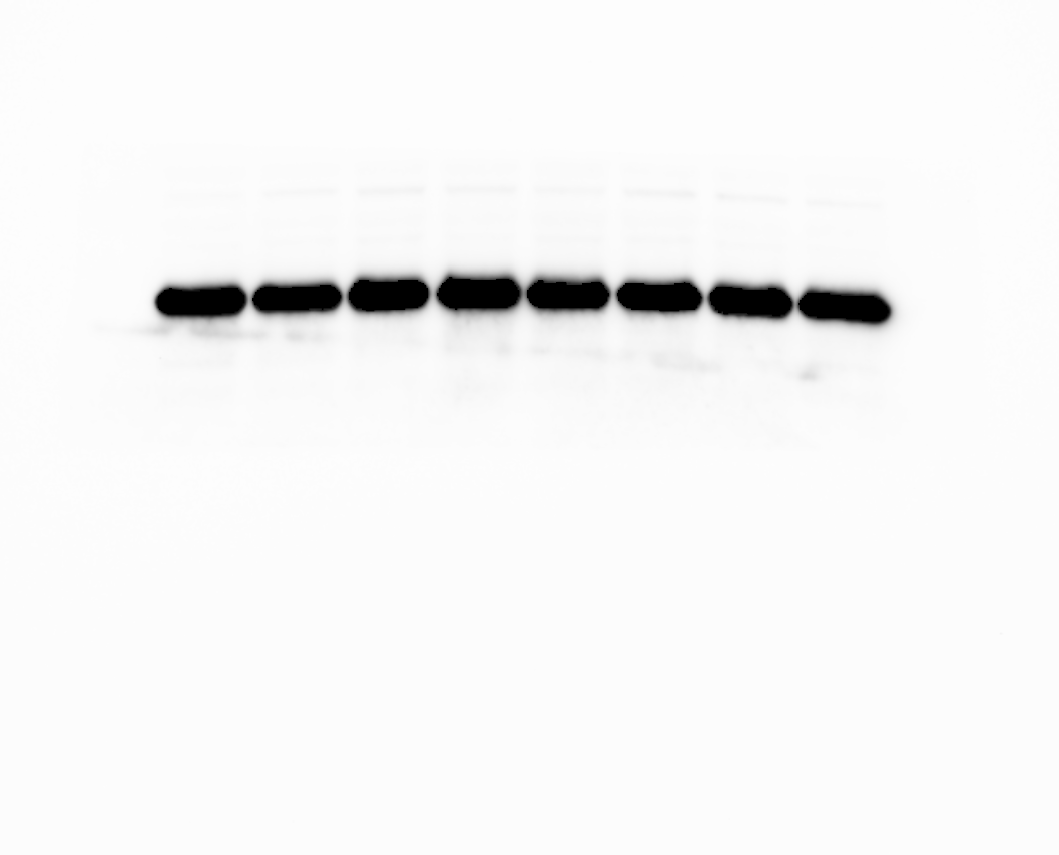

Supplement: Supplementary file 1 [file DataSheet3.ZIP › Raw data-2/Western Blot/in vivo/GAPDH/GAPDH(1,2).tif]

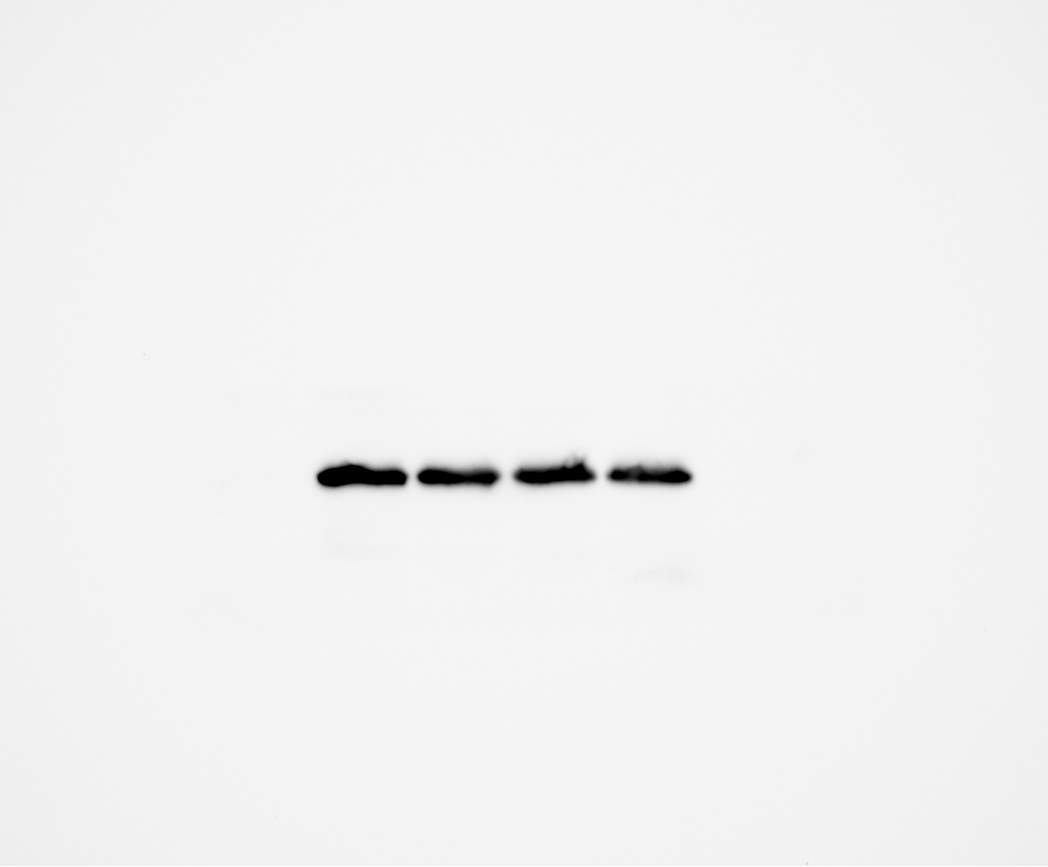

Supplement: Supplementary file 1 [file DataSheet3.ZIP › Raw data-2/Western Blot/in vivo/GAPDH/GAPDH(3).tif]

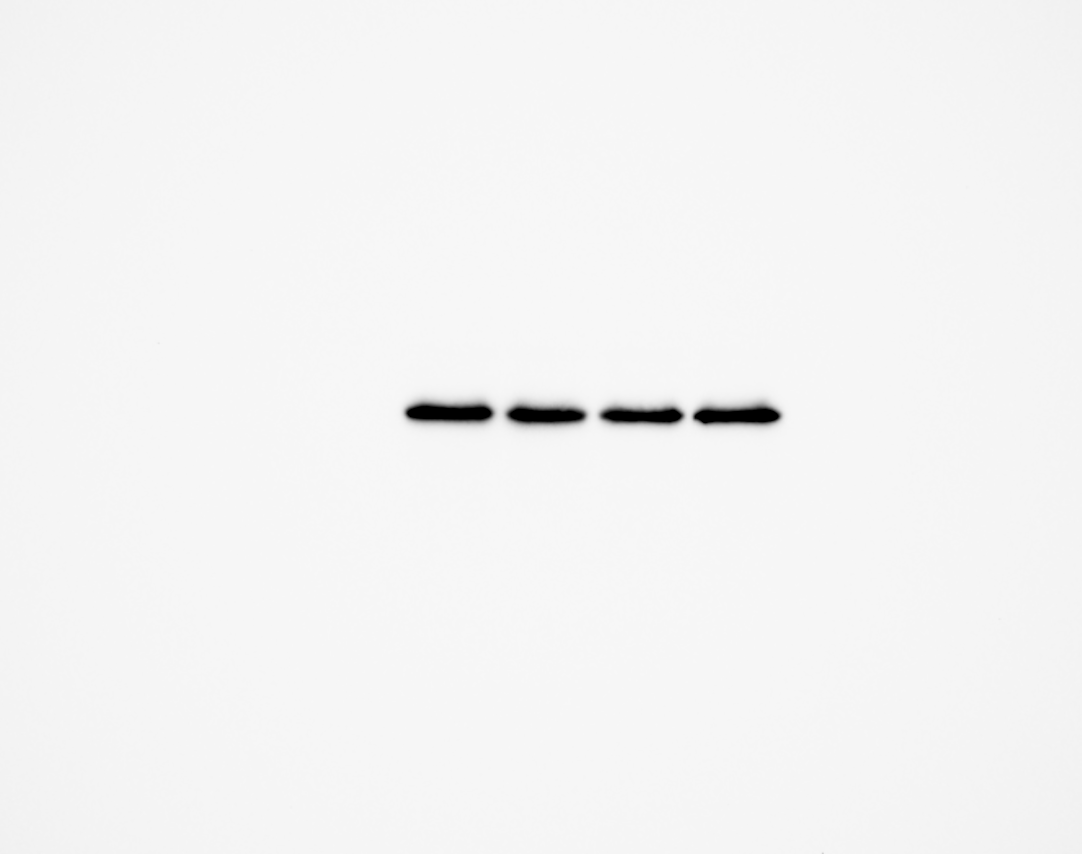

Supplement: Supplementary file 1 [file DataSheet3.ZIP › Raw data-2/Western Blot/in vivo/GAPDH/GAPDH(4).tif]

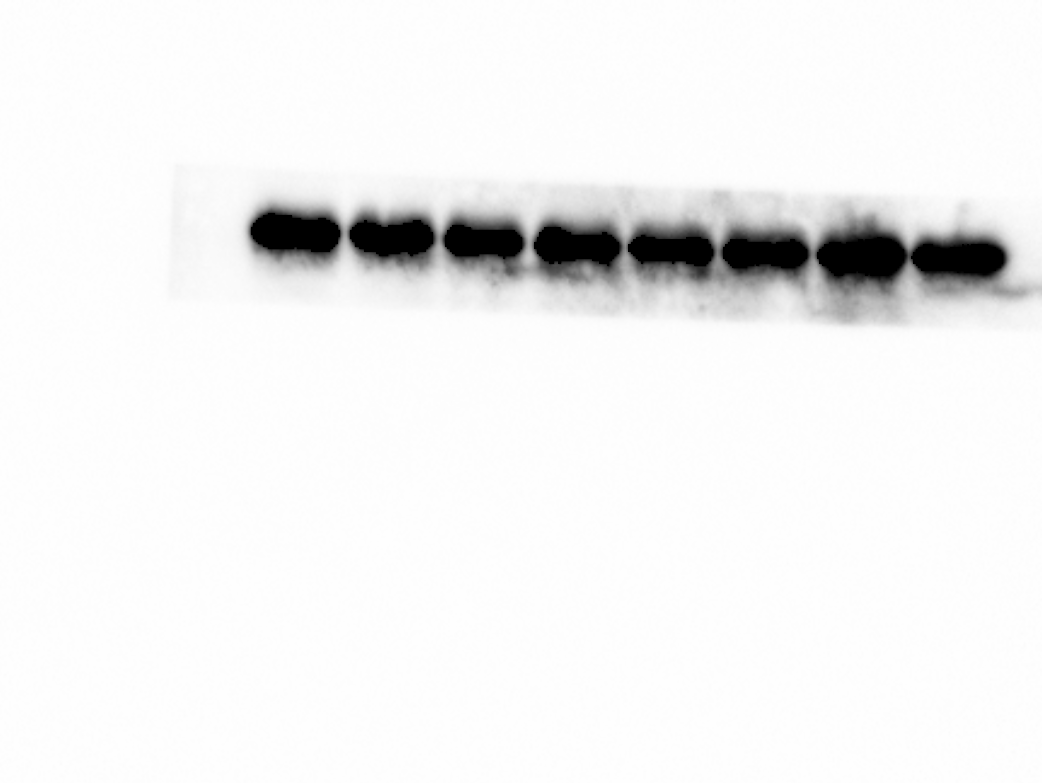

Supplement: Supplementary file 1 [file DataSheet3.ZIP › Raw data-2/Western Blot/in vivo/GAPDH/GAPDH(5,6).tif]

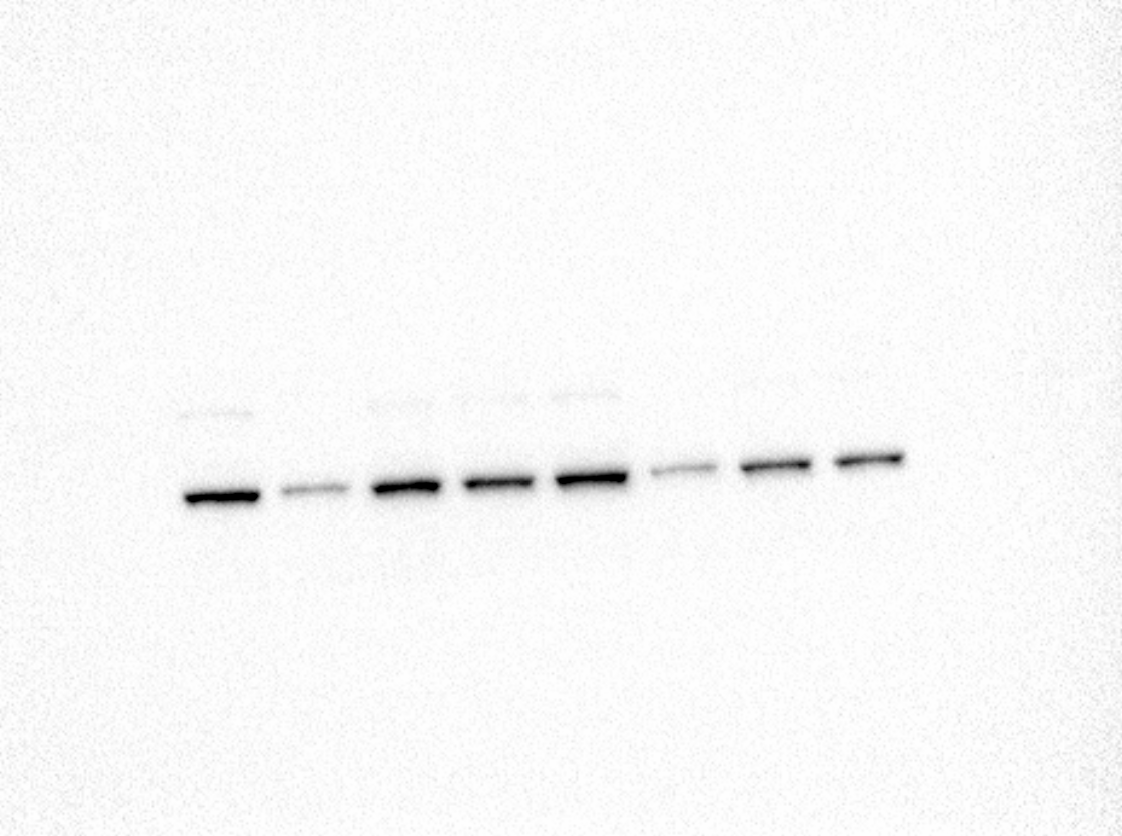

Supplement: Supplementary file 1 [file DataSheet3.ZIP › Raw data-2/Western Blot/in vivo/mTORC1/mTORC1(1,2).tif]

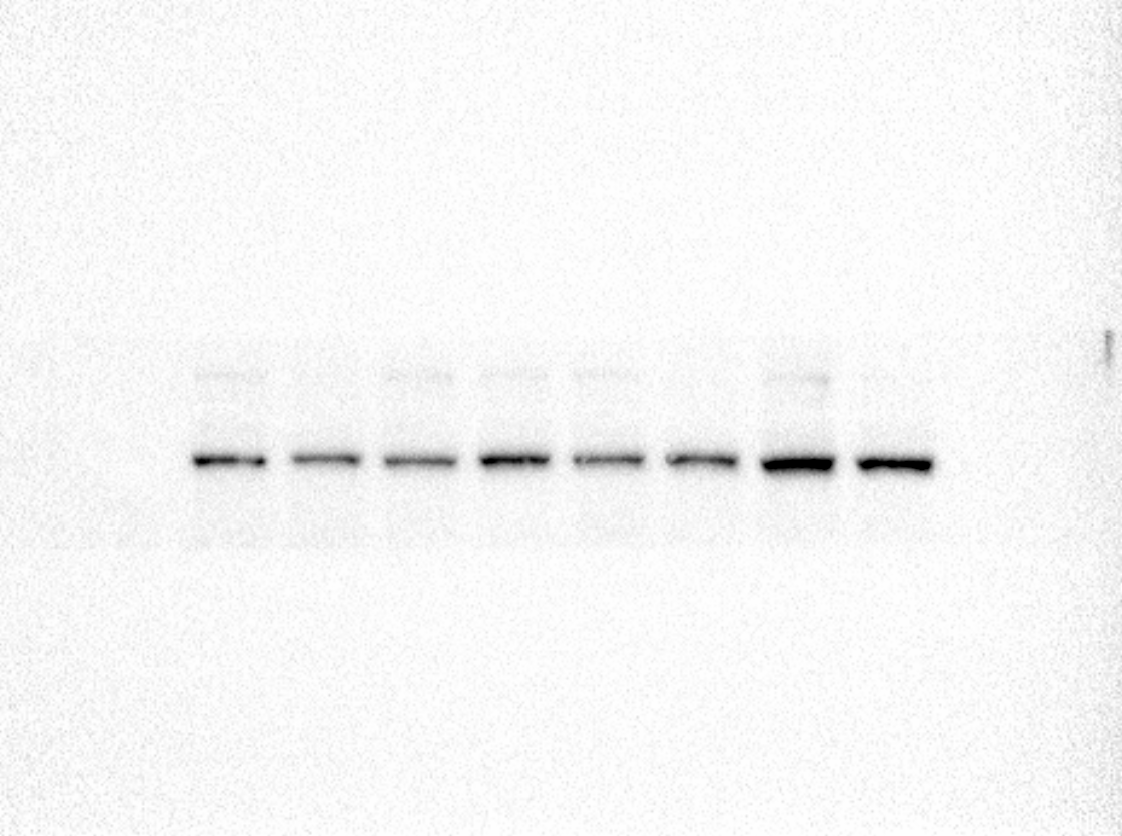

Supplement: Supplementary file 1 [file DataSheet3.ZIP › Raw data-2/Western Blot/in vivo/mTORC1/mTORC1(3,4).tif]

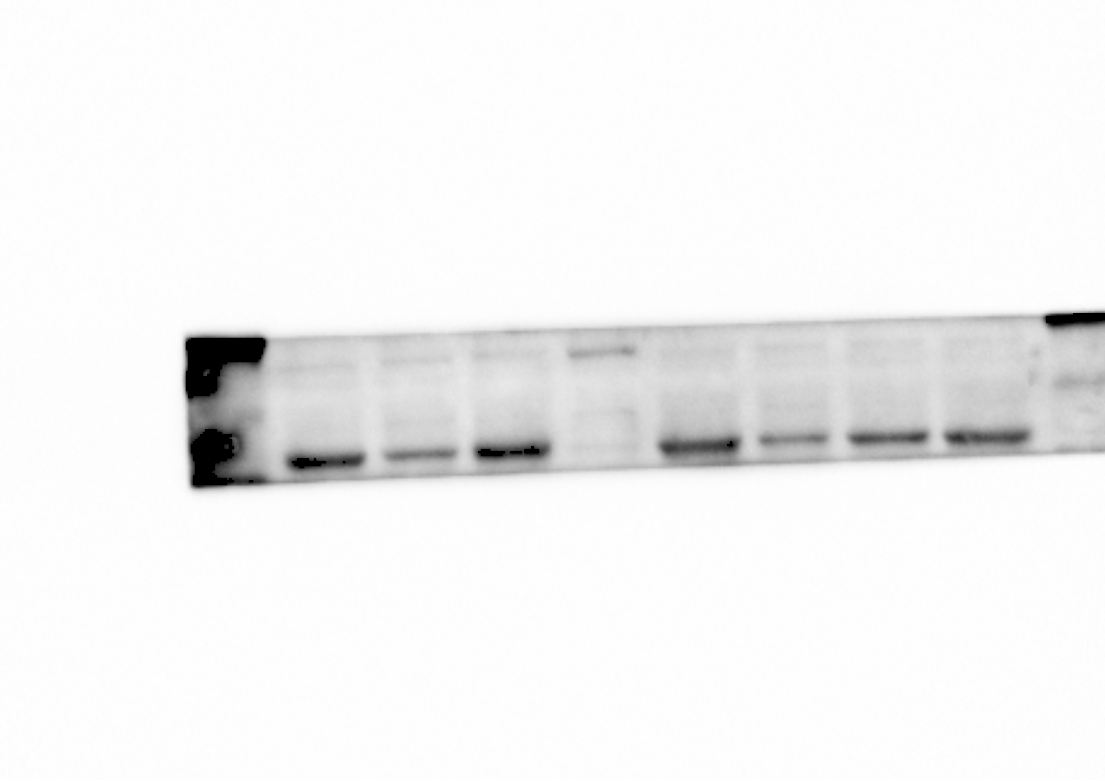

Supplement: Supplementary file 1 [file DataSheet3.ZIP › Raw data-2/Western Blot/in vivo/p-Akt/p-Akt(1,2).tif]

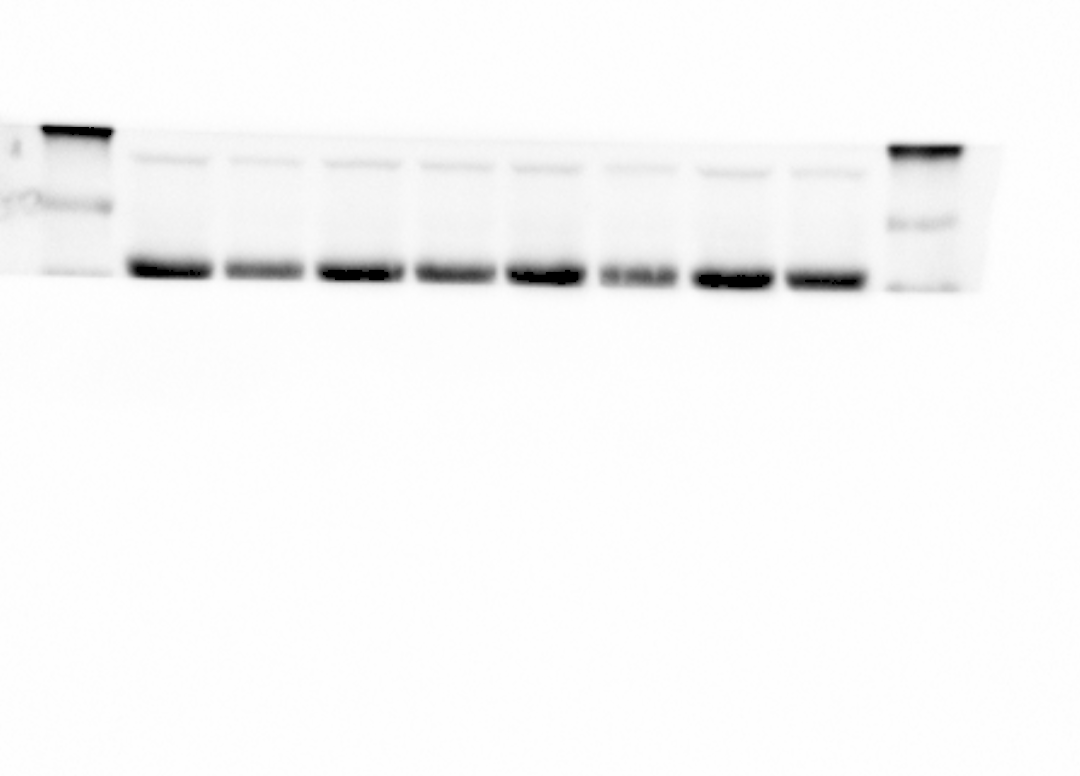

Supplement: Supplementary file 1 [file DataSheet3.ZIP › Raw data-2/Western Blot/in vivo/p-Akt/p-Akt(3.4).tif]

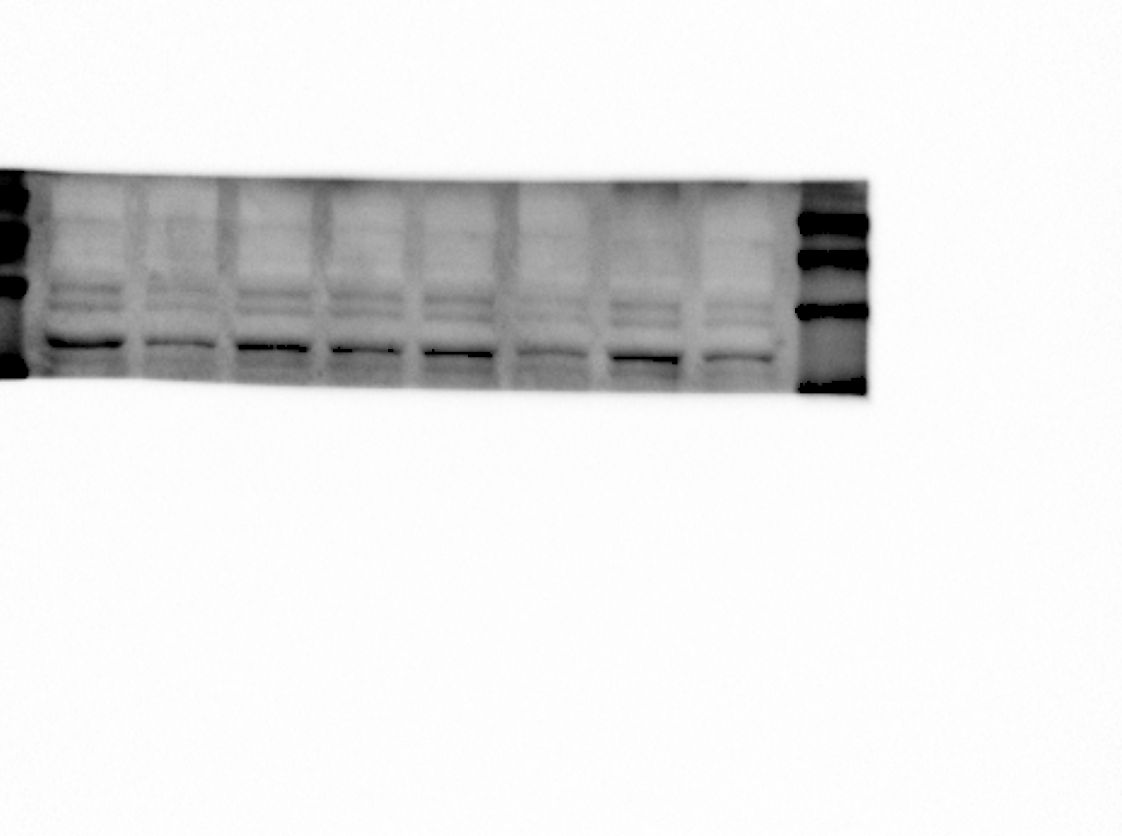

Supplement: Supplementary file 1 [file DataSheet3.ZIP › Raw data-2/Western Blot/in vivo/p-PI3K/p-PI3K(1.2).tif]

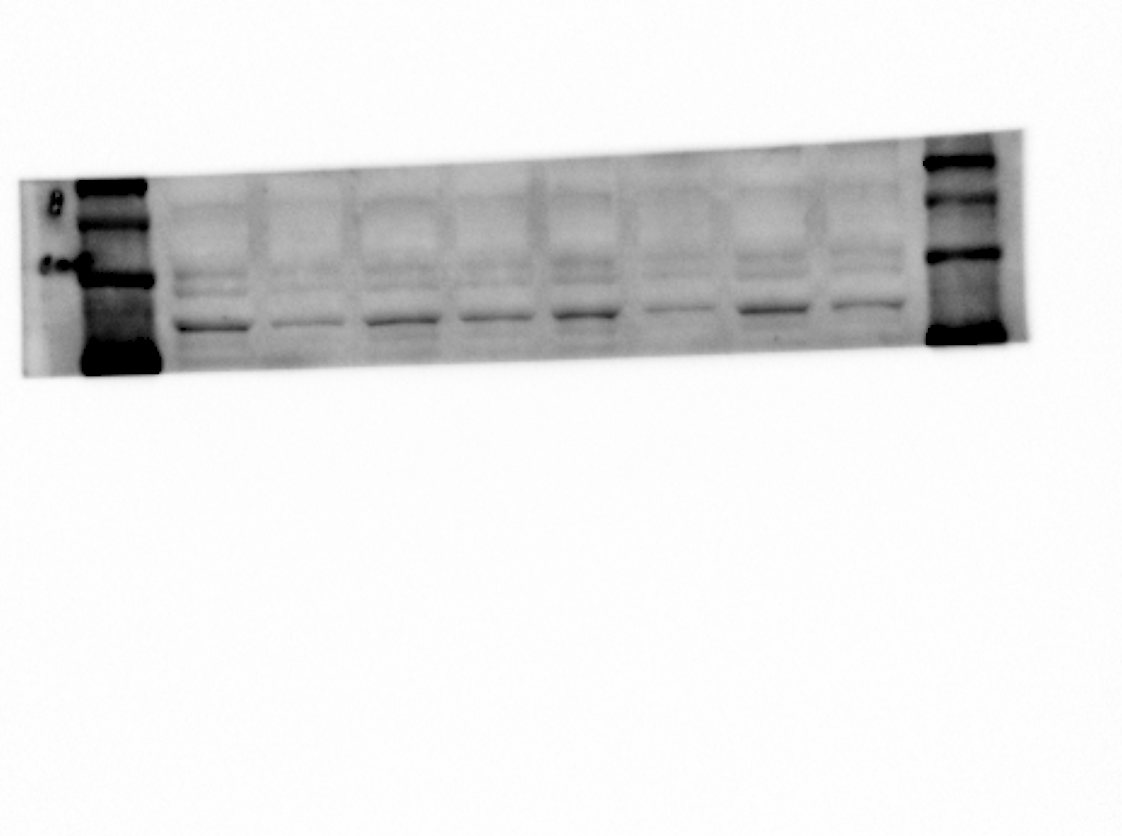

Supplement: Supplementary file 1 [file DataSheet3.ZIP › Raw data-2/Western Blot/in vivo/p-PI3K/p-PI3K(3,4).tif]

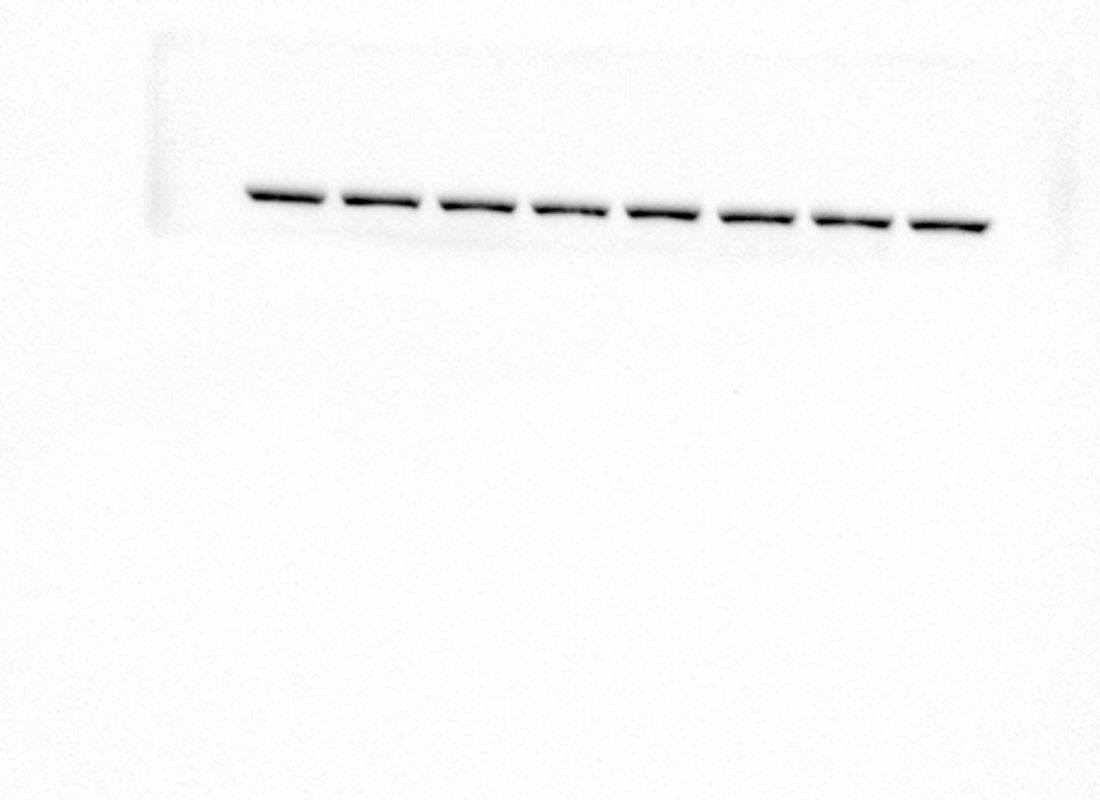

Supplement: Supplementary file 1 [file DataSheet3.ZIP › Raw data-2/Western Blot/in vivo/PI3K/PI3K(1,2).tif]

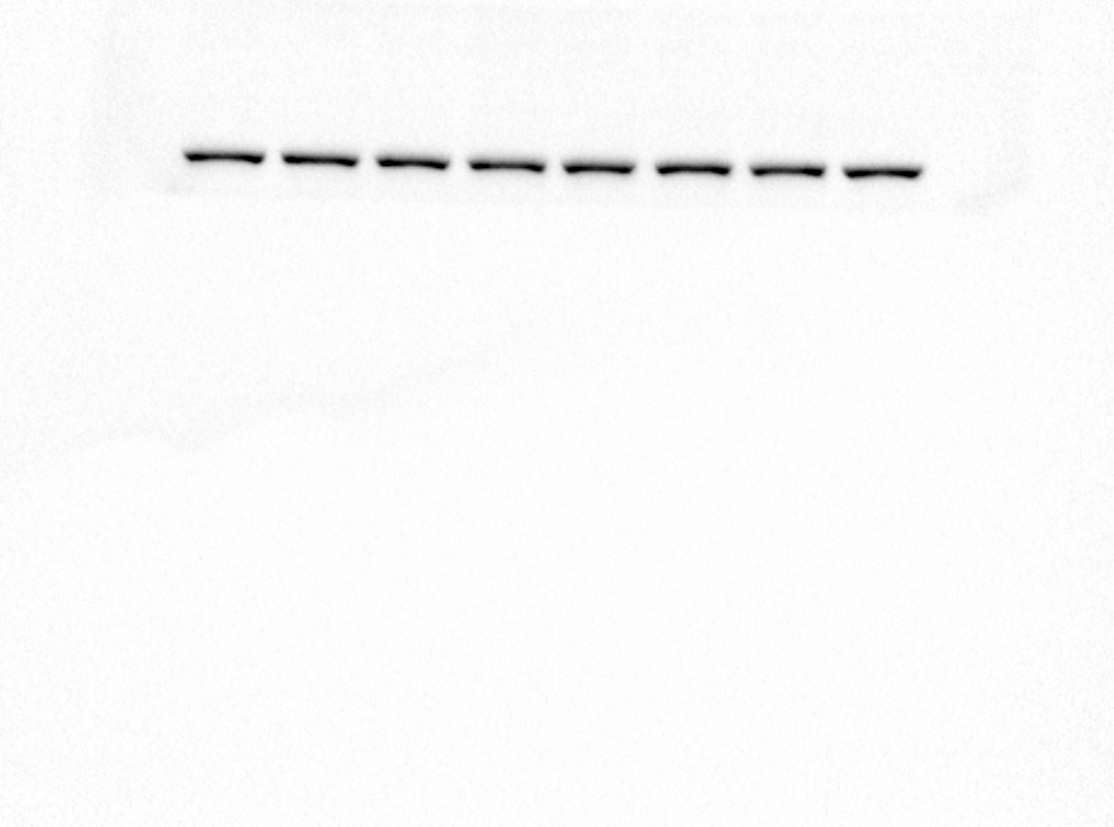

Supplement: Supplementary file 1 [file DataSheet3.ZIP › Raw data-2/Western Blot/in vivo/PI3K/PI3K(3,4).tif]

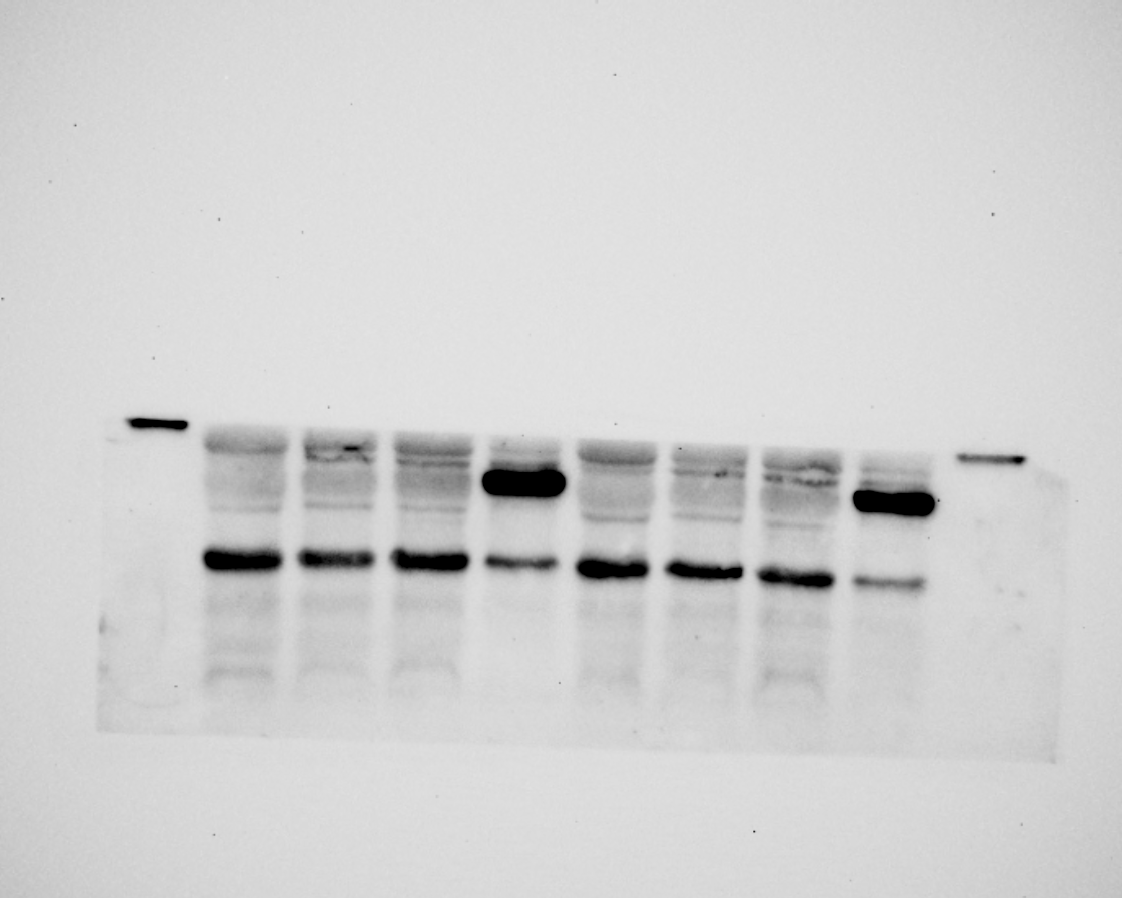

Supplement: Supplementary file 1 [file DataSheet3.ZIP › Raw data-2/Western Blot/in vivo/VEGF-A/VEGF-A(1,2).tif]

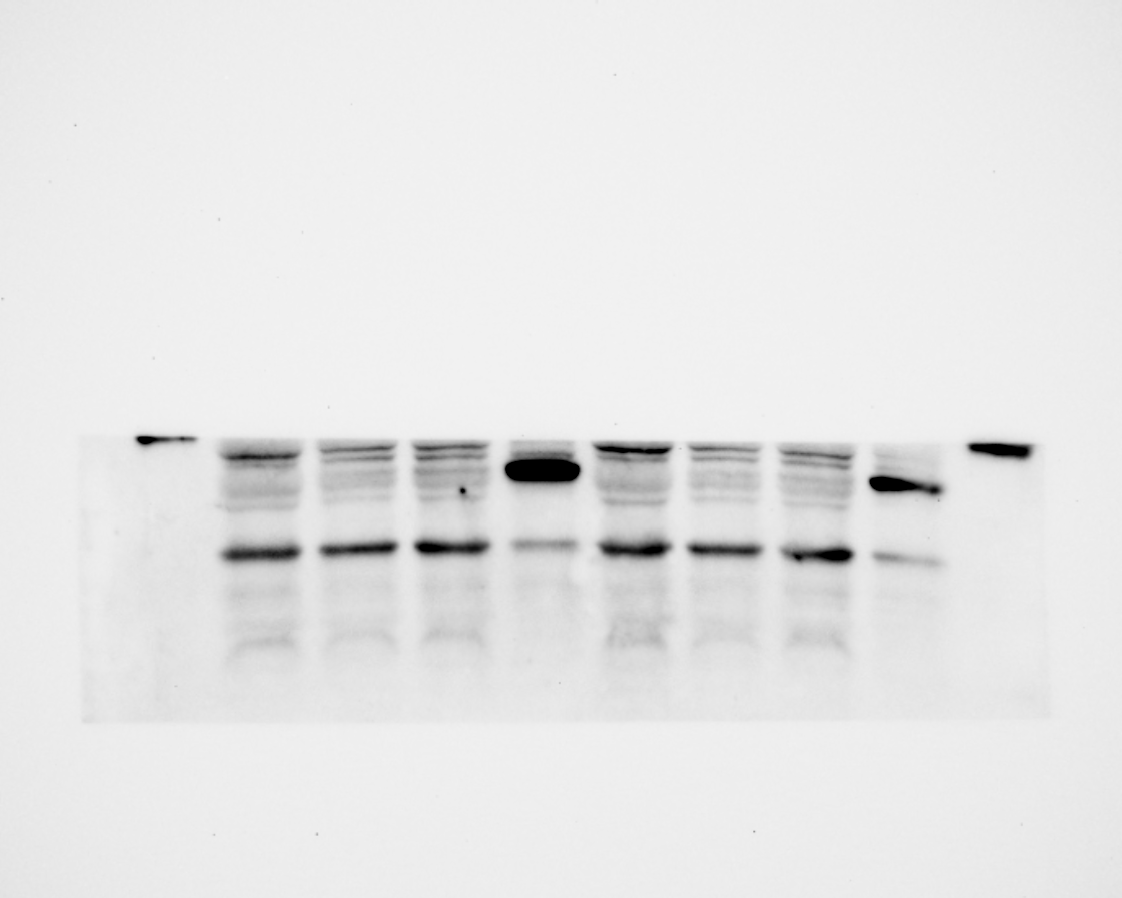

Supplement: Supplementary file 1 [file DataSheet3.ZIP › Raw data-2/Western Blot/in vivo/VEGF-A/VEGF-A(3,4).tif]

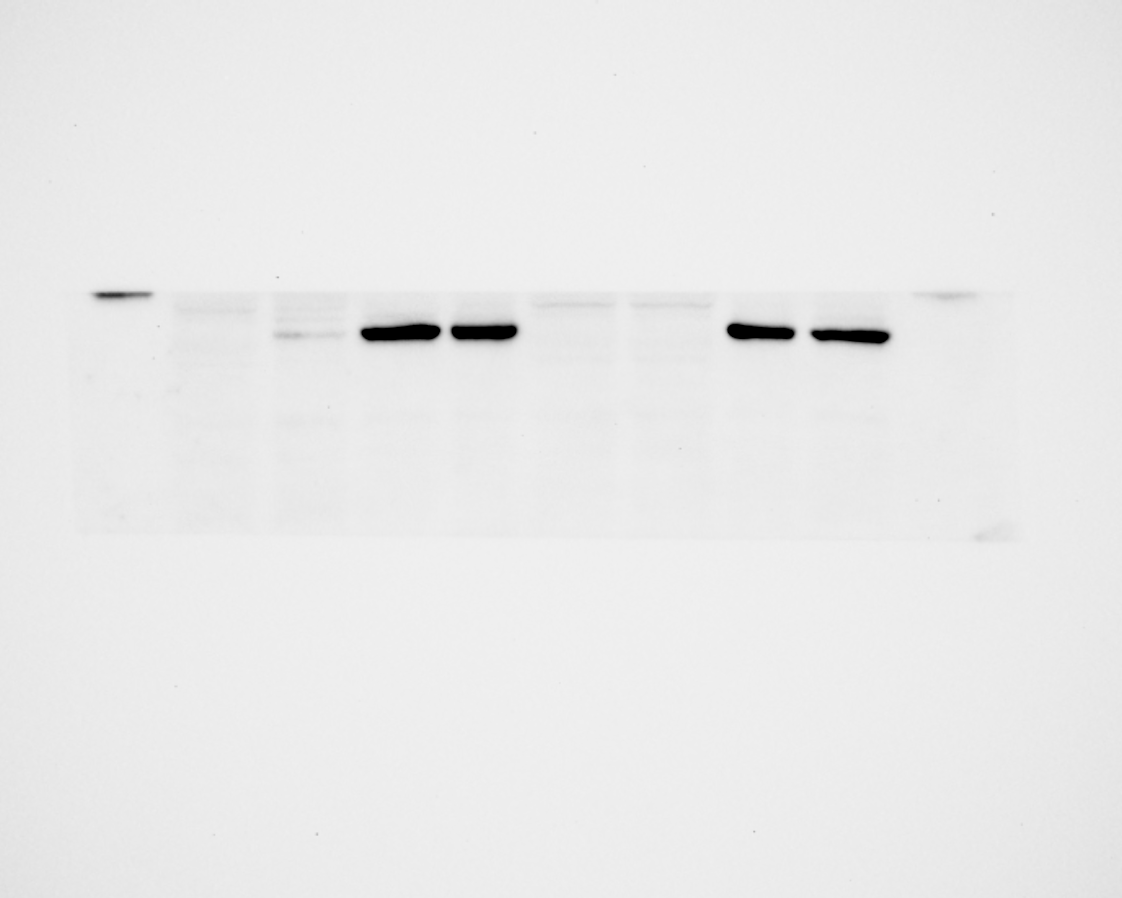

Supplement: Supplementary file 1 [file DataSheet3.ZIP › Raw data-2/Western Blot/in vivo/VEGF-A/VEGF-A(5,6).tif]

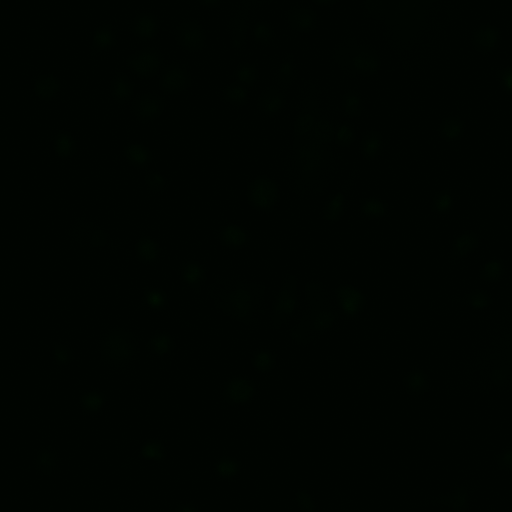

Supplement: Supplementary file 3 [file DataSheet4.ZIP › Raw data-3/Immunofluorescence (IF) Analysis/in vitro/Control/Control-CD206.tif]

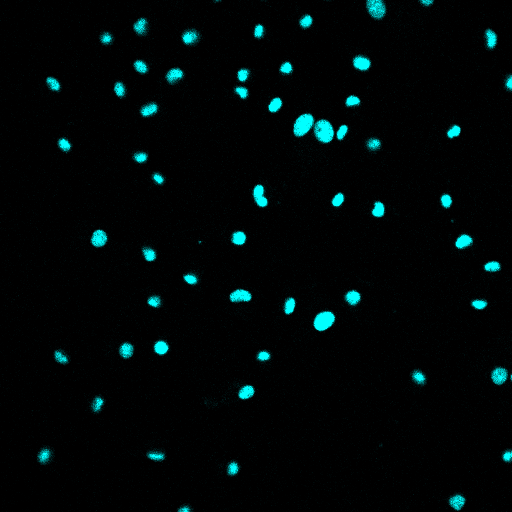

Supplement: Supplementary file 3 [file DataSheet4.ZIP › Raw data-3/Immunofluorescence (IF) Analysis/in vitro/Control/Control-DAPI.tif]

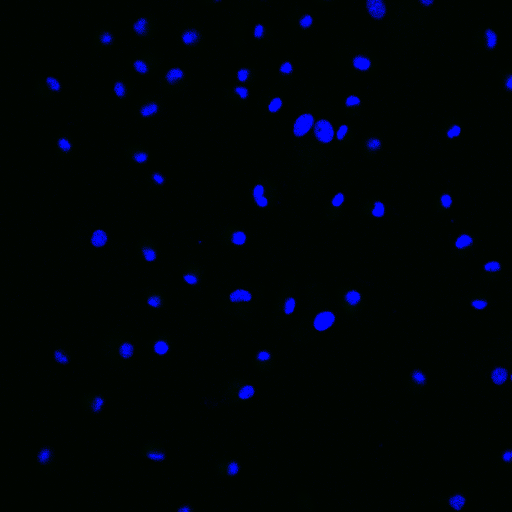

Supplement: Supplementary file 3 [file DataSheet4.ZIP › Raw data-3/Immunofluorescence (IF) Analysis/in vitro/Control/Control-Merge.tif]

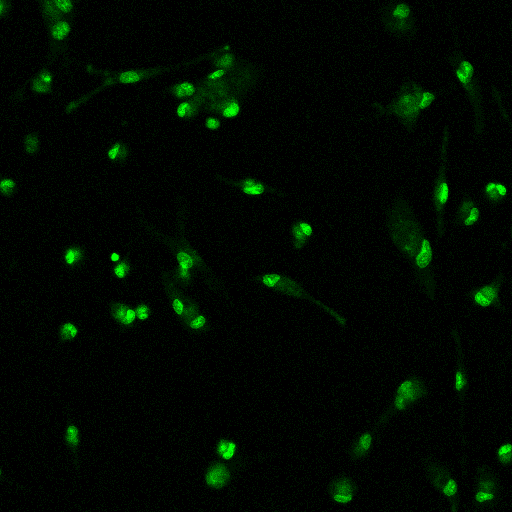

Supplement: Supplementary file 3 [file DataSheet4.ZIP › Raw data-3/Immunofluorescence (IF) Analysis/in vitro/LY29+STDP/LY29+STDP-CD206.tif]

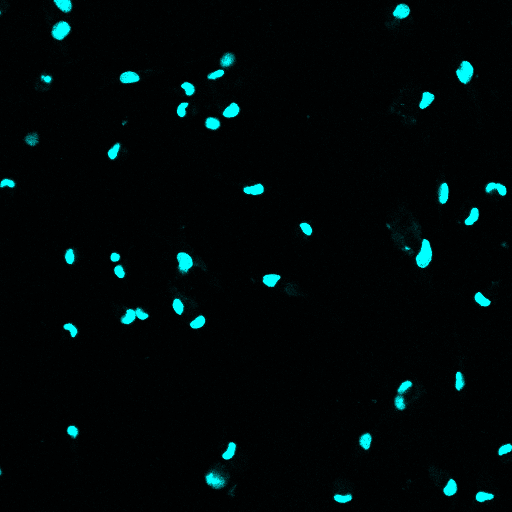

Supplement: Supplementary file 3 [file DataSheet4.ZIP › Raw data-3/Immunofluorescence (IF) Analysis/in vitro/LY29+STDP/LY29+STDP-DAPI.tif]

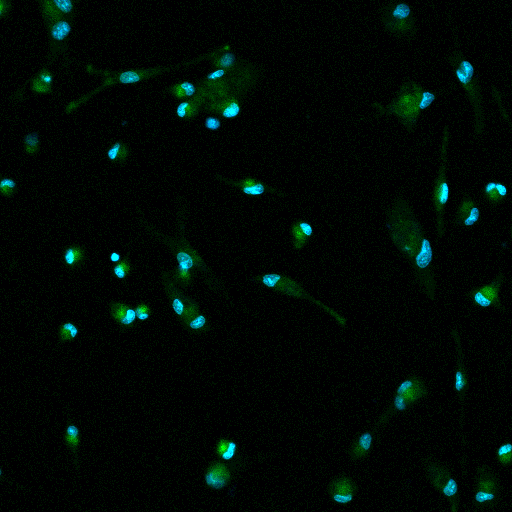

Supplement: Supplementary file 3 [file DataSheet4.ZIP › Raw data-3/Immunofluorescence (IF) Analysis/in vitro/LY29+STDP/LY29+STDP-Merge.tif]

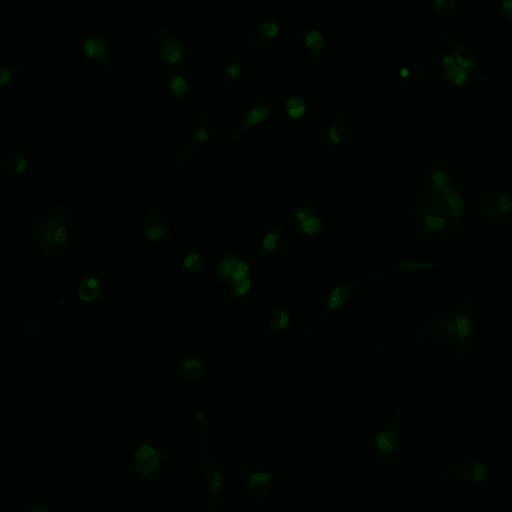

Supplement: Supplementary file 3 [file DataSheet4.ZIP › Raw data-3/Immunofluorescence (IF) Analysis/in vitro/LY29/LY29-CD206.tif]

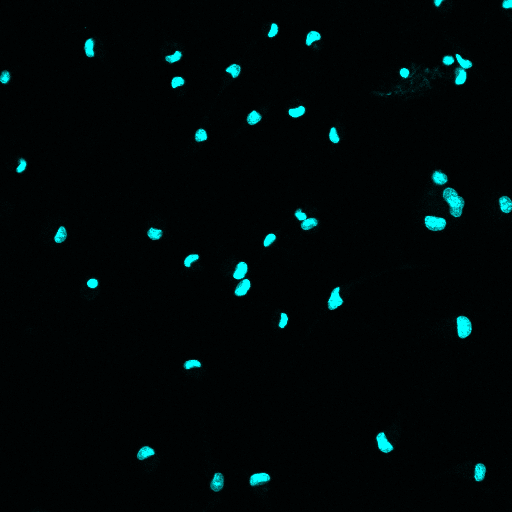

Supplement: Supplementary file 3 [file DataSheet4.ZIP › Raw data-3/Immunofluorescence (IF) Analysis/in vitro/LY29/LY29-DAPI.tif]

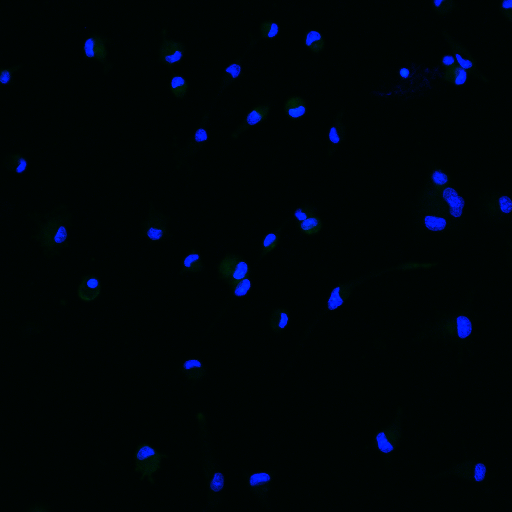

Supplement: Supplementary file 3 [file DataSheet4.ZIP › Raw data-3/Immunofluorescence (IF) Analysis/in vitro/LY29/LY29-Merge.tif]

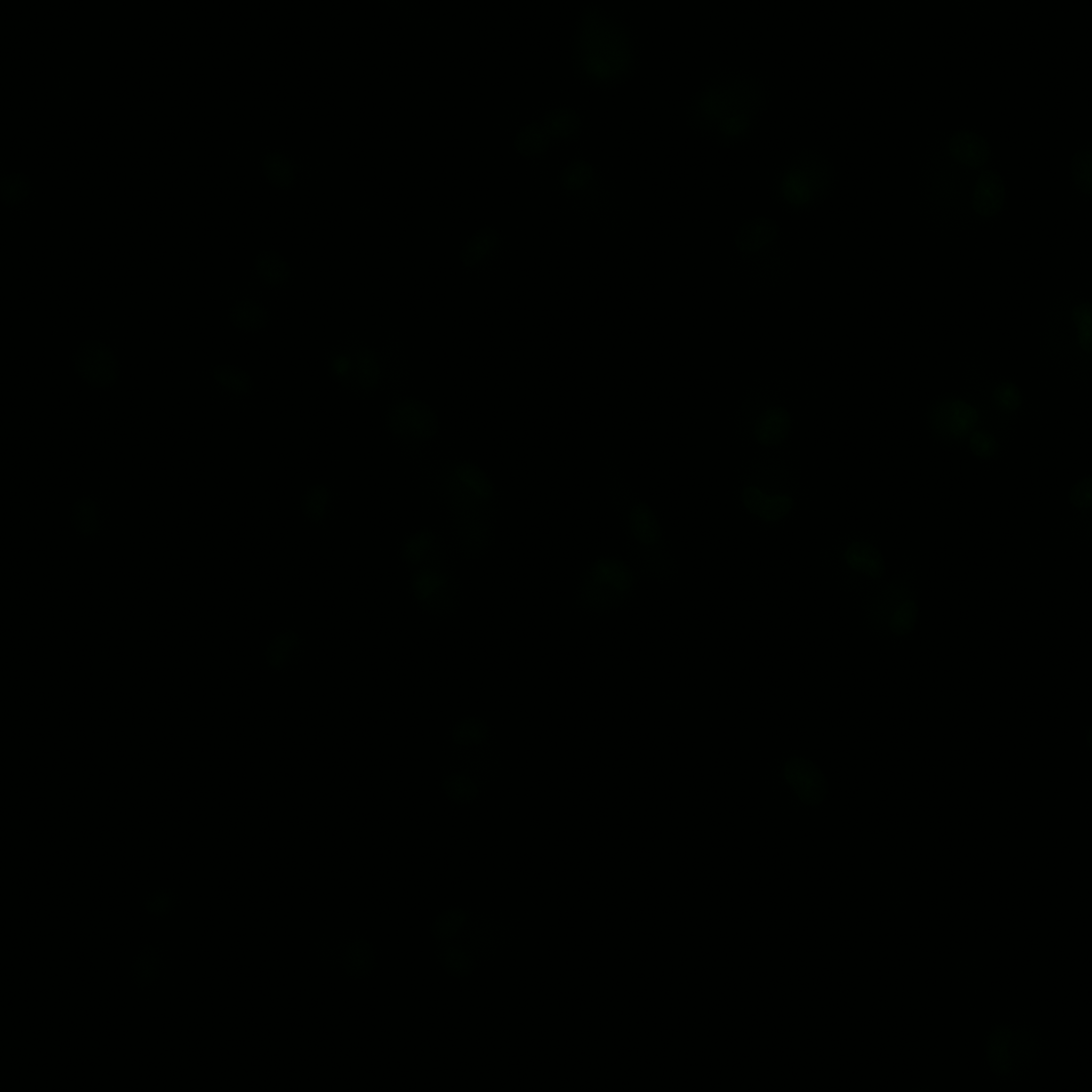

Supplement: Supplementary file 3 [file DataSheet4.ZIP › Raw data-3/Immunofluorescence (IF) Analysis/in vitro/Model/Model-CD206.tif]

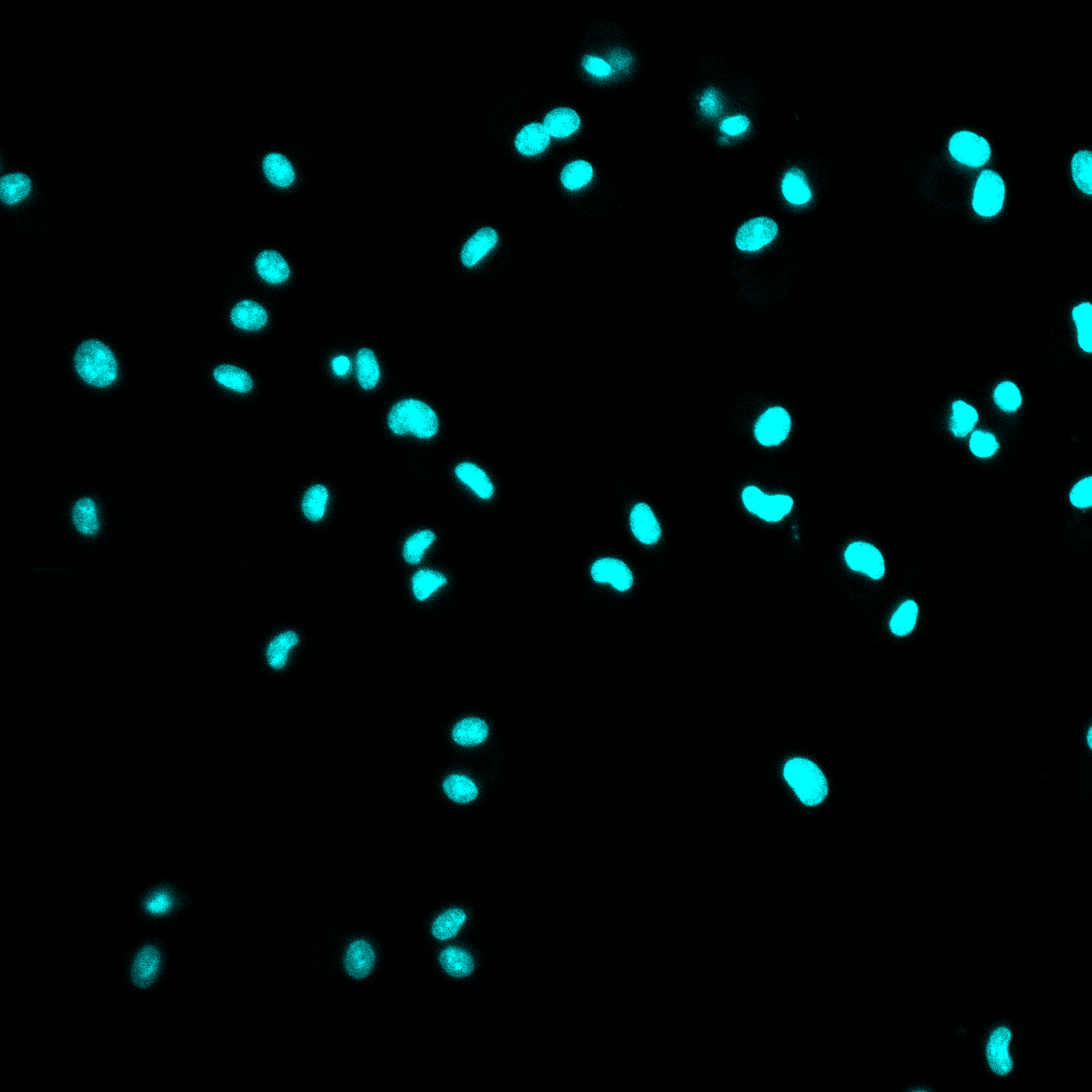

Supplement: Supplementary file 3 [file DataSheet4.ZIP › Raw data-3/Immunofluorescence (IF) Analysis/in vitro/Model/Model-DAPI.tif]

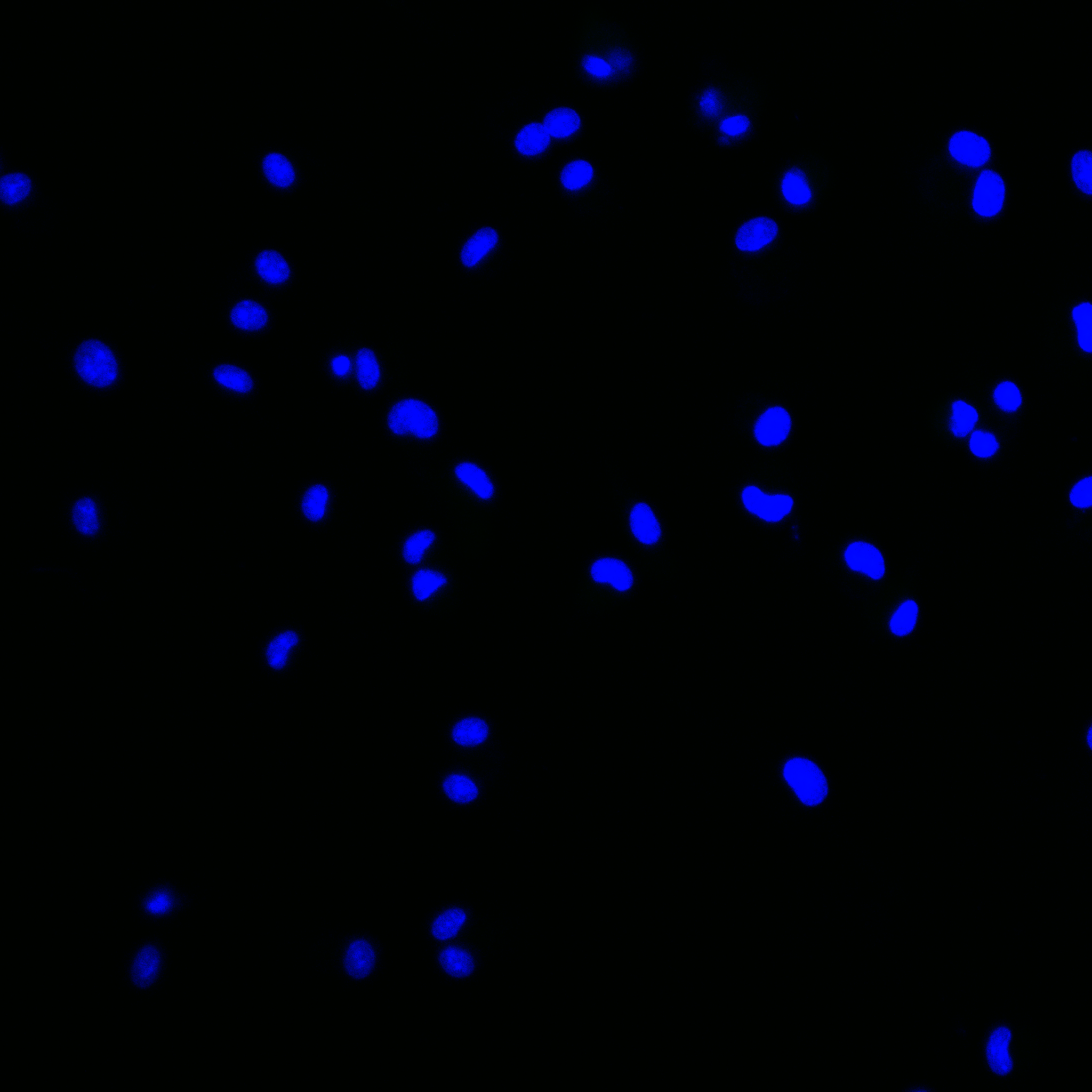

Supplement: Supplementary file 3 [file DataSheet4.ZIP › Raw data-3/Immunofluorescence (IF) Analysis/in vitro/Model/Model-Merge.tif]

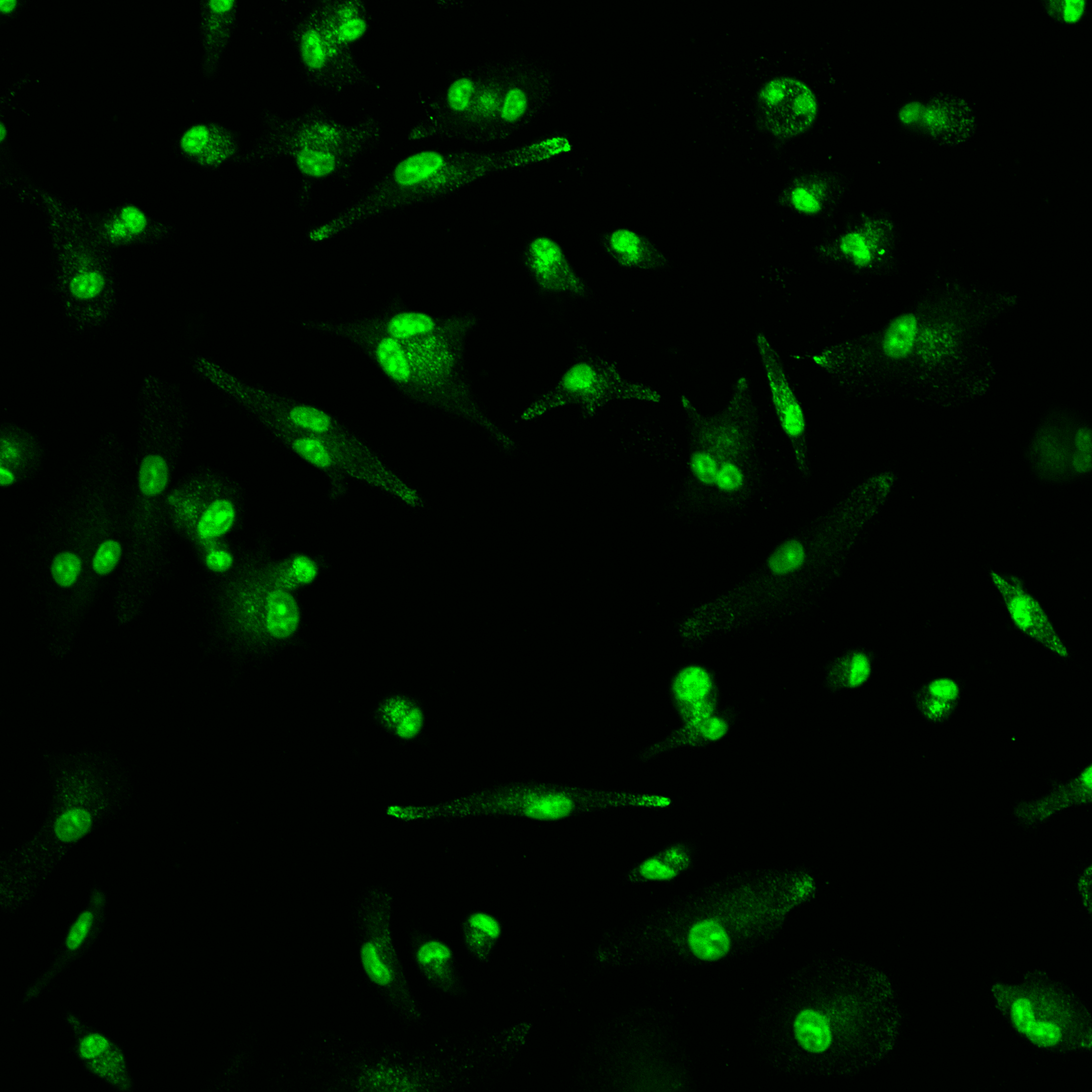

Supplement: Supplementary file 3 [file DataSheet4.ZIP › Raw data-3/Immunofluorescence (IF) Analysis/in vitro/STDP/STDP-CD206.tif]

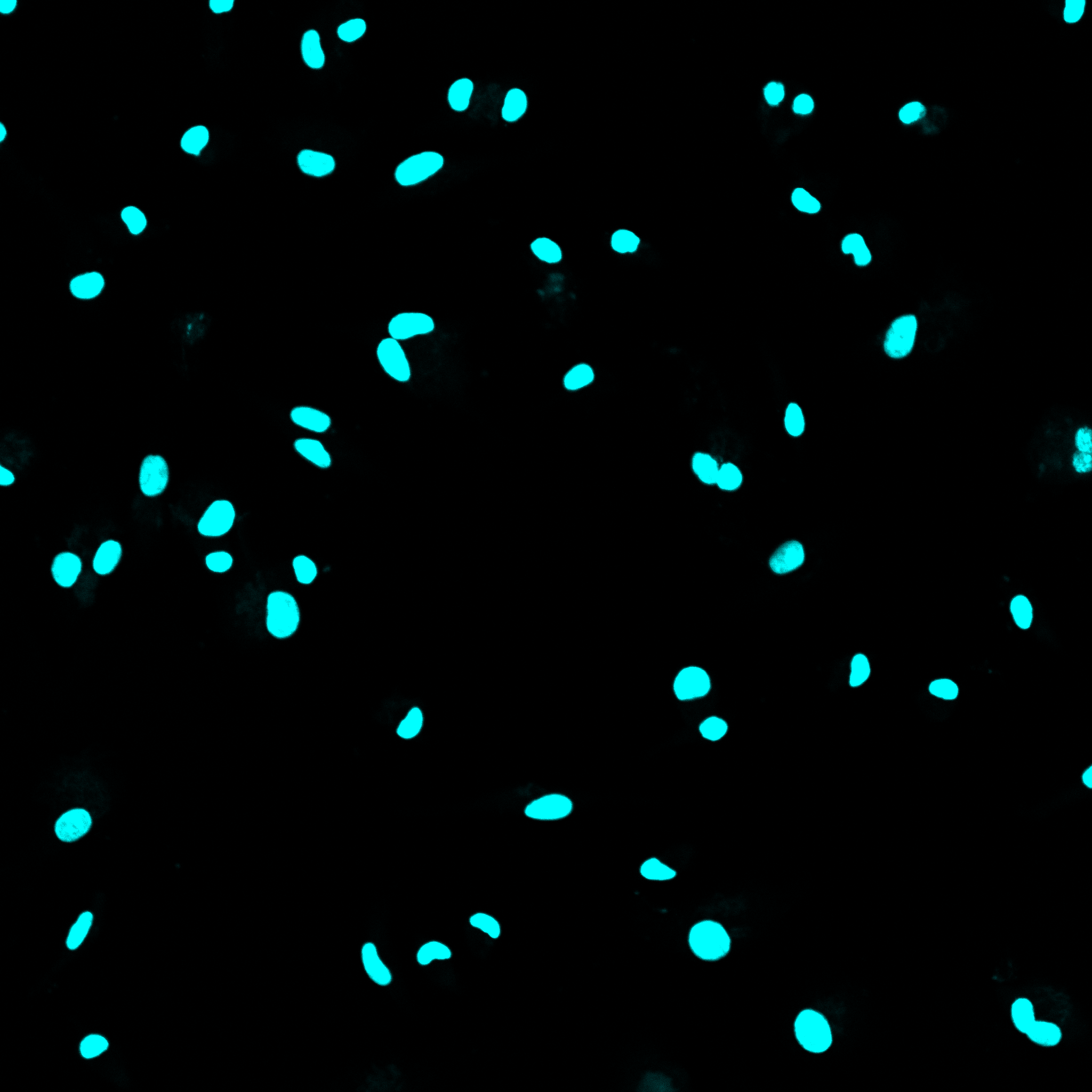

Supplement: Supplementary file 3 [file DataSheet4.ZIP › Raw data-3/Immunofluorescence (IF) Analysis/in vitro/STDP/STDP-DAPI.tif]

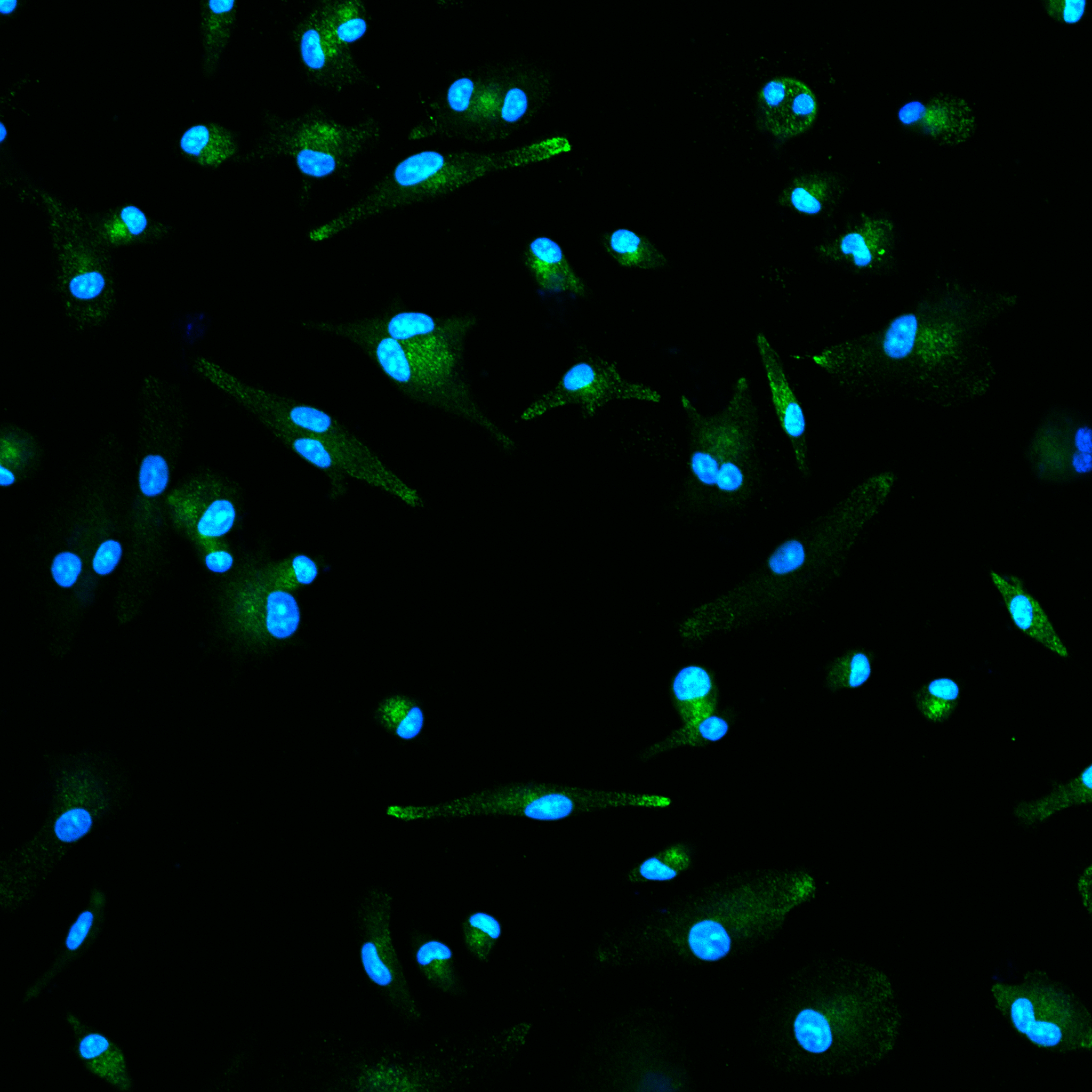

Supplement: Supplementary file 3 [file DataSheet4.ZIP › Raw data-3/Immunofluorescence (IF) Analysis/in vitro/STDP/STDP-Merge.tif]

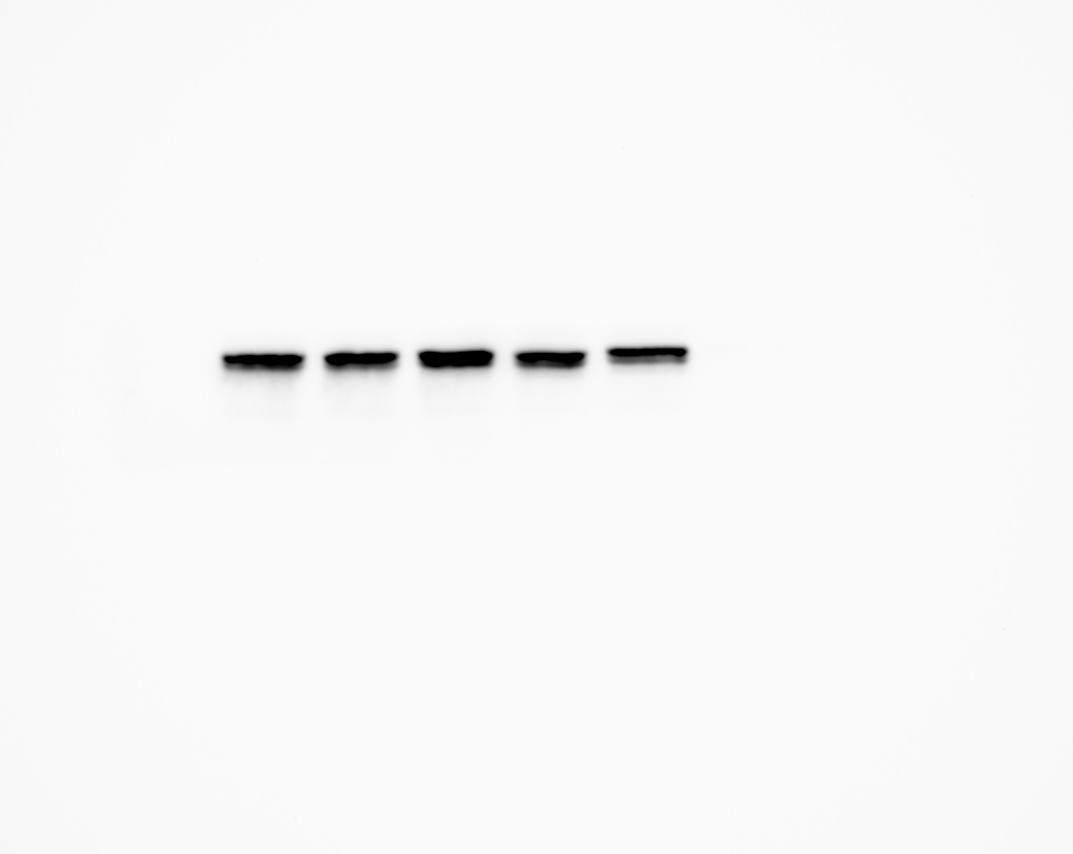

Supplement: Supplementary file 3 [file DataSheet4.ZIP › Raw data-3/Western Blot/in vitro/Akt/Akt(1).tif]

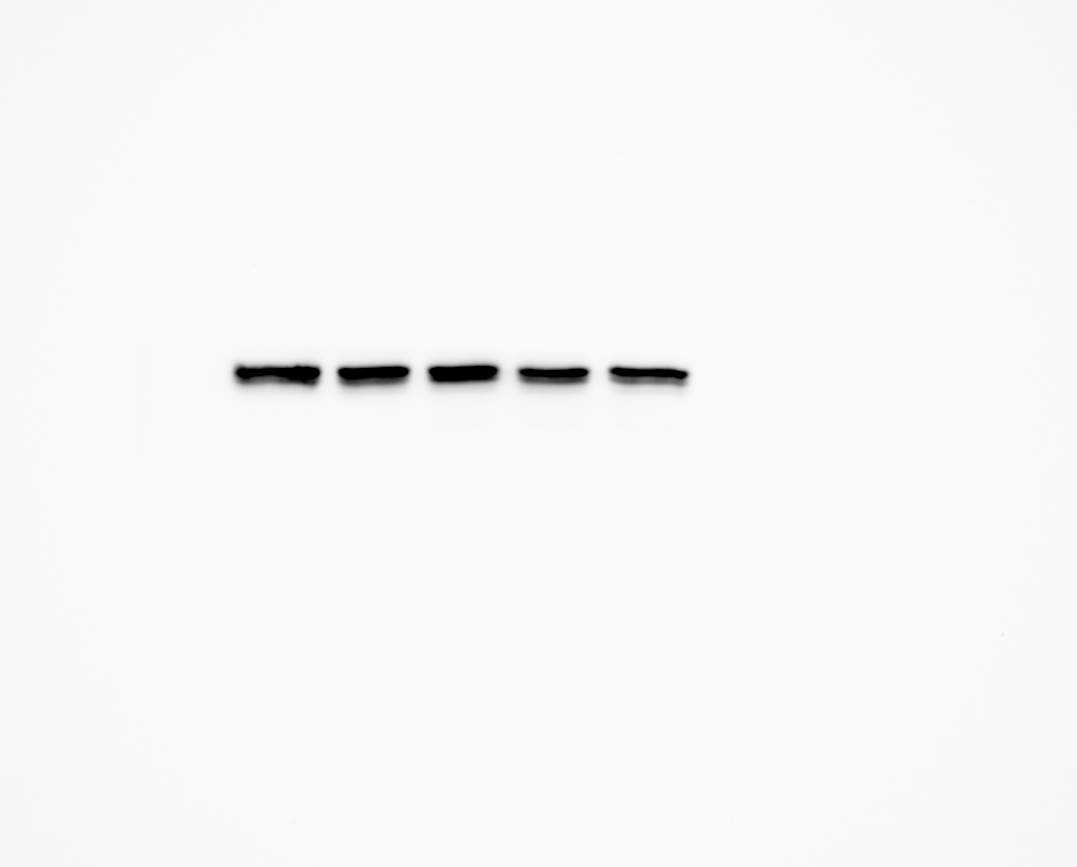

Supplement: Supplementary file 3 [file DataSheet4.ZIP › Raw data-3/Western Blot/in vitro/Akt/Akt(2).tif]

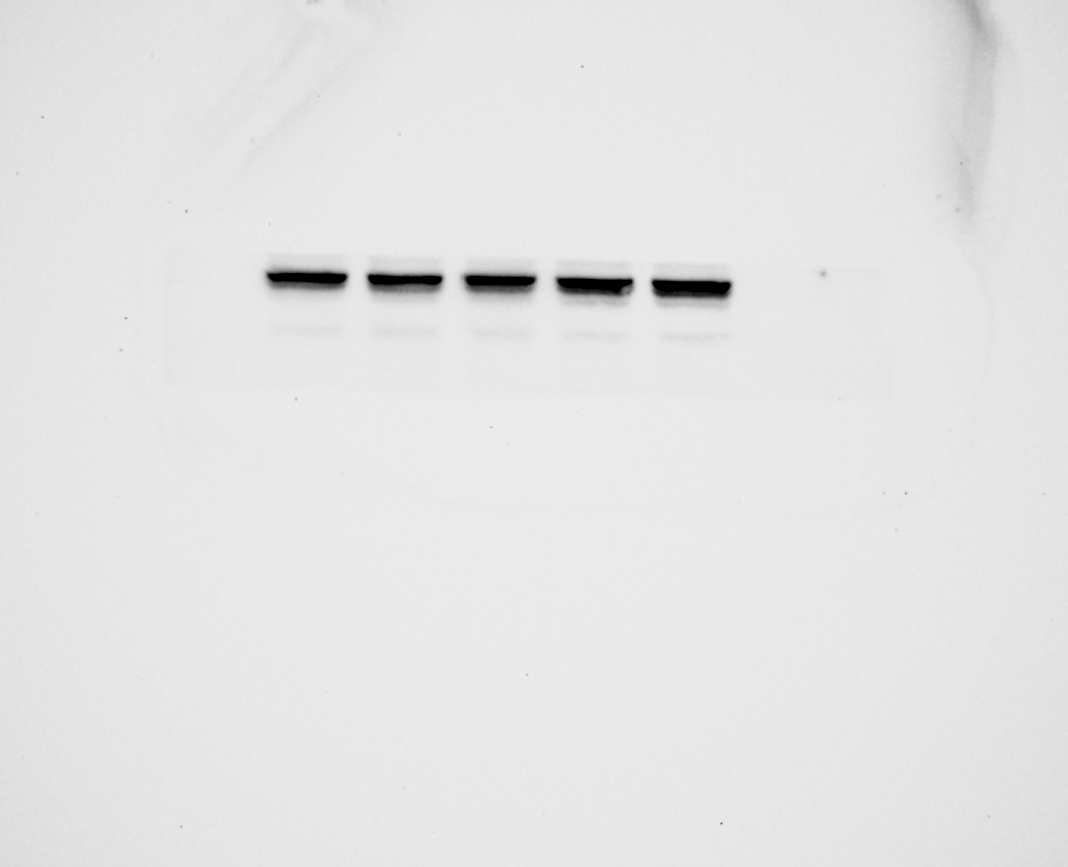

Supplement: Supplementary file 3 [file DataSheet4.ZIP › Raw data-3/Western Blot/in vitro/Akt/Akt(3).tif]

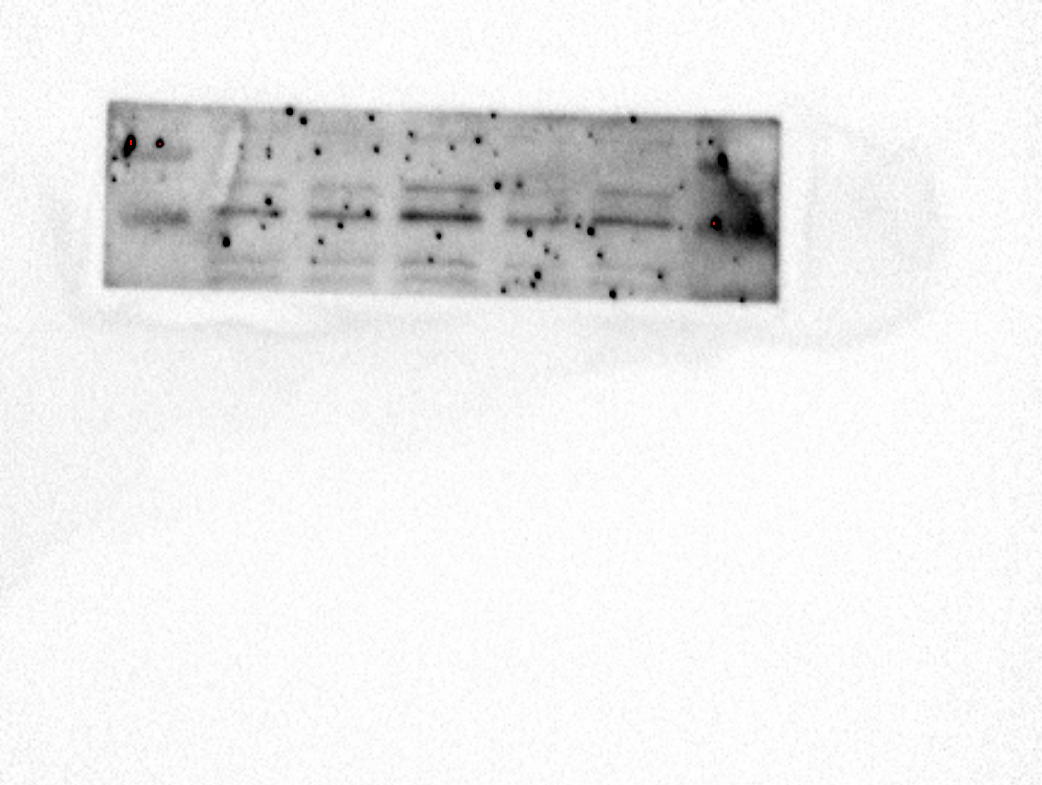

Supplement: Supplementary file 3 [file DataSheet4.ZIP › Raw data-3/Western Blot/in vitro/Arg-1/Arg-1(1).tif]

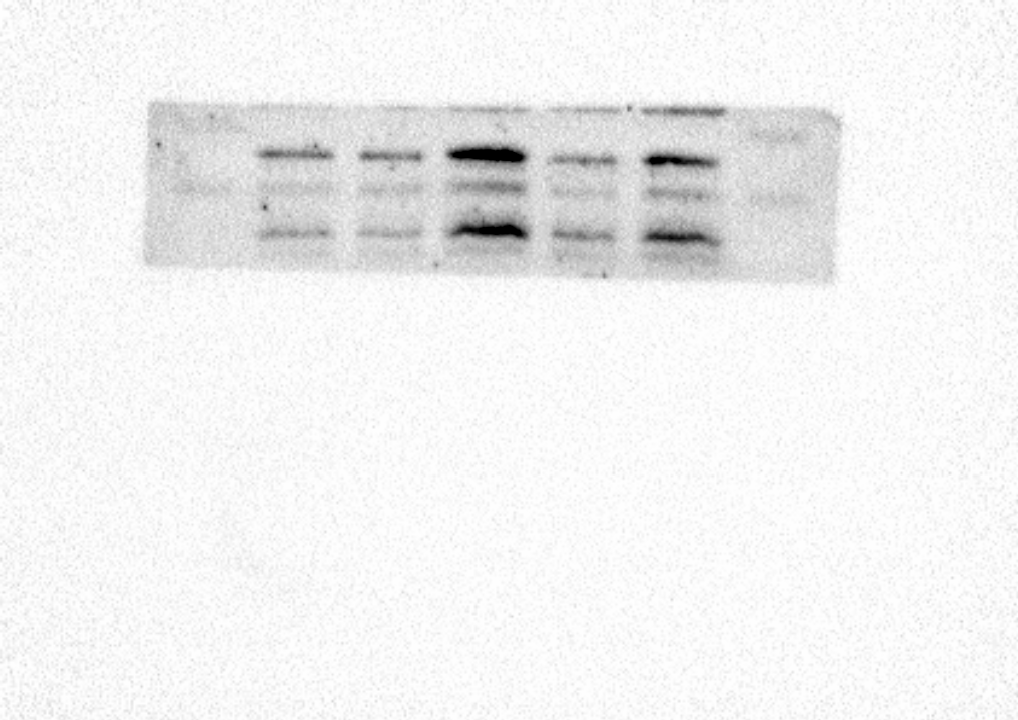

Supplement: Supplementary file 3 [file DataSheet4.ZIP › Raw data-3/Western Blot/in vitro/Arg-1/Arg-1(2).tif]

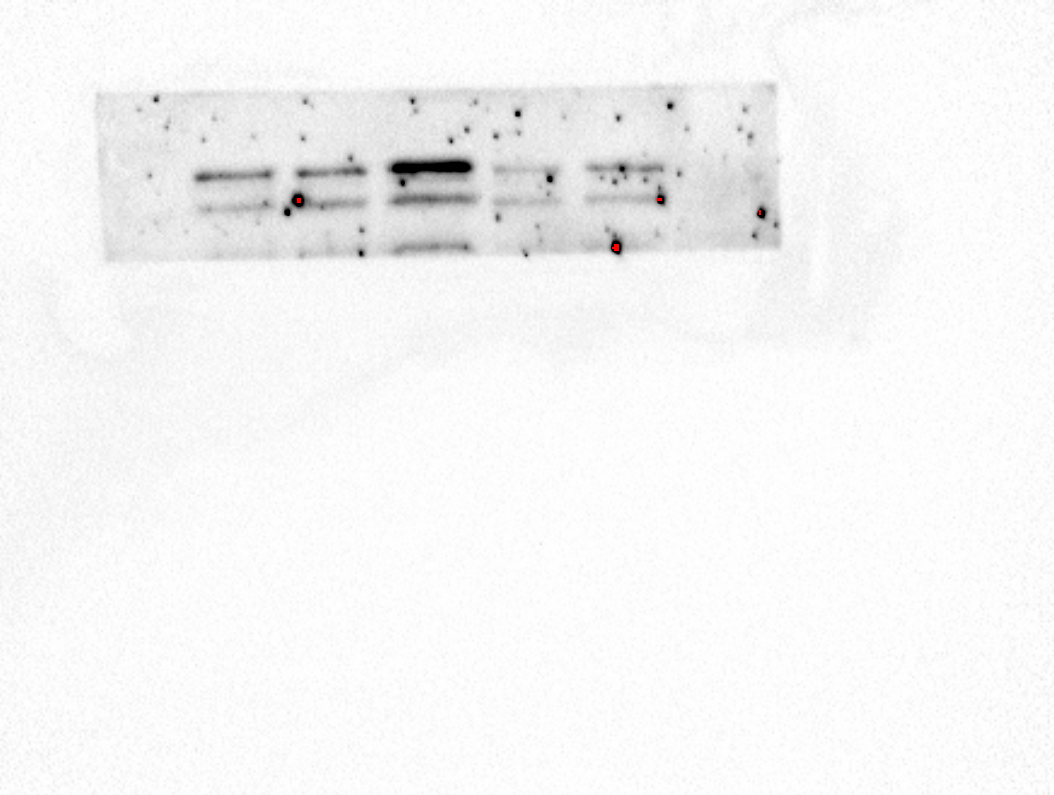

Supplement: Supplementary file 3 [file DataSheet4.ZIP › Raw data-3/Western Blot/in vitro/Arg-1/Arg-1(3).tif]

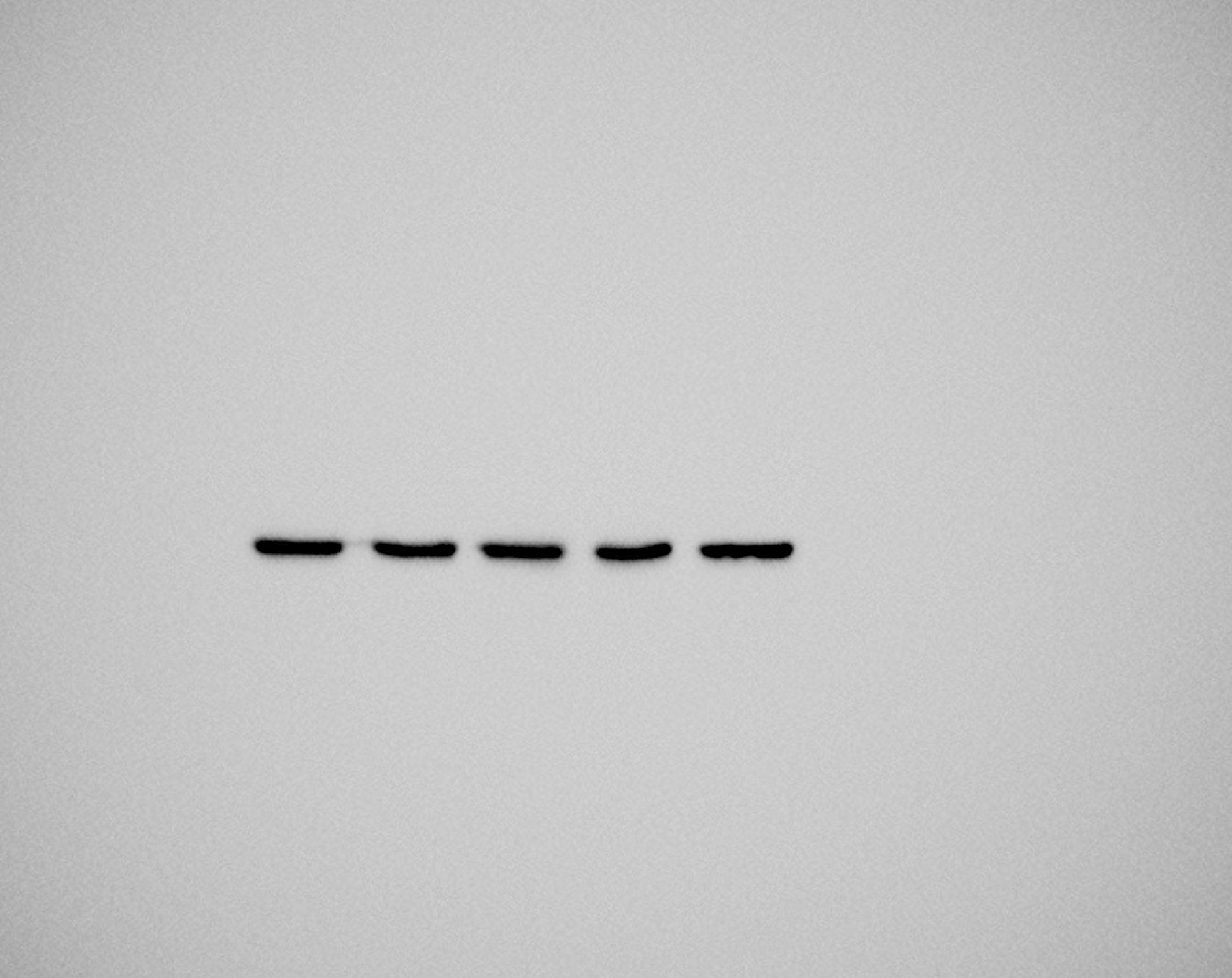

Supplement: Supplementary file 3 [file DataSheet4.ZIP › Raw data-3/Western Blot/in vitro/GAPDH/GAPDH(1).tif]

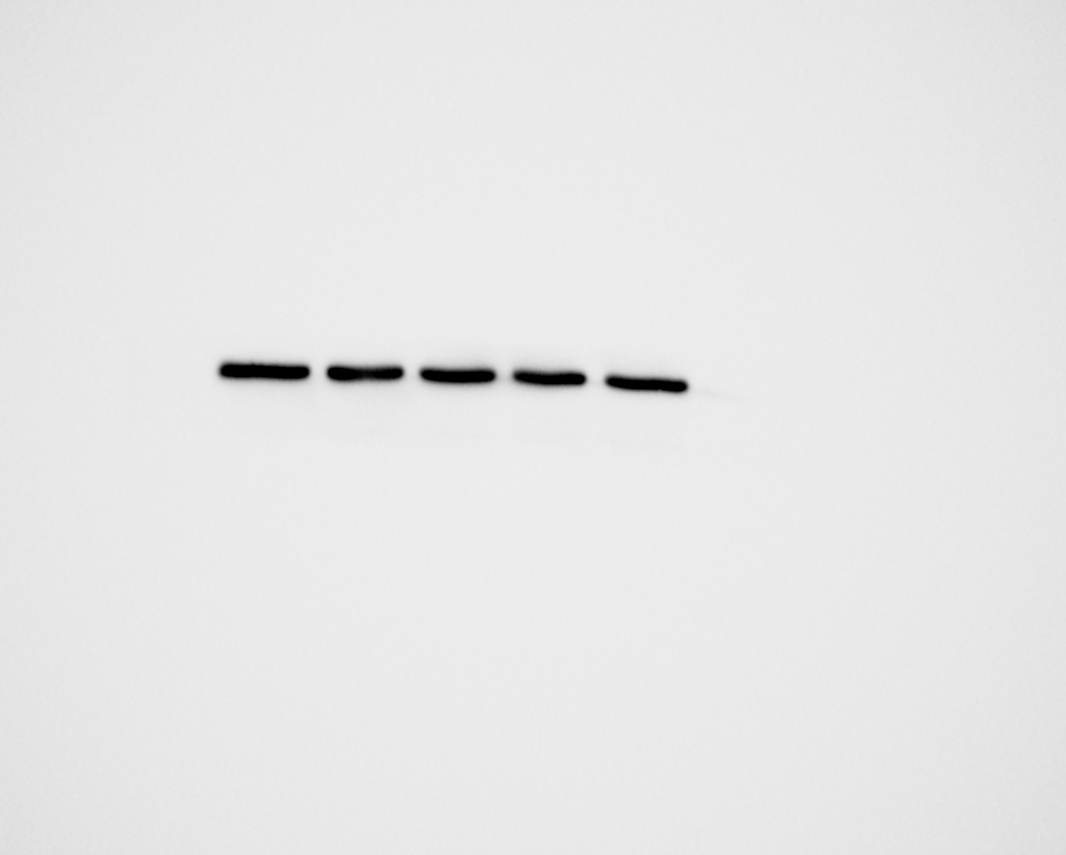

Supplement: Supplementary file 3 [file DataSheet4.ZIP › Raw data-3/Western Blot/in vitro/GAPDH/GAPDH(2).tif]

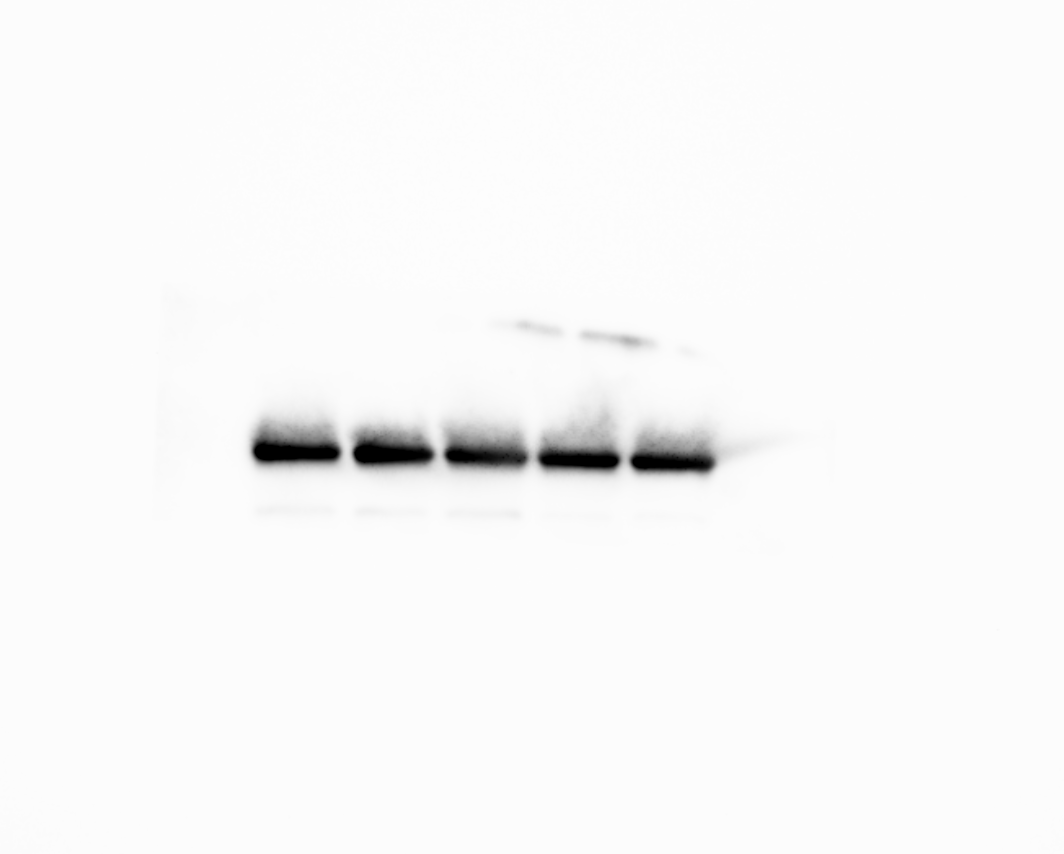

Supplement: Supplementary file 3 [file DataSheet4.ZIP › Raw data-3/Western Blot/in vitro/GAPDH/GAPDH(3).tif]

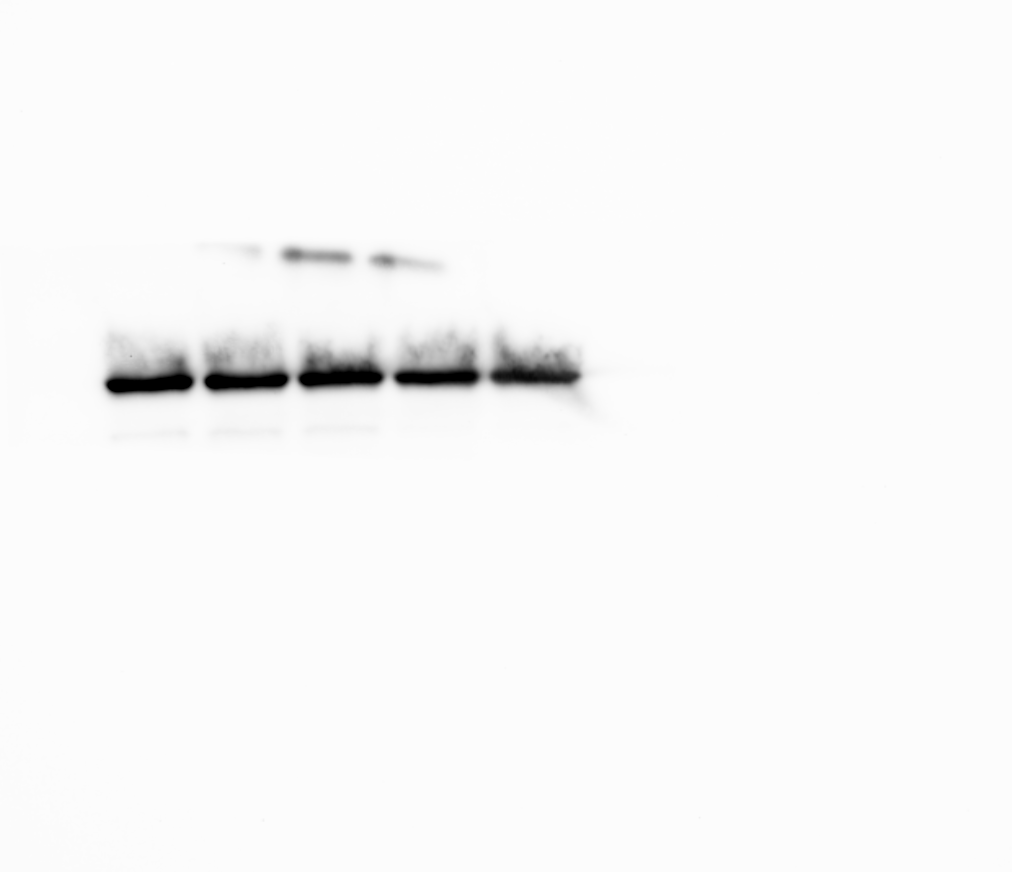

Supplement: Supplementary file 3 [file DataSheet4.ZIP › Raw data-3/Western Blot/in vitro/GAPDH/GAPDH(4).tif]

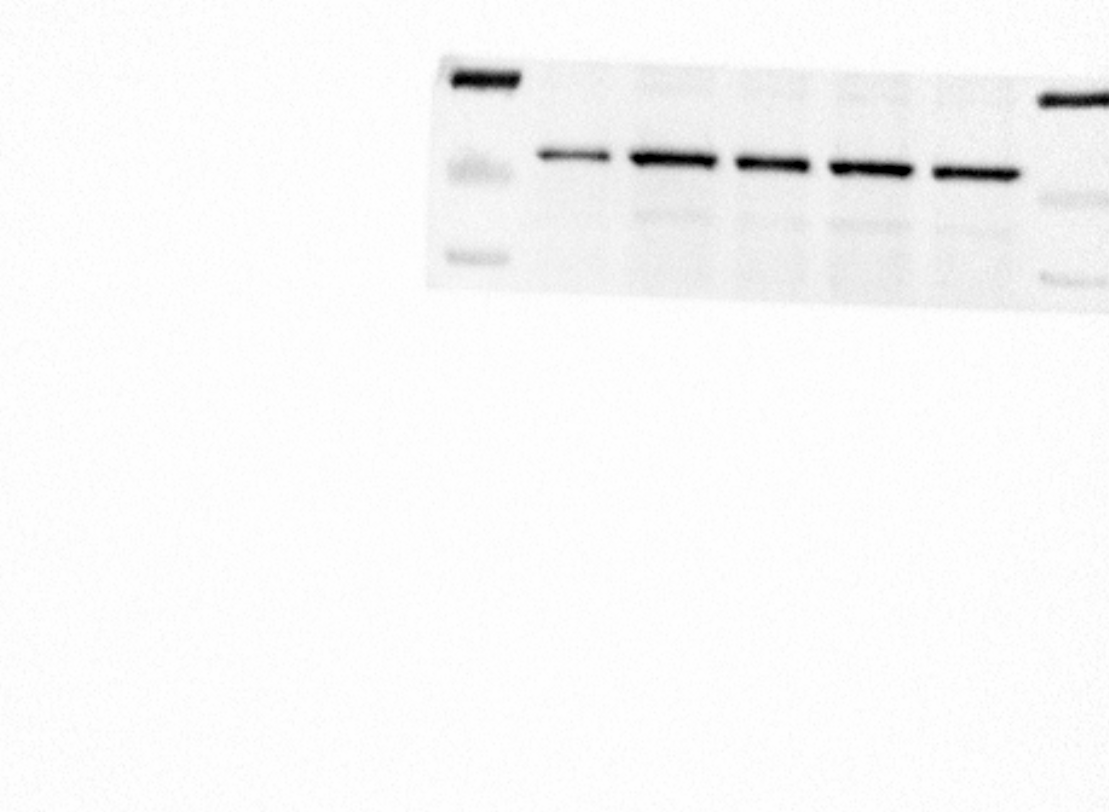

Supplement: Supplementary file 3 [file DataSheet4.ZIP › Raw data-3/Western Blot/in vitro/mTORC1/mTORC1(1).tif]

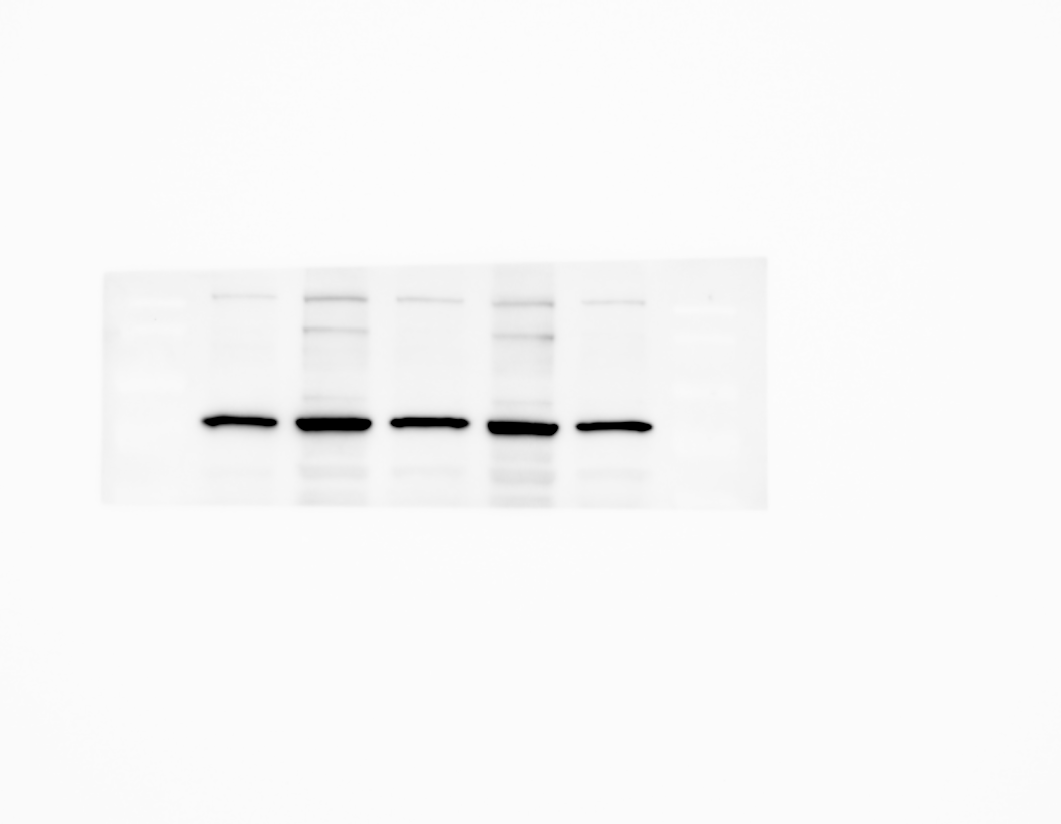

Supplement: Supplementary file 3 [file DataSheet4.ZIP › Raw data-3/Western Blot/in vitro/mTORC1/mTORC1(2).tif]

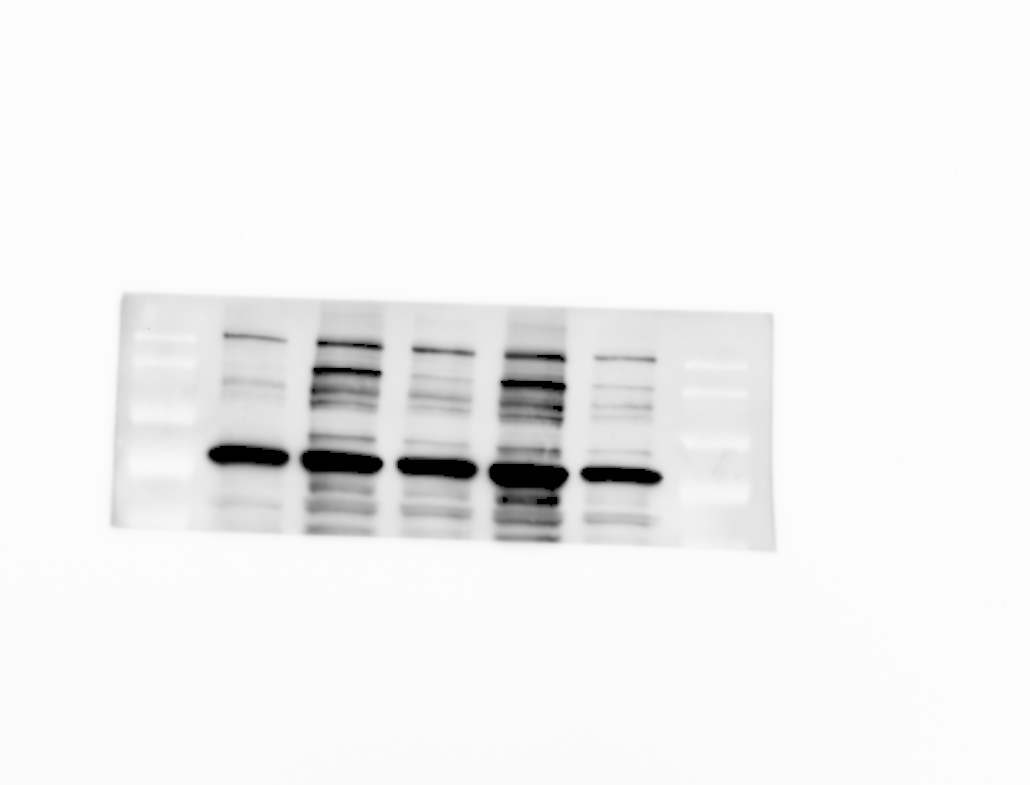

Supplement: Supplementary file 3 [file DataSheet4.ZIP › Raw data-3/Western Blot/in vitro/mTORC1/mTORC1(3).tif]

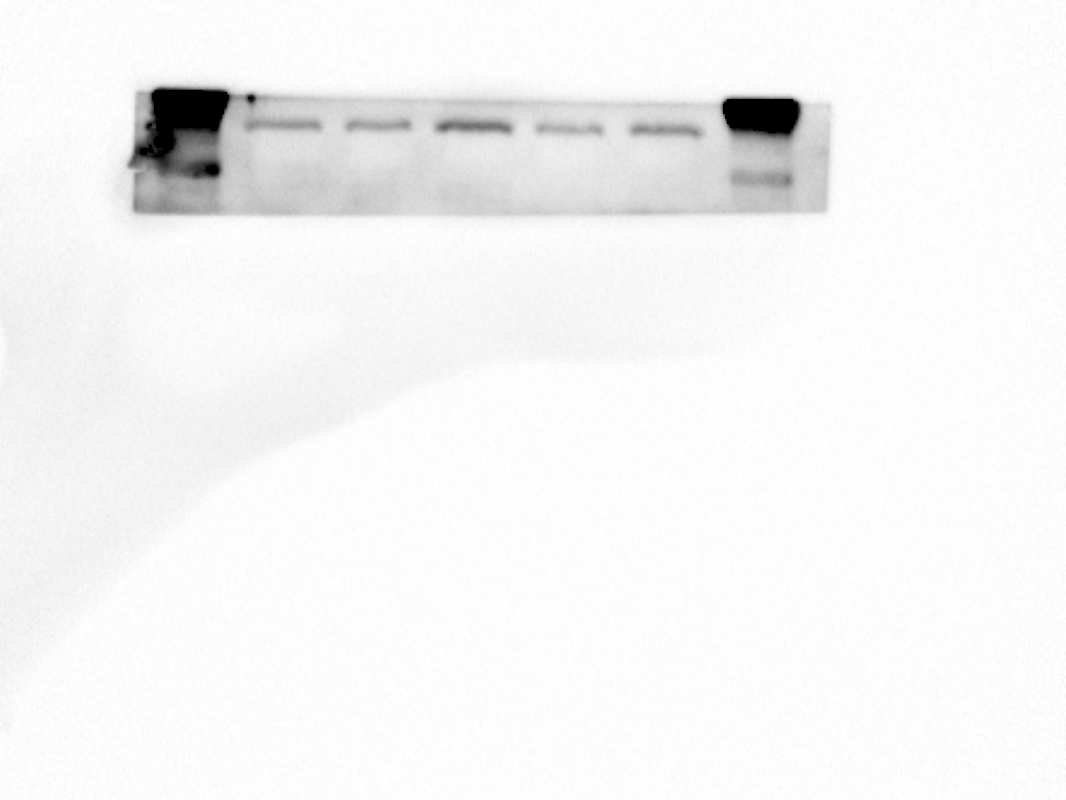

Supplement: Supplementary file 3 [file DataSheet4.ZIP › Raw data-3/Western Blot/in vitro/p-Akt/p-Akt(1).tif]

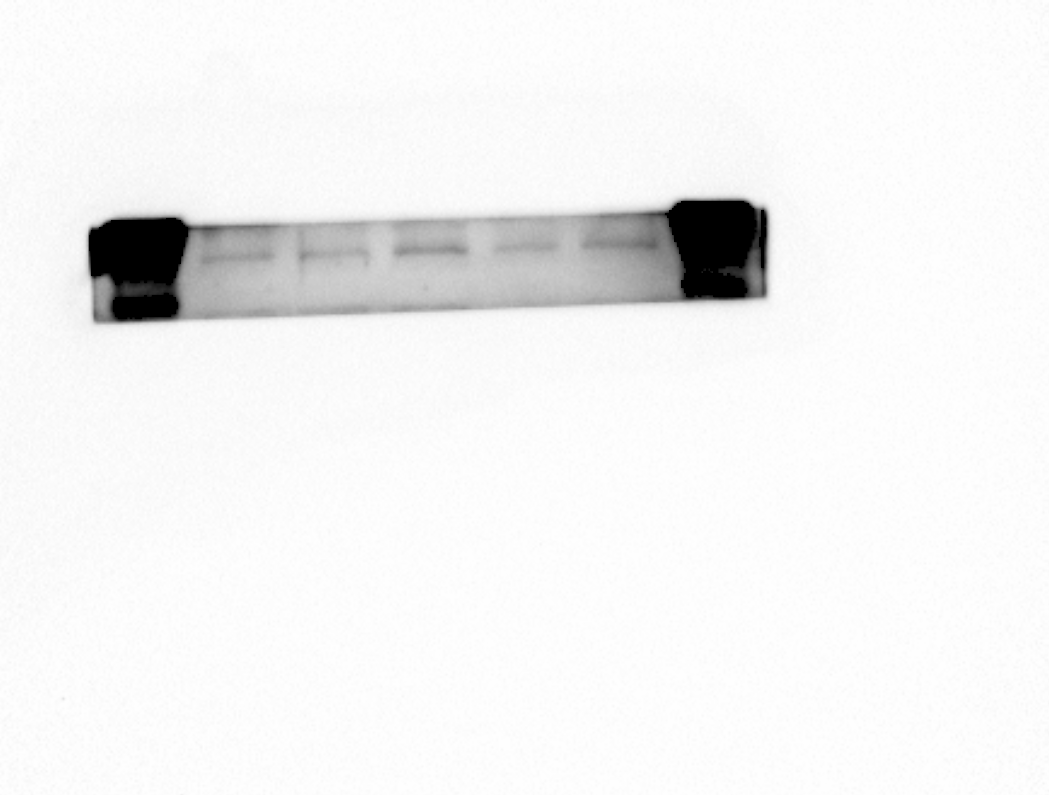

Supplement: Supplementary file 3 [file DataSheet4.ZIP › Raw data-3/Western Blot/in vitro/p-Akt/p-Akt(2).tif]

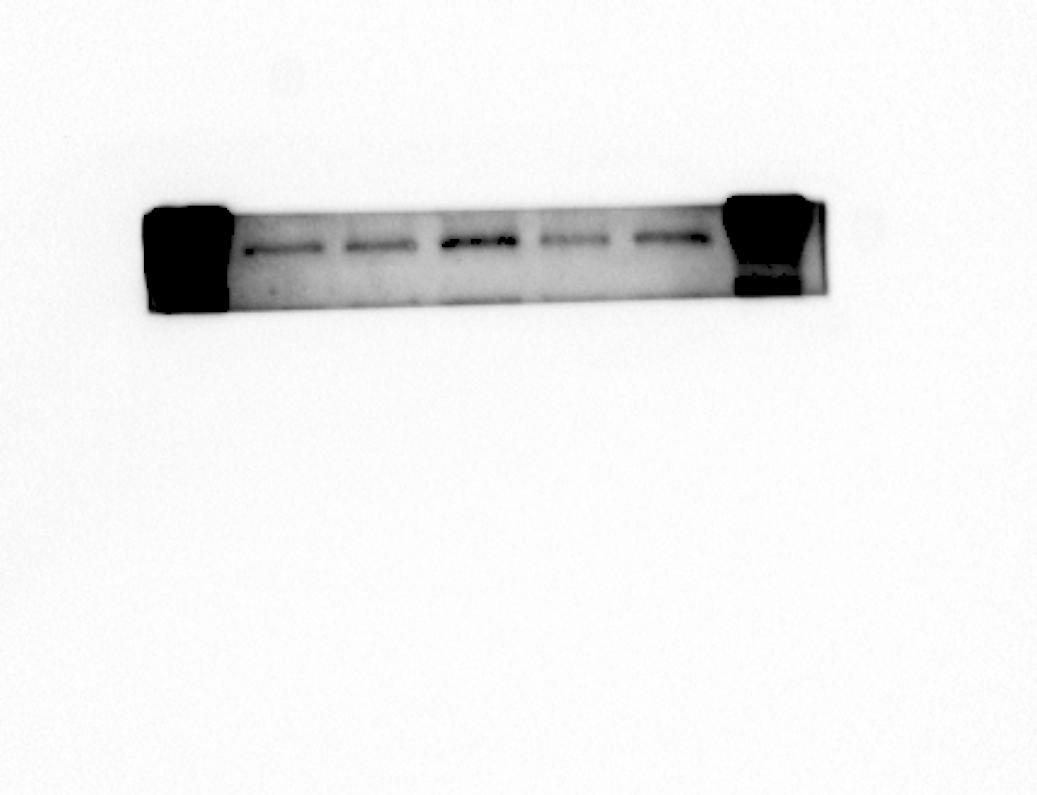

Supplement: Supplementary file 3 [file DataSheet4.ZIP › Raw data-3/Western Blot/in vitro/p-Akt/p-Akt(3).tif]
