# Supplementary material for: Shexiang Tongxin Dropping Pills Promote Macrophage Polarization-Induced Angiogenesis Against Coronary Microvascular Dysfunction via PI3K/Akt/mTORC1 Pathway
Source: Front Pharmacol. 2022 Mar 23;13:840521. doi: 10.3389/fphar.2022.840521 (PMC8984141; doi:10.3389/fphar.2022.840521)
Supplement: Supplementary file 2 [file Presentation1.pdf]

Supplementary Material

Supplementary Figures

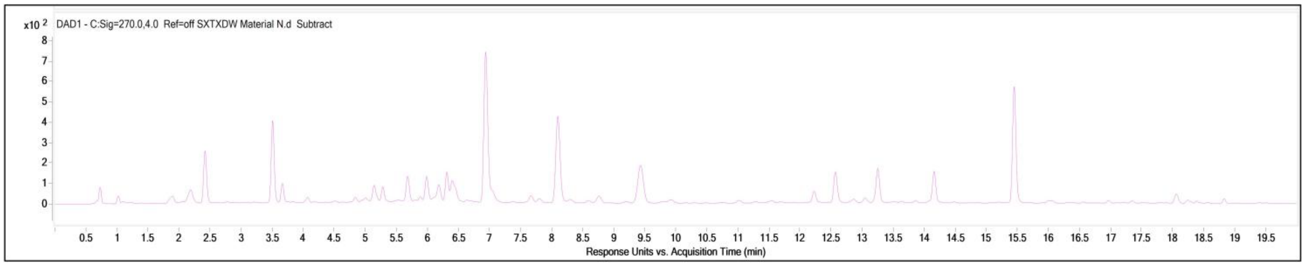

Supplementary Figure 1. The fingerprint was established by HPLC to control the quality of STDP.

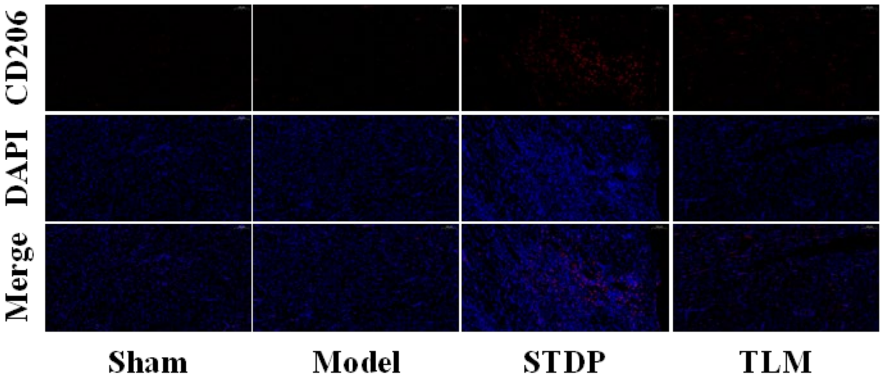

Supplementary Figure 2. IF staining of CD206 in different groups .
