# Supplementary figures and images for: Shexiang Tongxin Dropping Pills Promote Macrophage Polarization-Induced Angiogenesis Against Coronary Microvascular Dysfunction via PI3K/Akt/mTORC1 Pathway (part 2 of 2)
Source: Front Pharmacol. 2022 Mar 23;13:840521. doi: 10.3389/fphar.2022.840521 (PMC8984141; doi:10.3389/fphar.2022.840521)

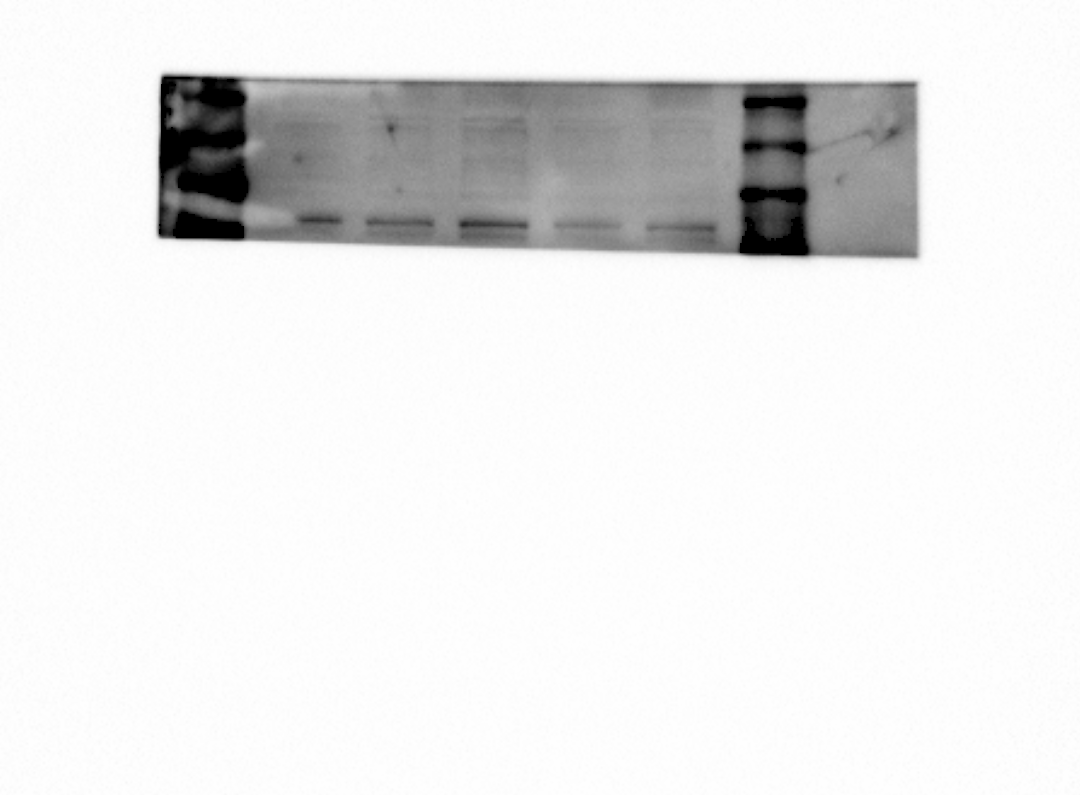

Supplement: Supplementary file 3 [file DataSheet4.ZIP › Raw data-3/Western Blot/in vitro/P-PI3K/P-PI3K(1).tif]

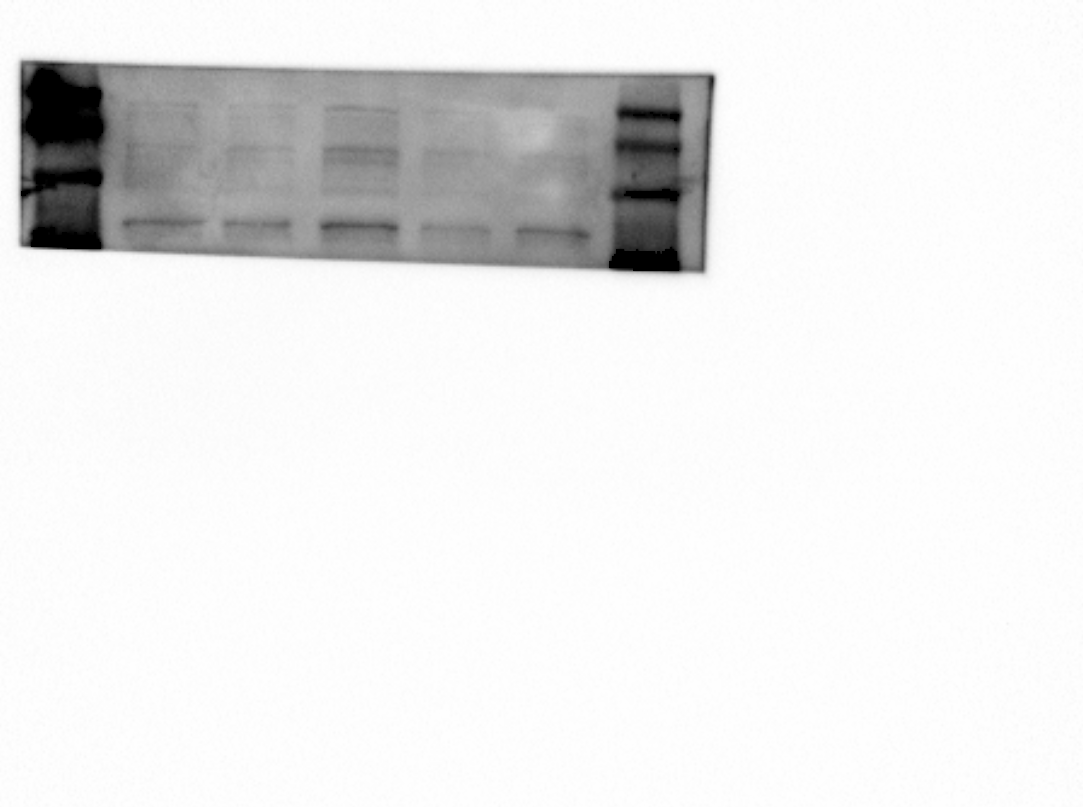

Supplement: Supplementary file 3 [file DataSheet4.ZIP › Raw data-3/Western Blot/in vitro/P-PI3K/P-PI3K(2).tif]

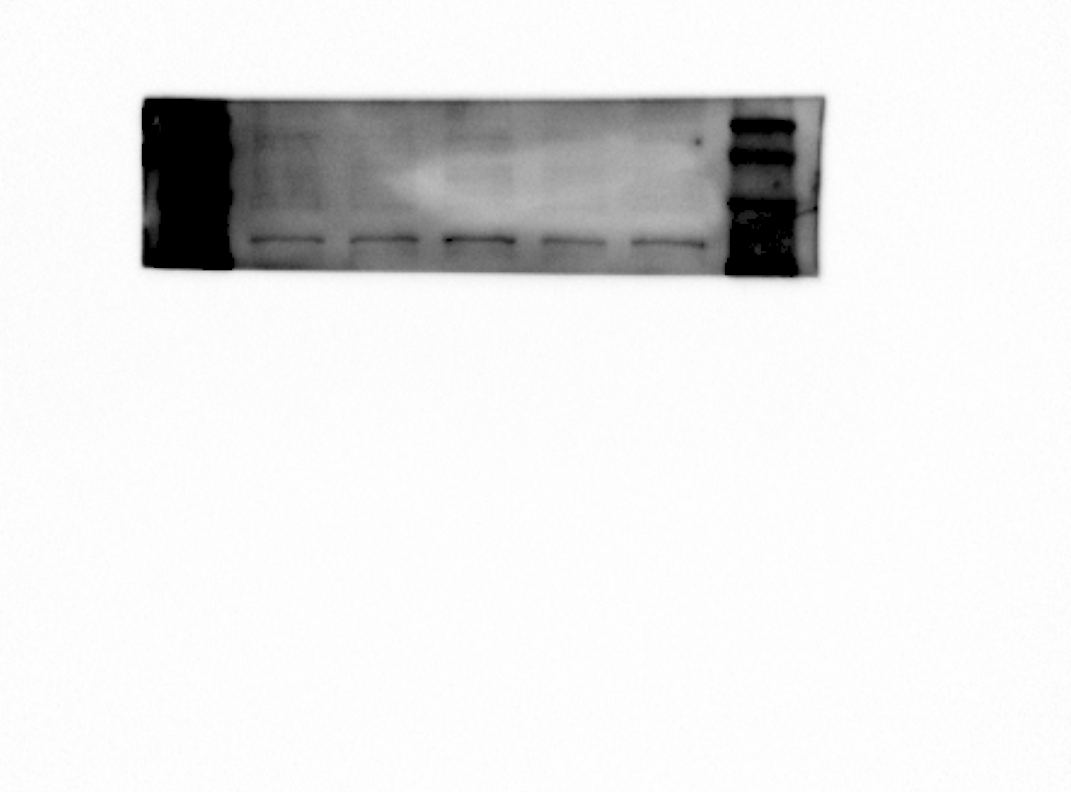

Supplement: Supplementary file 3 [file DataSheet4.ZIP › Raw data-3/Western Blot/in vitro/P-PI3K/P-PI3K(3).tif]

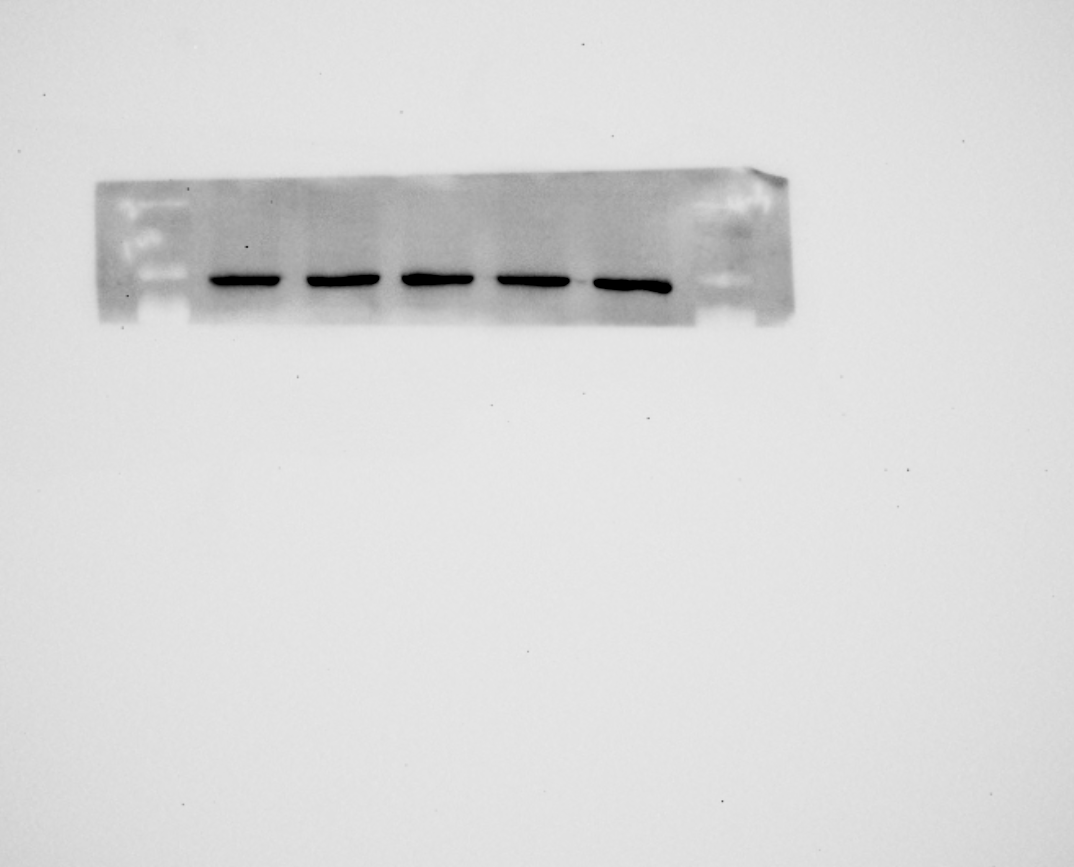

Supplement: Supplementary file 3 [file DataSheet4.ZIP › Raw data-3/Western Blot/in vitro/PI3K/PI3K(1).tif]

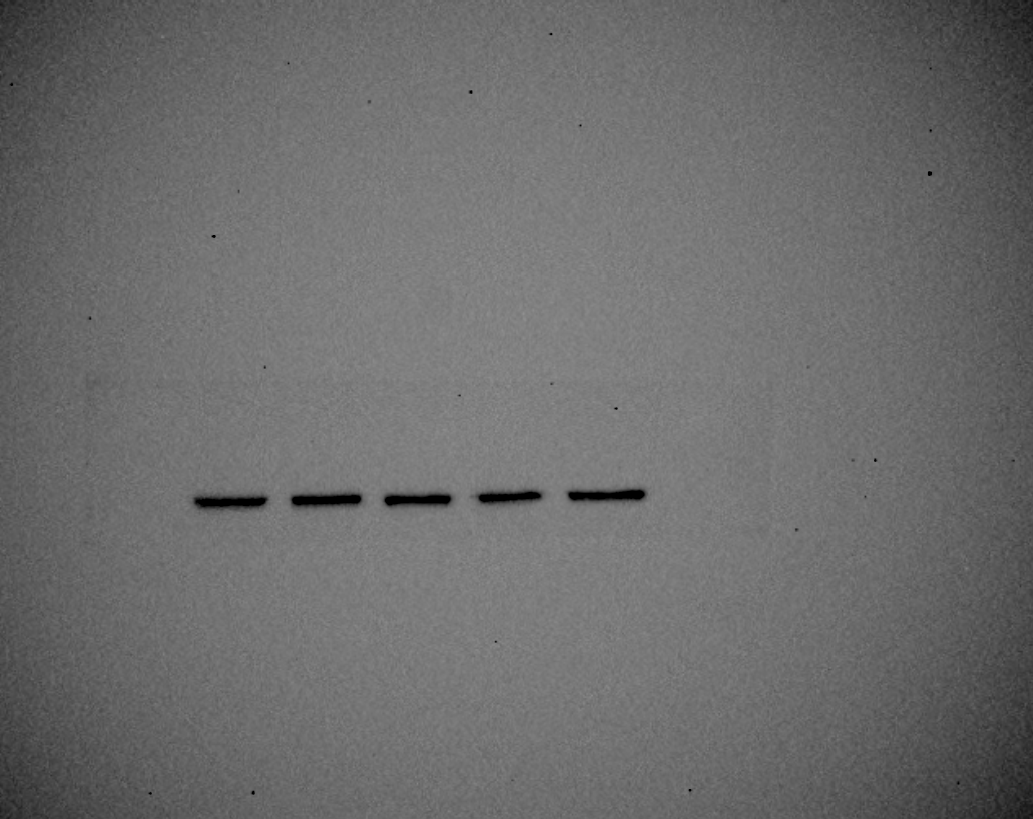

Supplement: Supplementary file 3 [file DataSheet4.ZIP › Raw data-3/Western Blot/in vitro/PI3K/PI3K(2).tif]

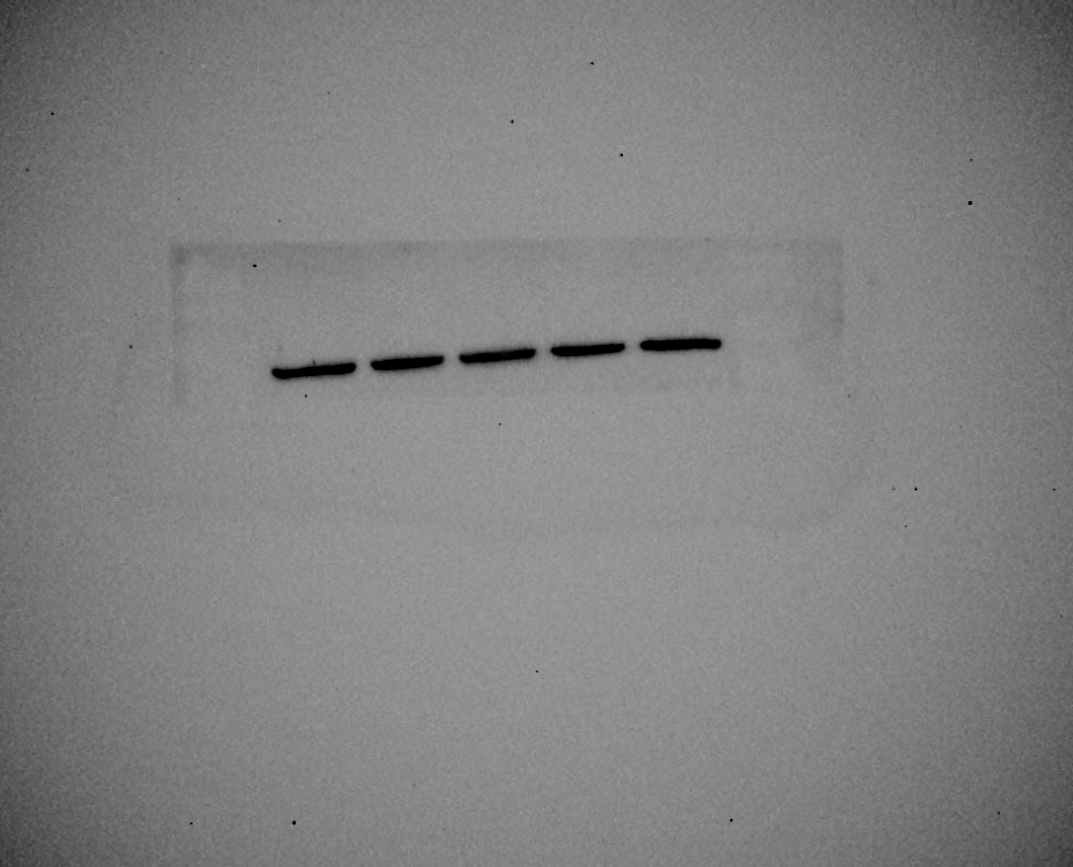

Supplement: Supplementary file 3 [file DataSheet4.ZIP › Raw data-3/Western Blot/in vitro/PI3K/PI3K(3).tif]

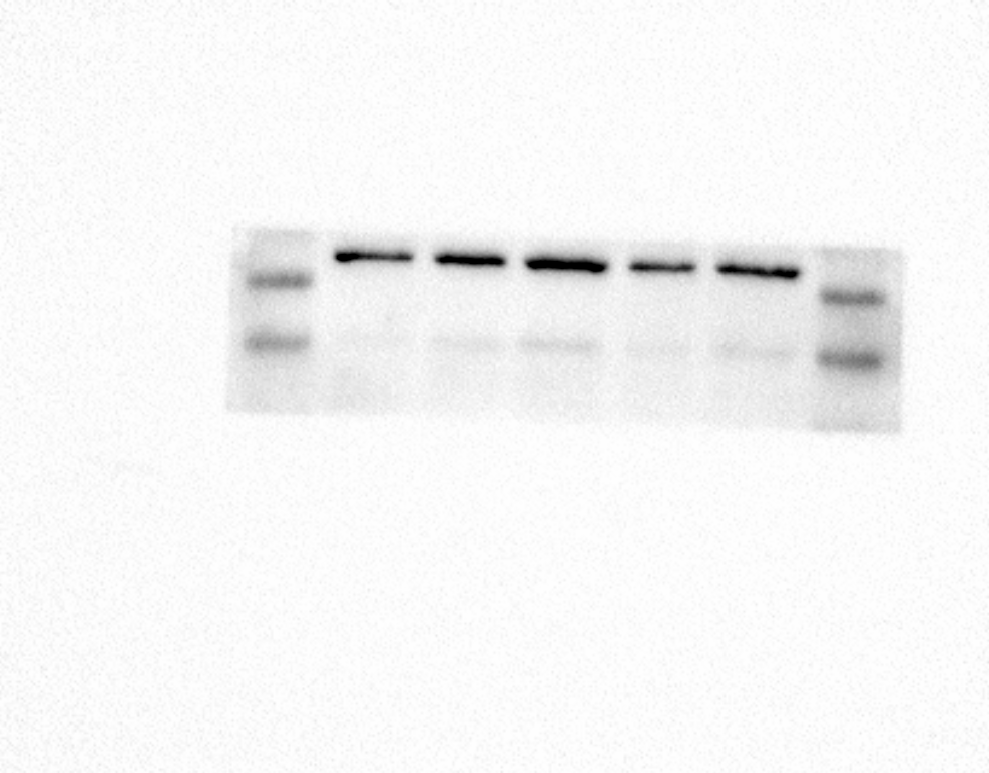

Supplement: Supplementary file 3 [file DataSheet4.ZIP › Raw data-3/Western Blot/in vitro/VEGF-A/VEGF-A(1).tif]

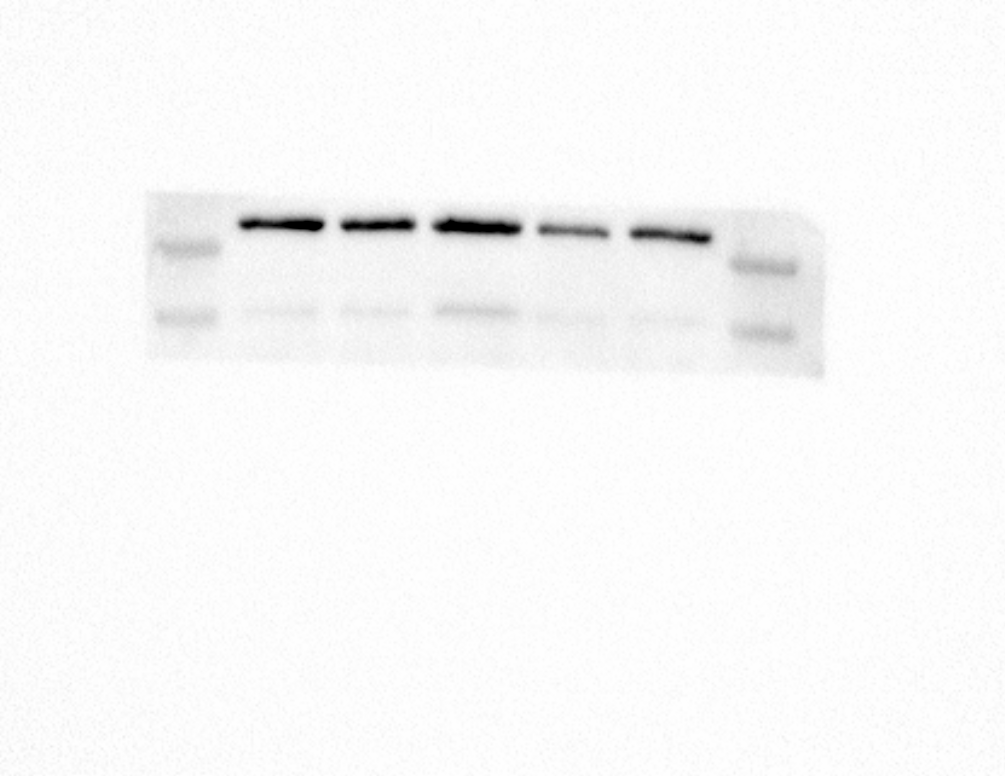

Supplement: Supplementary file 3 [file DataSheet4.ZIP › Raw data-3/Western Blot/in vitro/VEGF-A/VEGF-A(2).tif]

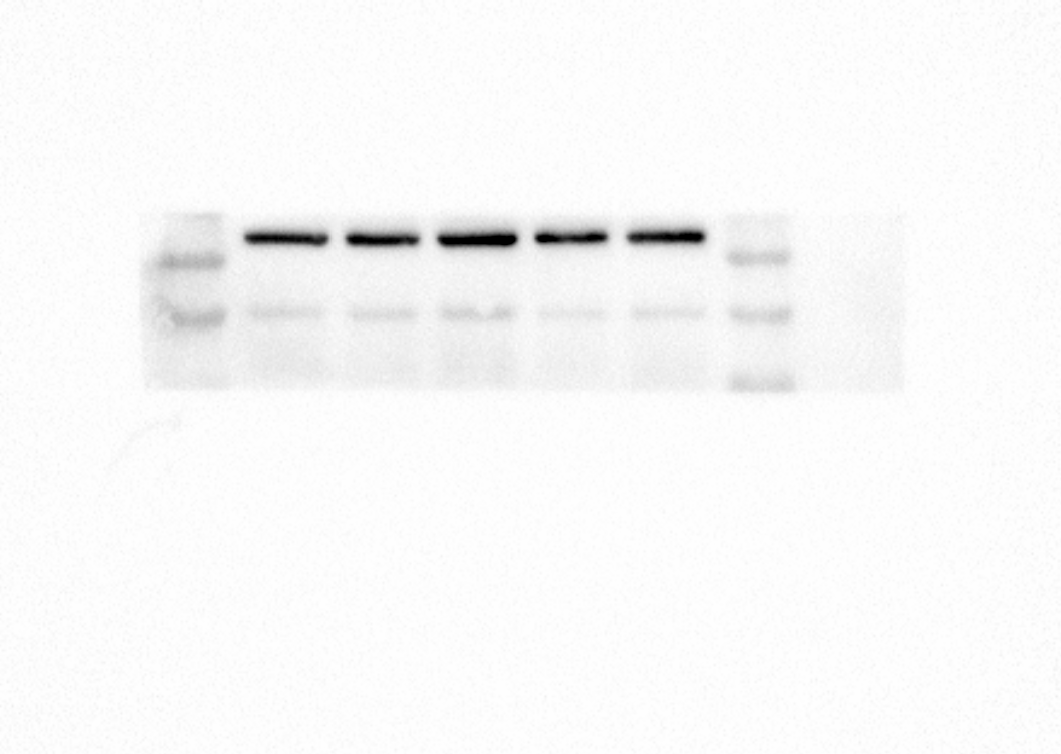

Supplement: Supplementary file 3 [file DataSheet4.ZIP › Raw data-3/Western Blot/in vitro/VEGF-A/VEGF-A(3).tif]

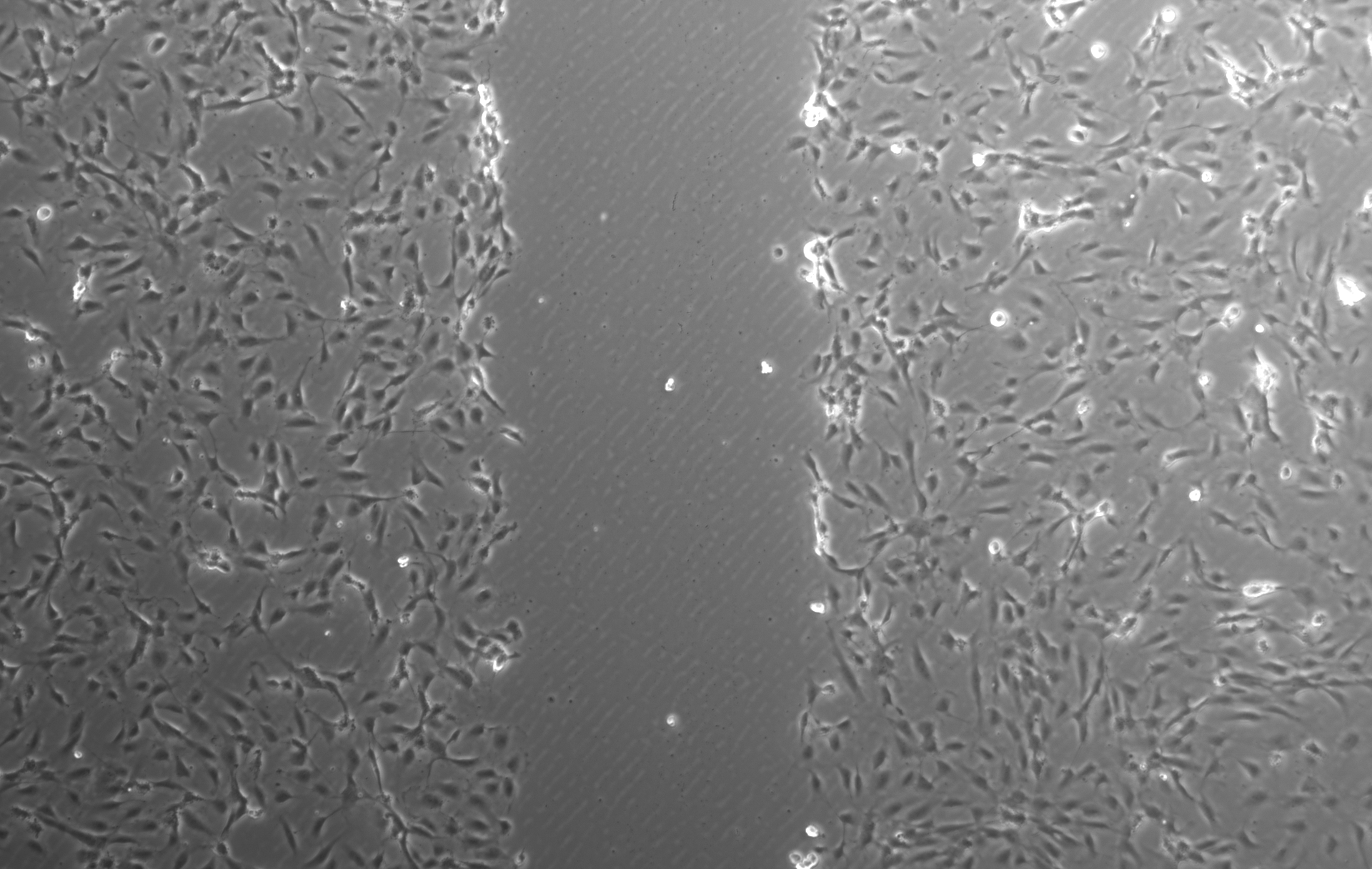

Supplement: Supplementary file 4 [file DataSheet1.ZIP › Raw data-1-1/Wound Healing Assay/0h/HUVEC-Control.tif]

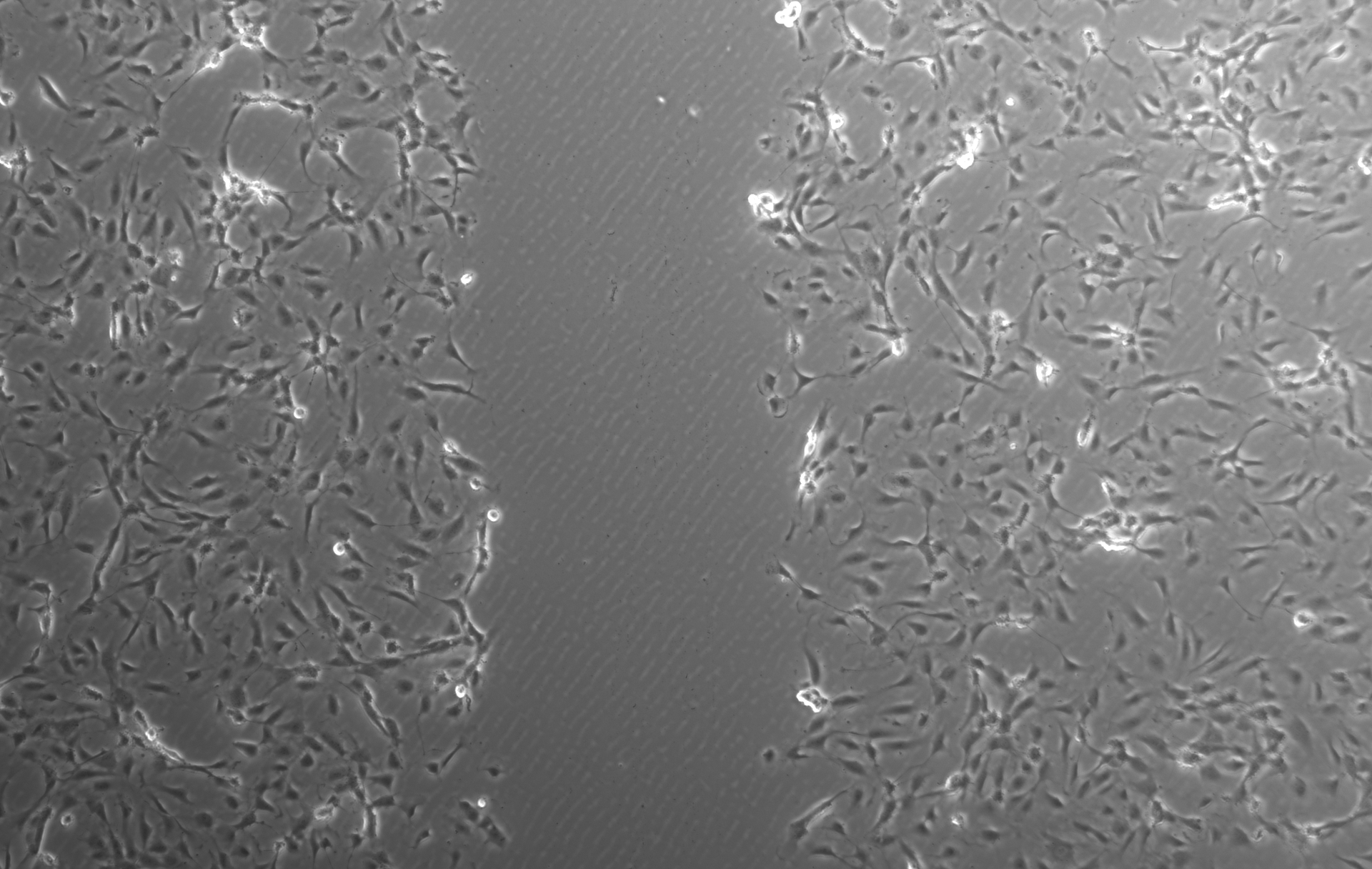

Supplement: Supplementary file 4 [file DataSheet1.ZIP › Raw data-1-1/Wound Healing Assay/0h/HUVEC-Model.tif]

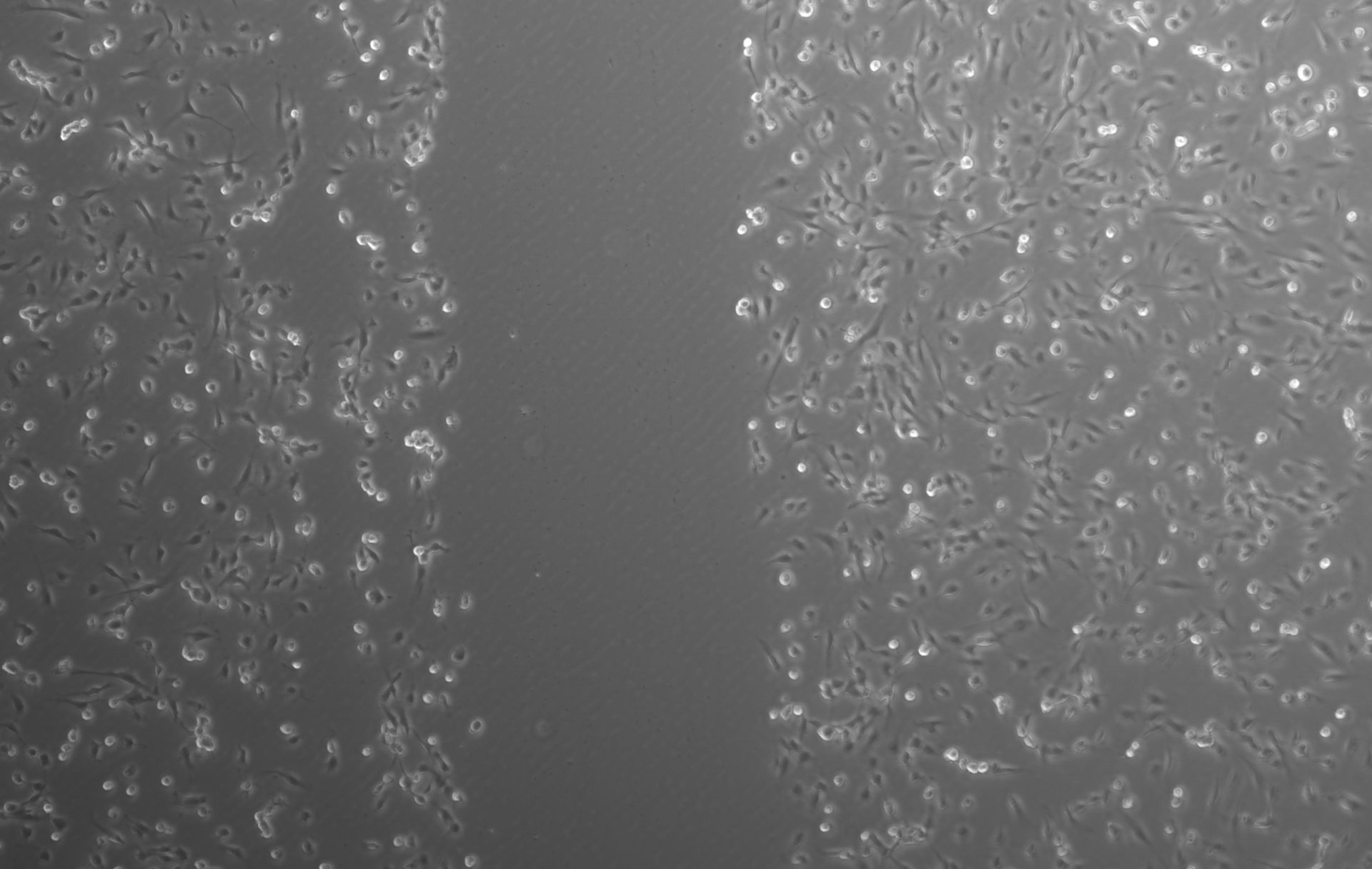

Supplement: Supplementary file 4 [file DataSheet1.ZIP › Raw data-1-1/Wound Healing Assay/0h/HUVEC-STDP.tif]

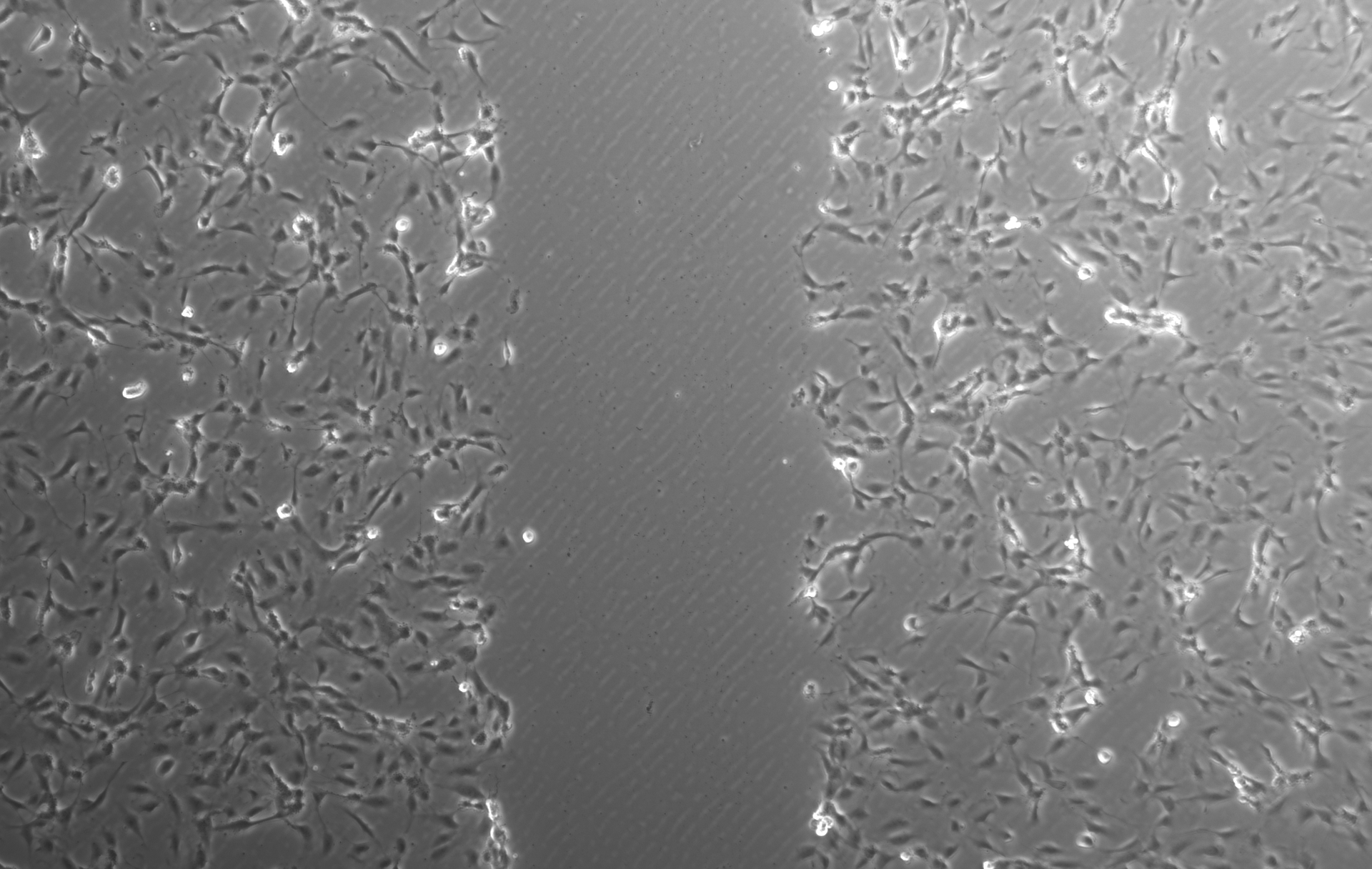

Supplement: Supplementary file 4 [file DataSheet1.ZIP › Raw data-1-1/Wound Healing Assay/0h/HUVEC-VEGF-A.tif]

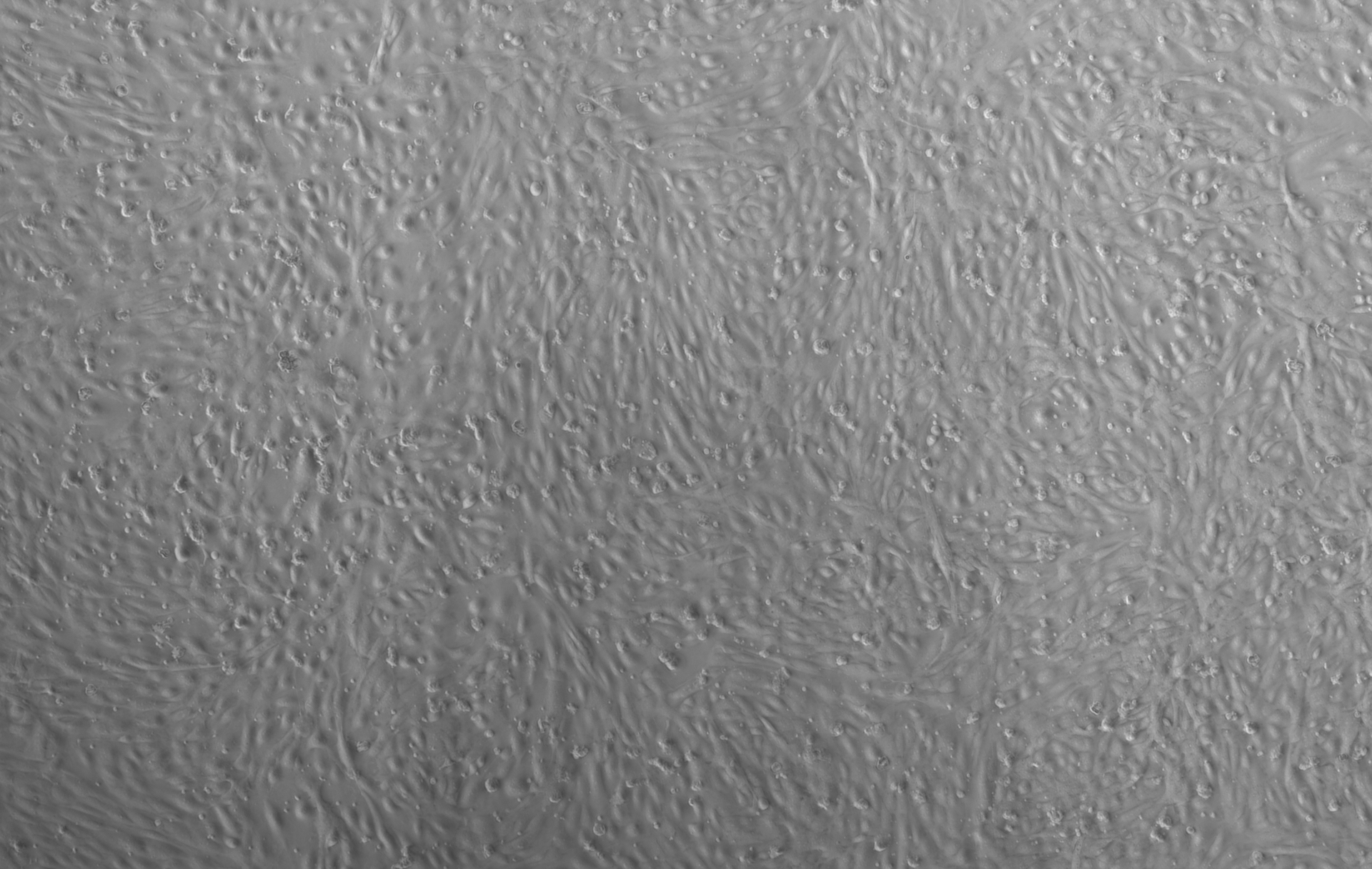

Supplement: Supplementary file 4 [file DataSheet1.ZIP › Raw data-1-1/Wound Healing Assay/12h/Control-12h.tif]

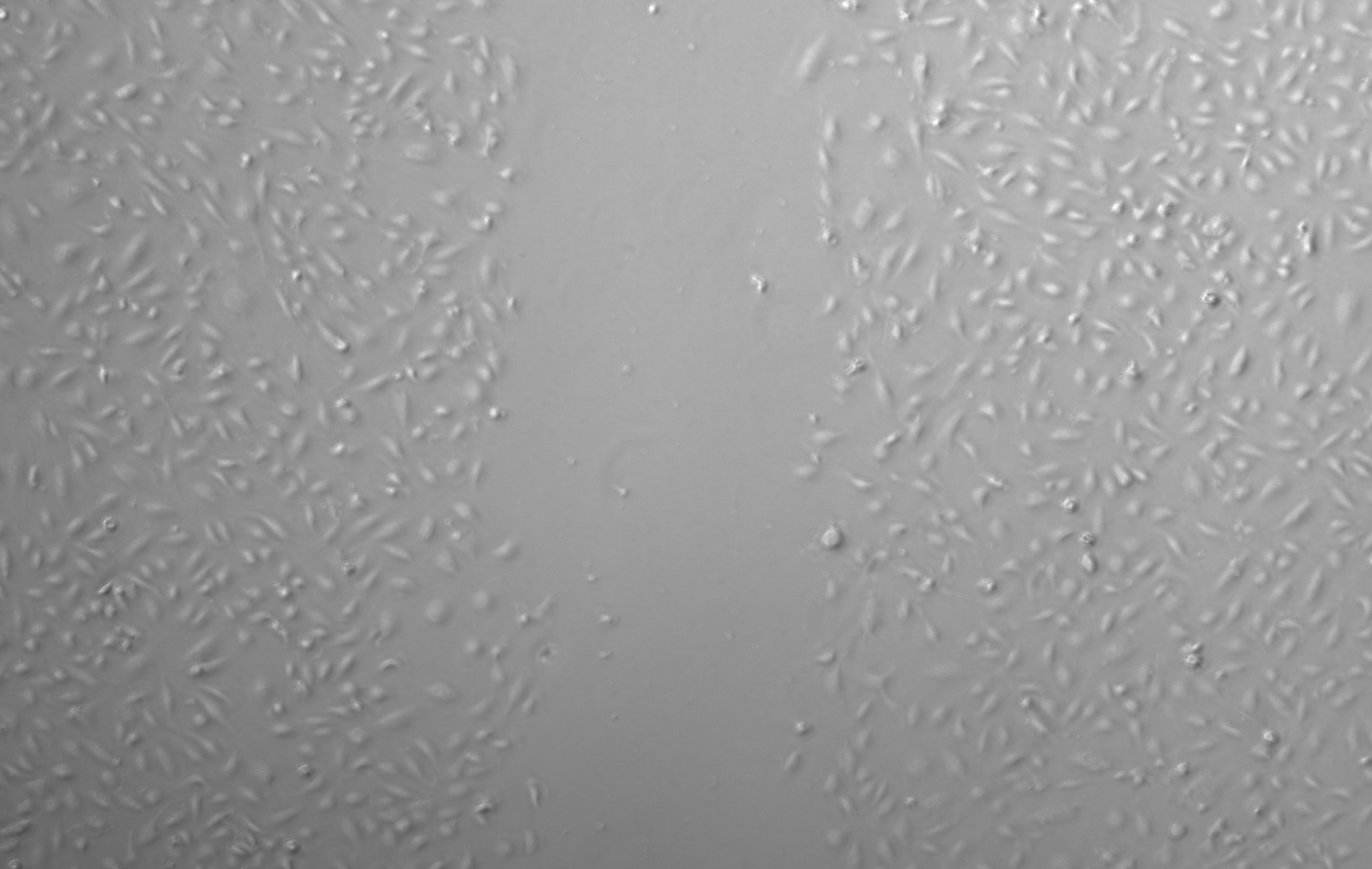

Supplement: Supplementary file 4 [file DataSheet1.ZIP › Raw data-1-1/Wound Healing Assay/12h/Model-12h.tif]

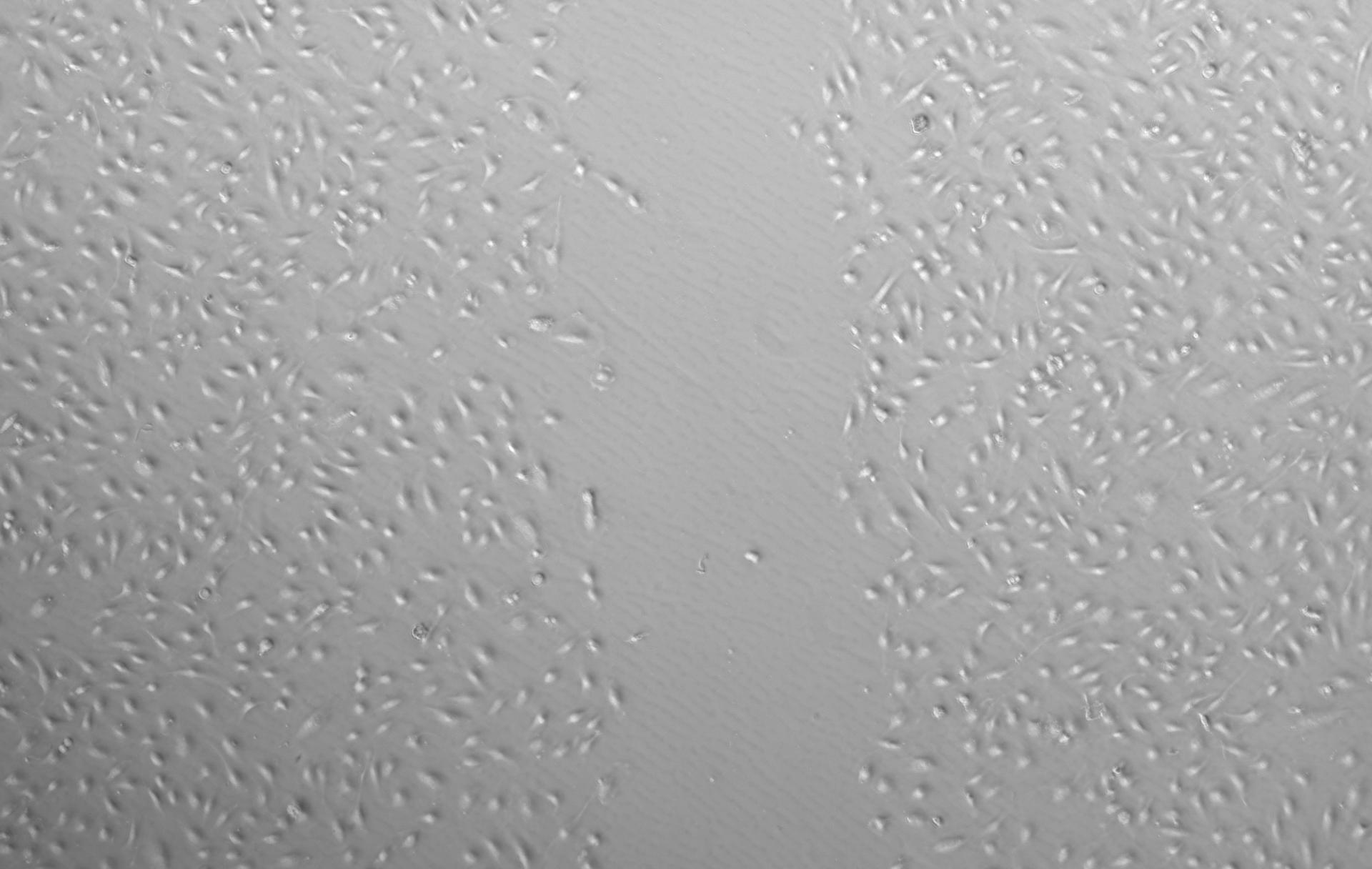

Supplement: Supplementary file 4 [file DataSheet1.ZIP › Raw data-1-1/Wound Healing Assay/12h/STDP-12h.tif]

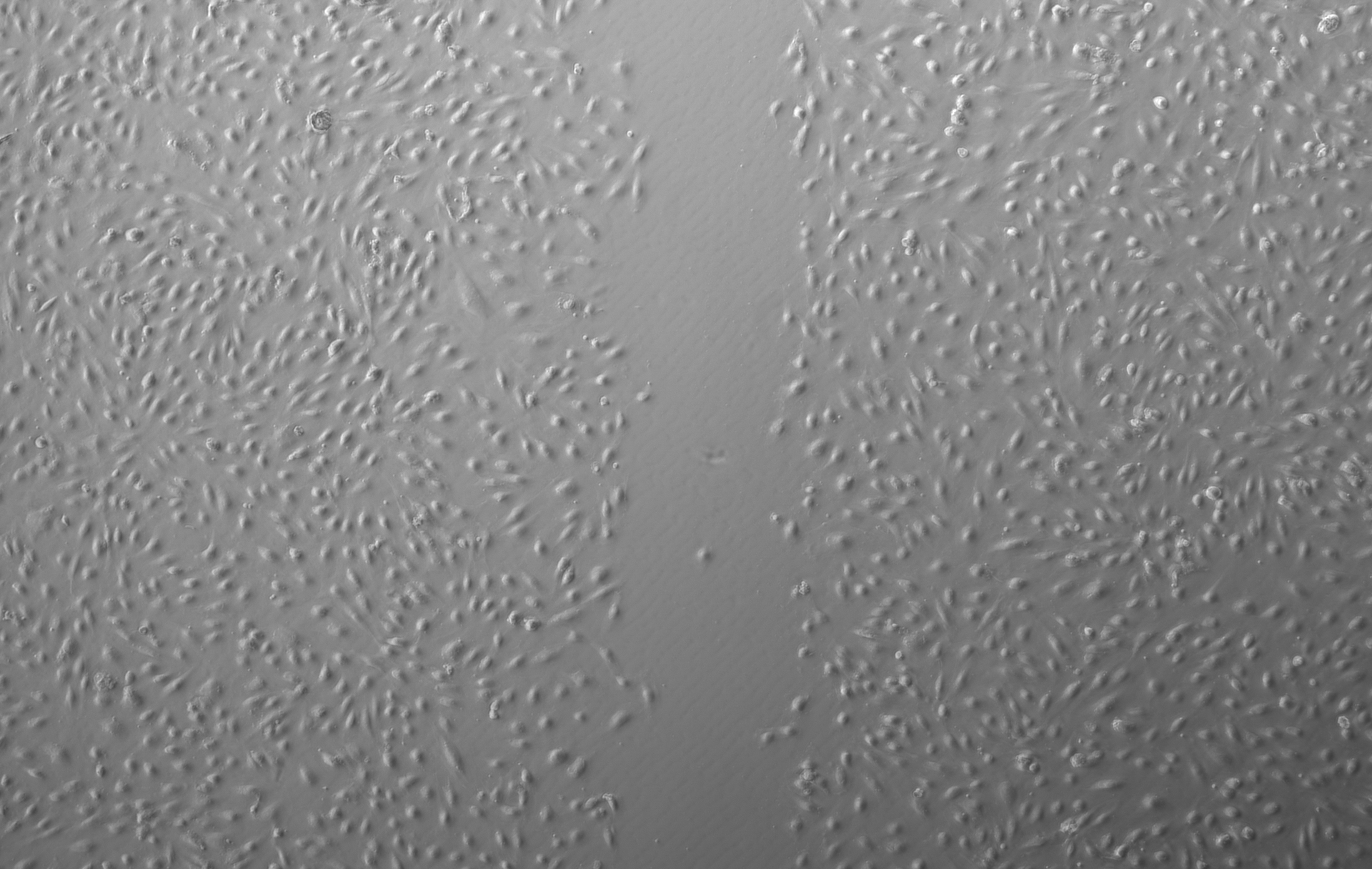

Supplement: Supplementary file 4 [file DataSheet1.ZIP › Raw data-1-1/Wound Healing Assay/12h/VEGFA-12h.tif]

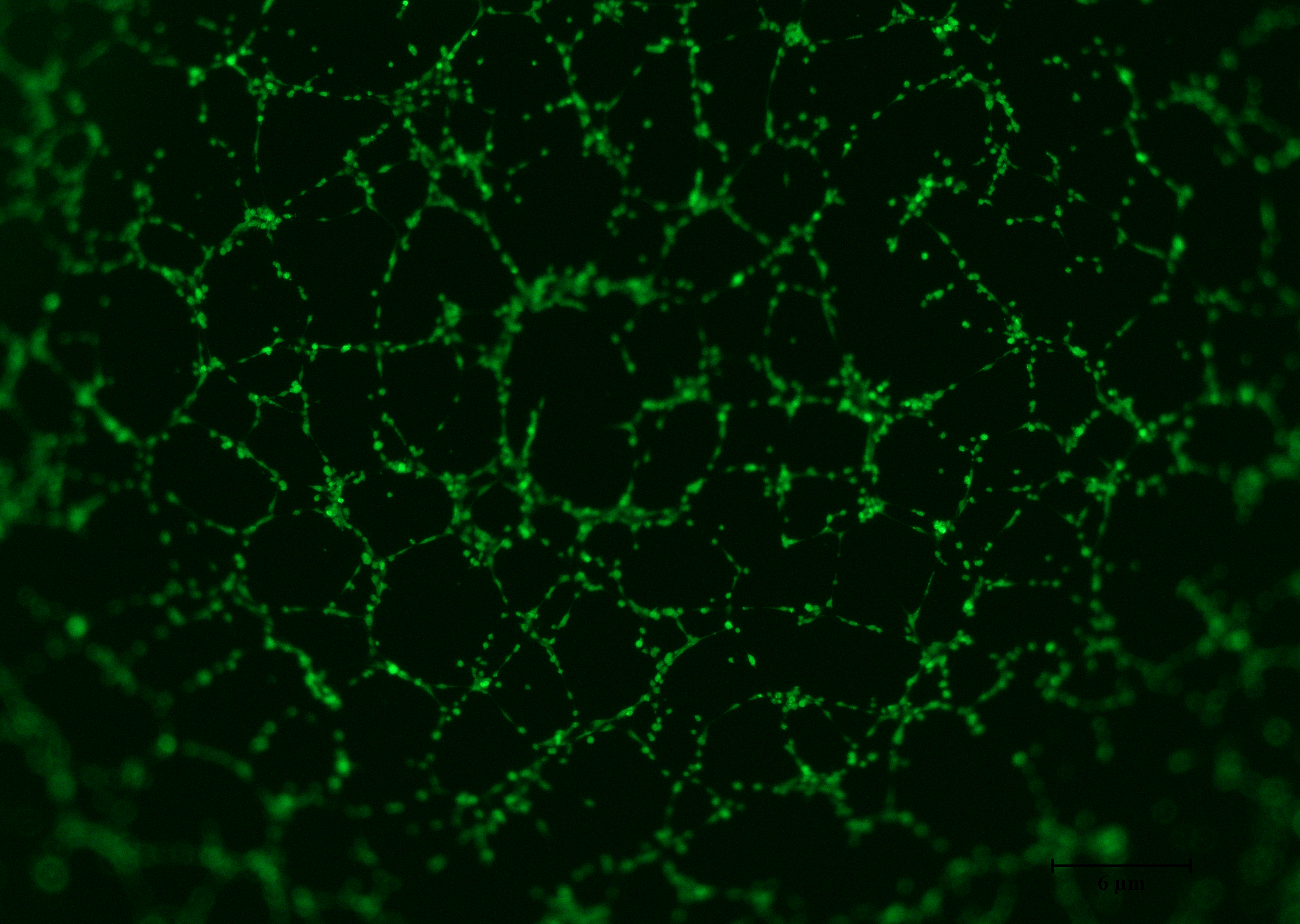

Supplement: Supplementary file 5 [file DataSheet2.ZIP › Raw data-1-2/Matrigel Tube Formation Assay/HUVEC-Control.tif]

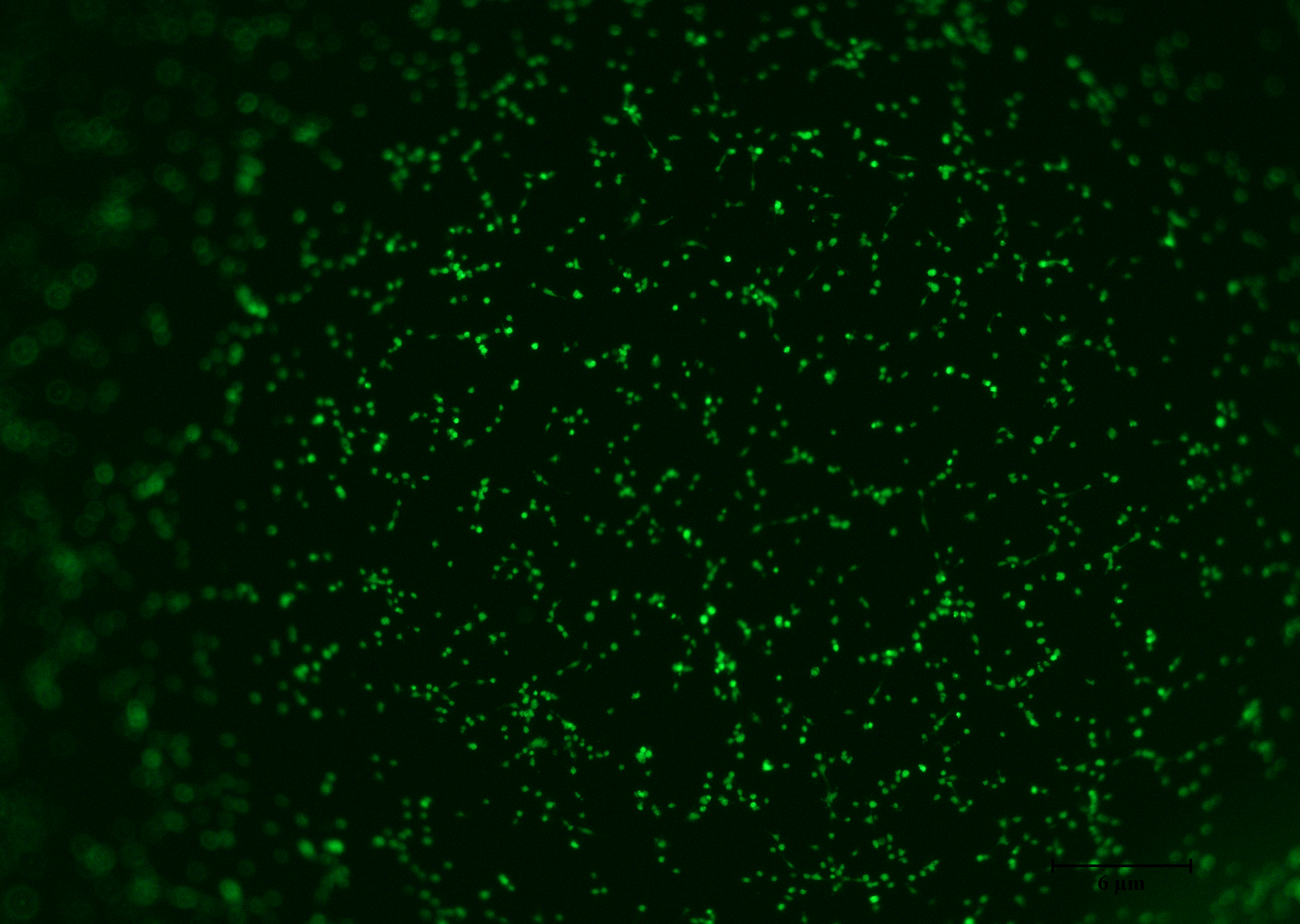

Supplement: Supplementary file 5 [file DataSheet2.ZIP › Raw data-1-2/Matrigel Tube Formation Assay/HUVEC-Model.tif]

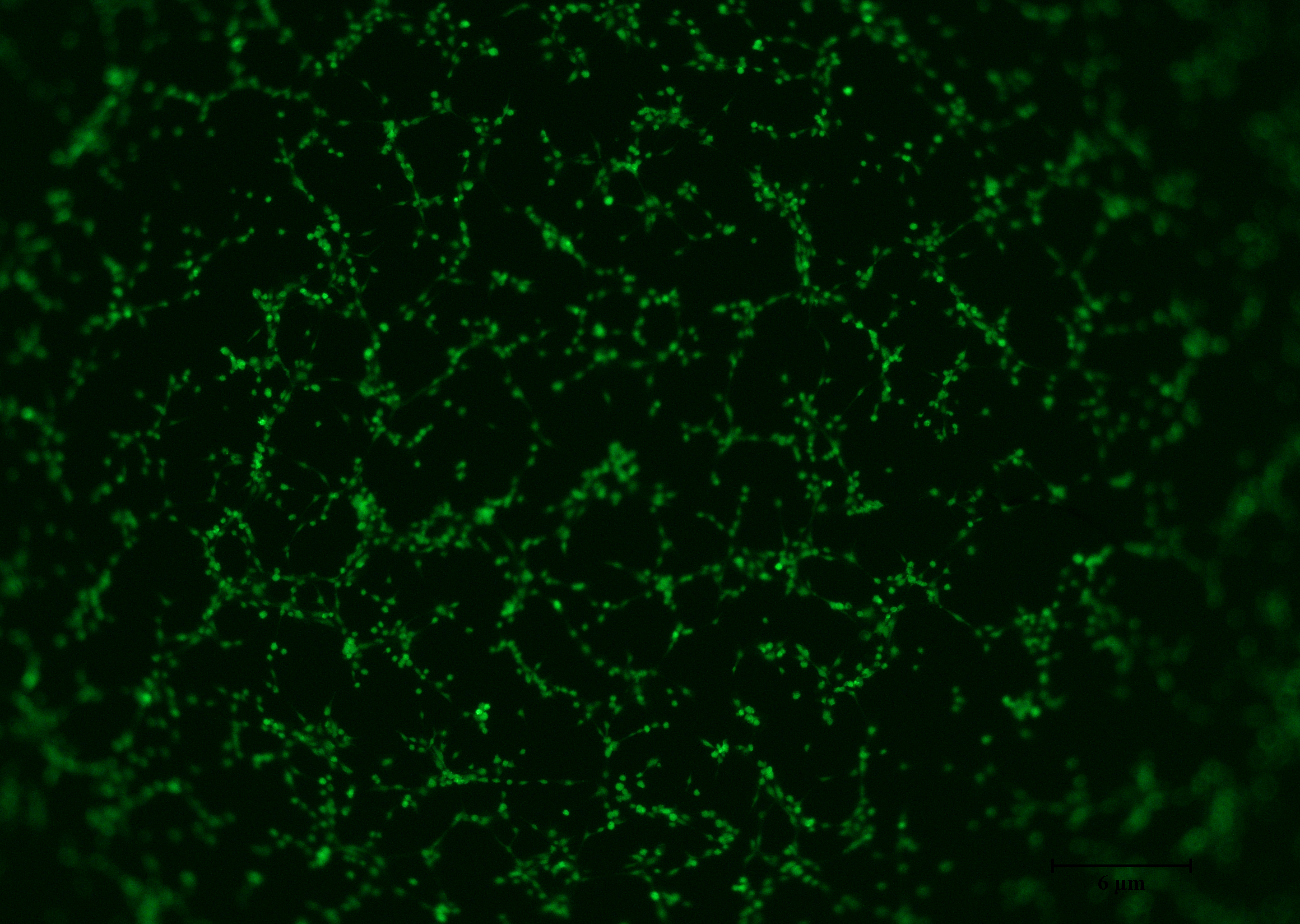

Supplement: Supplementary file 5 [file DataSheet2.ZIP › Raw data-1-2/Matrigel Tube Formation Assay/HUVEC-STDP.tif]

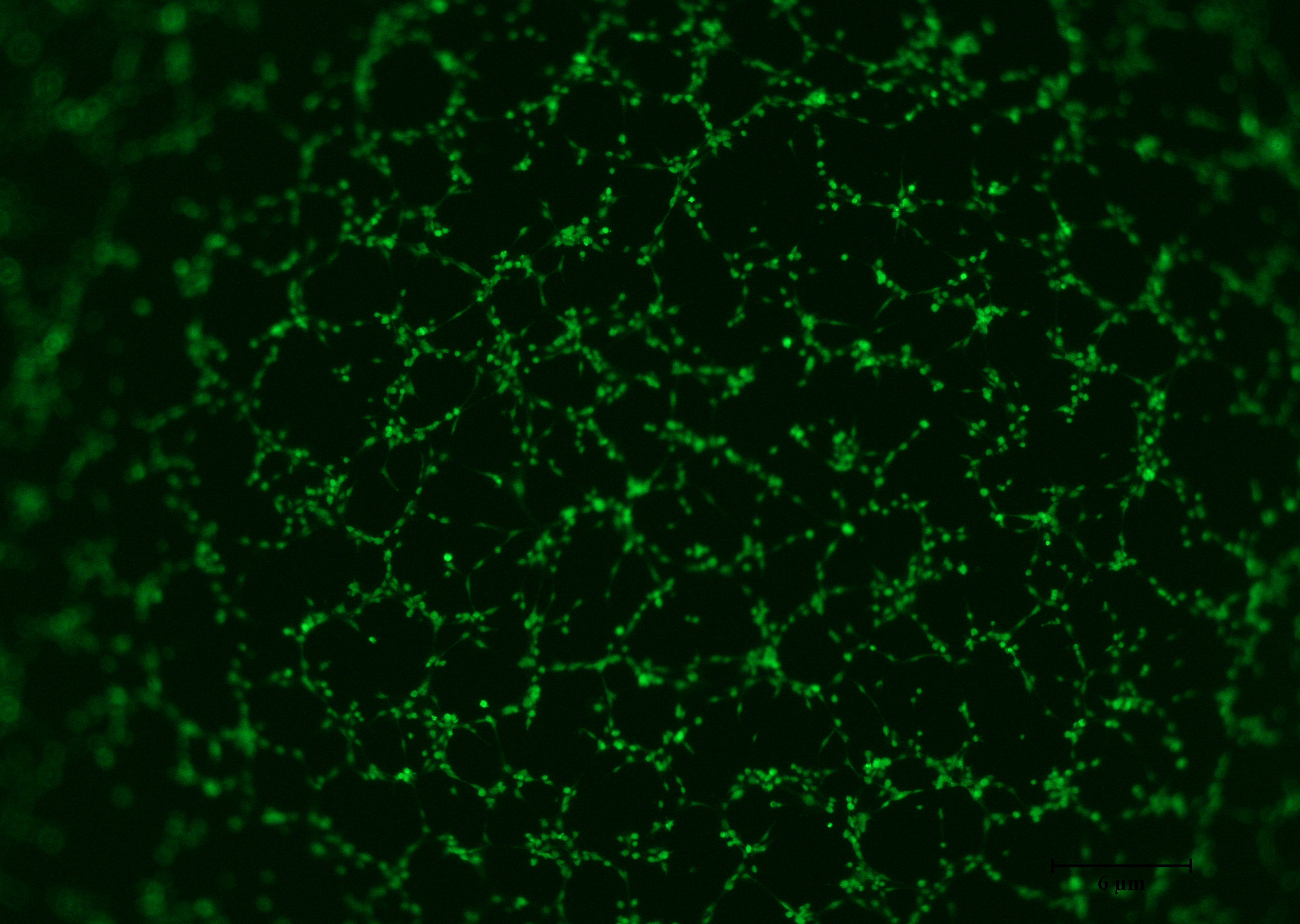

Supplement: Supplementary file 5 [file DataSheet2.ZIP › Raw data-1-2/Matrigel Tube Formation Assay/HUVEC-VEGFA.tif]
